# In your face: the biased judgement of fear-anger expressions in violent offenders

**DOI:** 10.1186/s40359-017-0186-z

**Published:** 2017-05-12

**Authors:** Martin Wegrzyn, Sina Westphal, Johanna Kissler

**Affiliations:** 1 Department of Psychology, Bielefeld University, Postfach 10 01 31, 33501 Bielefeld, Germany; 2 Center of Excellence Cognitive Interaction Technology (CITEC), Bielefeld University, Bielefeld, Germany

**Keywords:** Emotion, Face recognition, Psychopathology, Aggression, Psychophysics

## Abstract

**Background:**

Why is it that certain violent criminals repeatedly find themselves engaged in brawls? Many inmates report having felt provoked or threatened by their victims, which might be due to a tendency to ascribe malicious intentions when faced with ambiguous social signals, termed hostile attribution bias.

**Methods:**

The present study presented morphed fear-anger faces to prison inmates with a history of violent crimes, a history of child sexual abuse, and to matched controls form the general population. Participants performed a fear-anger decision task. Analyses compared both response frequencies and measures derived from psychophysical functions fitted to the data. In addition, a test to distinguish basic facial expressions and questionnaires for aggression, psychopathy and personality disorders were administered.

**Results:**

Violent offenders present with a reliable hostile attribution bias, in that they rate ambiguous fear-anger expressions as more angry, compared to both the control population and perpetrators of child sexual abuse. Psychometric functions show a lowered threshold to detect anger in violent offenders compared to the general population. This effect is especially pronounced for male faces, correlates with self-reported aggression and presents in absence of a general emotion recognition impairment.

**Conclusions:**

The results indicate that a hostile attribution, related to individual level of aggression and pronounced for male faces, might be one mechanism mediating physical violence.

**Electronic supplementary material:**

The online version of this article (doi:10.1186/s40359-017-0186-z) contains supplementary material, which is available to authorized users.

## Background

What characterizes inmates who have been found guilty of violent offences and what is it that distinguishes them from other groups of criminals or from the population at large? While most of us manage to go through life without having inflicted physical harm unto others, violent offenders usually report a history of repeated engagement in brawls. Anecdotally, they often report feeling provoked or threatened by their respective victims, an assessment which calls for scepticism, as there is evidence that this stems at least partly from an inaccurate perception of social signals: Far from being just inaccurate, this perception rather seems skewed in one direction, in what is termed hostile attribution bias [1–3]. This bias is defined as the tendency to attribute malicious intentions to an interaction partner, even in absence of any clear stimuli that would justify such an attribution [3–5]. This hostile attribution bias has been identified in violent offenders, for example by performing tests with semi-projective stories or ratings of body postures, which these groups of delinquents often identify as more hostile than do non-violent comparison groups [6].

Since the face is one of the most important cues in social interaction, there has also been accumulating evidence that the hostile attribution bias leads to a characteristic misperception of facial expressions. For example, inmates diagnosed with antisocial personality disorder or psychopathy have been found to show deficits in emotion expression recognition [7–9]. While hostile intentions could in theory be ascribed to any ambiguous facial expression, the bias seems to be triggered most strongly when the expression contains some amount of anger [10].

A number of studies tapping into the hostile attribution bias have used gradually morphed faces, generating a continuum from one expression (e.g. full-blown fear) to another (e.g. full-blown anger), with ambiguous faces (half-fearful, half-angry) in the middle of the spectrum [11, 12].

For example, when a face is gradually morphed from a fearful to an angry expression, violent offenders have been found to respond to the faces in the middle of the spectrum (where guessing is the only viable strategy for an unbiased observer), with a marked anger bias [12]. Meanwhile, their perception of morphed faces not containing anger (e.g. happy-fearful morphs), seems not biased in any way, which indicates a more specific deficit. The anger bias for ambiguous faces has been found repeatedly with different variations of morphed faces and different groups of violent offenders, such as adolescents with a history of criminal offending [13], adult delinquents with antisocial personality disorder [14] and violent offenders without a clinical diagnosis [6]. Furthermore, some studies found a dissociation of responses to male and female faces, with more pronounced hostile attributions for male faces or postures [6, 15]. However, no study so far compared violence offenders to groups of other inmates. Therefore, the specificity of a hostile attribution bias for this type of criminal offenders remains an open question. If hostile attributions are specific for aggressive behaviour, they should for example not be present in child sex offenders, who are known to be low in empathy [16], but whose abusive behaviour is often not overtly violent.

While evidence whether the anger bias correlates with self-report measures of aggression is mixed [10, 17–19] this also indicates that a pattern of hostile attributions for faces might tap into mechanisms that are independent of or not easily assessed with questionnaire measures. Also, different types of aggression exist, such as appetitive aggression, associated with gaining pleasure form harming others and facilitative aggression, associated with the reduction of unpleasant states [20]. Hence, the hostile attribution bias might be associated only with certain kinds of aggression.

The mechanisms behind the hostile attribution bias might be further elucidated by using methods from psychophysics allowing to characterize observers’ responses in greater detail. Basic research has shown that when participants are asked to identify morphed faces as fearful or angry, their responses do not follow the linear changes in low-level features of the face, but reflect a categorization into distinct groups [21, 22]. This categorical perception is reflected in an s-shaped response function, which indicates a sharp shift from perceiving one expression to perceiving the other [23, 24]. It might therefore be expected that individuals exhibiting a hostile attribution bias will show anomalous categorical perception, with the category boundary shifted such that anger is perceived earlier. Changes in categorical perception specific to faces containing anger have been shown in groups of children with a history of physical abuse [11, 25] and might be similarly present in violent offenders reflecting the above mentioned hostile attribution bias or as a correlate of higher levels of aggression. A deeper understanding of the biased perception of facial signals in violent offenders might help understand some aspects of how delinquents perceive social signals and tailor specific interventions to overcome this bias [13, 26].

Therefore, the present study asked whether measures of biased interpretation of facial cues can be used to successfully identify violent offenders both compared with the general male population, as well as compared to inmates who sexually abused children.

The hostile attribution bias was investigated using morphed fear-anger expressions and measured both by comparing the percentage of anger responses for ambiguous faces as well as by the characteristics of the emerging psychometric curves, where a lower threshold for recognizing anger would be expected.

The present study also investigated whether male faces can indeed be more diagnostic to identify violent delinquents than are female faces [6]. A task to identify basic expressions of emotion was also carried out to investigate whether violent or child sexual offenders show a more generalized deficit of face recognition. A final question was, how the hostile perception of faces can be related to a direct self-report questionnaire measure of aggression [20, 27], where more aggressive individuals should exhibit generally higher scores. In particular, this questionnaire is designed to differentiate between appetitive and facilitative types of aggression, thereby offering the possibility to investigate whether a hostile attribution bias might be related more to one specific type.

## Methods

### Participants

A total of 62 male participants took part in the study: 30 inmates with violence offences (mean age 42 years, range 21–64), 15 inmates who committed child sexual abuse (mean 42, range 26–57) and 17 non-student controls from the general population (mean 43, range 24–58). These controls were adult males who were enrolled at a local gym; hence they were assumed to have a proclivity to a certain degree of physical competitiveness and were deemed an appropriate control group. Table 1 details the participants’ demographic and clinical characteristics.

**Table 1:** Descriptive statistics for demographic data, PPI-R and SCID-II

Measure	Means (SD)		
	Violent offenders	Child sex offenders	General population
*Demographics*			
Age (years)	42.23 (11.45)	42.07 (8.87)	42.76 (10.33)
Sentence term (months)	93.43 (66.58)	59.07 (24.31)	-
*PPI-R*			
Blame externalization	33.17 (9.61)	37.92 (8.84)	26.65 (7.6)
Rebellious nonconformity	54.31 (16.44)	50.54 (10.69)	53.00 (13.49)
Stress immunity	44.52 (9.65)	44.54 (8.81)	43.00 (6.86)
Social influence	44.83 (8.89)	34.54 (8.90)	45.59 (5.92)
Coldheartedness	32.76 (5.84)	29.62 (3.82)	30.53 (5.92)
Machiavellian egocentricity	34.62 (6.01)	33.85 (5.46)	33.65 (4.76)
Carefree nonplanfulness	27.55 (5.52)	31.15 (6.05)	29.65 (6.66)
Fearlessness	17.83 (5.91)	15.38 (5.06)	18.18 (5.49)
Sum	289.59 (34.98)	277.54 (29.45)	280.24 (27.15)
Dissimulation Score	41.76 (6.46)	45.15 (7.28)	41.59 (7.14)
*SCID-II-Screening*			
Avoidant personality disorder PD	1.50 (1.70)	2.36 (1.98)	0.76 (1.09)
Obsessive-compulsive PD	3.60 (1.63)	4.43 (1.79)	4.29 (2.11)
Negativistic PD	1.57 (1.68)	2.07 (1.90)	1.24 (1.44)
Depressive PD	1.83 (2.07)	3.07 (2.34)	0.88 (1.54)
Paranoid PD	2.57 (2.10)	2.86 (2.57)	1.35 (1.66)
Schizotypal PD	1.23 (0.94)	2.14 (2.38)	1.41 (1.37)
Schizoid PD	1.80 (1.42)	2.64 (1.98)	1.59 (1.00)
Histrionic PD	1.60 (1.81)	0.57 (0.76)	1.41 (1.33)
Narcissistic PD	4.00 (2.94)	3.07 (3.15)	2.18 (1.78)
Borderline PD	3.53 (3.23)	2.21 (2.89)	2.47 (2.69)
Antisocial PD	4.23 (4.19)	2.43 (2.21)	1.82 (2.81)

*PPI-R* Psychopathic Personality Inventory—Revised), *SCID-II* Structured Clinical Interview for DSM Disorders, *PD* personality disorder. For PPI-R and SCID-II, values denote raw sum scores of each scale

All inmates were recruited from a German prison for adult males. To be classified as a violent offender, the person had to commit either some form of assault and battery, extortionate robbery, homicide (attempted or successful) or murder (attempted or successful), but not rape. To be classified as a child sex offender, the inmate had to have committed sexual abuse of a minor, including aggravated sexual abuse.

### Material

#### Face stimuli

**Fig. 1:**
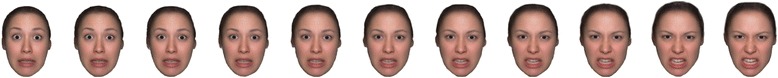
Example stimuli of main experiment. Illustration of a face morphed from the original fearful (*outer left*) to the original angry expression (*outer right*) in nine intermediary steps, resulting in a total of 11 face morphs; due to copyright restrictions, the depicted example is an in-house generated average face [30] which was not used in the present experiment

The face stimuli comprised of 20 identities (10 female, 10 male) as derived from the NimStim [28] and KDEF databases [29]. For each identity, the fear and anger expression were selected and morphed into one another in 10% steps, using GIMP and the GAP toolbox (www.gimp.org). This resulted in 11 morphed expressions per identity (the two original fear and anger faces and nine intermediate morphs), resulting in a total of 220 stimuli. These morphed faces had been used in previous research [30], where they are described in more detail. Figure 1 shows an example.

In addition to this main experiment, there was a test of basic expression recognition (six basic expressions and neutral [31, 32]) with 12 face identities (six male, six female) from the NimStim set.

### Basic emotion recognition task

To test participants’ performance in recognizing full-blown facial expressions of emotion, each experimental session started with a basic emotion recognition task, where all basic expressions and a neutral face were displayed by 12 different actors. Each face was shown for four seconds or as long as it took the participants to make a decision. The participants had to make a 7-way forced-choice decision with the options happy, sad, angry, fearful, disgusted, surprised or neutral.

### Main experiment with morphed faces

Following the basic emotion task, a two-alternatives forced choice identification task was used, in which participants had to decide for each face whether its expression was 'angry' or 'fearful'. Each of the 20 identities was presented in 11 morphing grades. The experiment consisted of two runs with a total of 40 trials per morphing grade. Pictures were shown with no time limit and order of stimuli was randomized, the only constraint being that two subsequent trials never contained the same face identity. Participants had to press the left or right mouse button to indicate whether the target face part showed an angry or fearful expression (button assignment counterbalanced across participants). Experiments were programmed and presented using PsychoPy [33].

#### Questionnaires

After the experiment, participants filled out the *Appetitive and Facilitative Aggression Scale* (AFAS [20]), designed to measure aggressive behaviour. Appetitive aggression refers to violence with the aim to derive pleasure for the suffering of others (example item: *“How often have you provoked others, merely out of enjoyment”*), while facilitative or reactive aggression can be defined as violence to reduce a negative state (example item: *“How often have you destroyed things because you were in pain?”*). There are 15 questions for each scale and participants are instructed to indicate how often in their life they acted or felt in the way described. Each item can be answered on a 5-point scale from 0 (never) to 4 (very often).

Afterwards, participants filled out the *Psychopathic Personality Inventory Revised* (PPI-R [34]) and the *SCID-II* [35]. The PPI-R is a self-assessment questionnaire with 154 items and 9 subscales, such as “coldheartedness”. The SCID-II uses 117 questions to screen for a total of 12 personality disorders, including antisocial personality disorder and was filled out by the inmates as a self-report.

### Data analysis

Data analysis was performed with Python 2.7 (www.python.org) using the toolboxes NumPy, SciPy, Pandas, Matplotlib, Seaborn and the Jupyter Notebook, all as provided with Anaconda 2.4 (Continuum Analytics; docs.continuum.io/anaconda). Analyses of variance (ANOVA) were computed using JASP 0.7.5 [36]. Non-parametric post-hoc tests (Mann–Whitney *U*-Test) were carried out using SciPy [37].

To characterise the participants' performance in psychometric terms, a logistic function (F_logistic_(x;α,β) = 1/[1 + exp(−β(x-α))]) was fitted to the data [38] of each participant. Guess and lapse parameters were added as free parameters, as adapted from the Matlab-based Palamedes Toolbox [39]. After fitting a psychometric function, the threshold parameters, i.e. the point at which the curve is steepest, were subjected to statistical analyses. Here, lower thresholds should indicate an earlier categorization of faces as angry.

## Results

### AFAS questionnaire

**Fig. 2:**
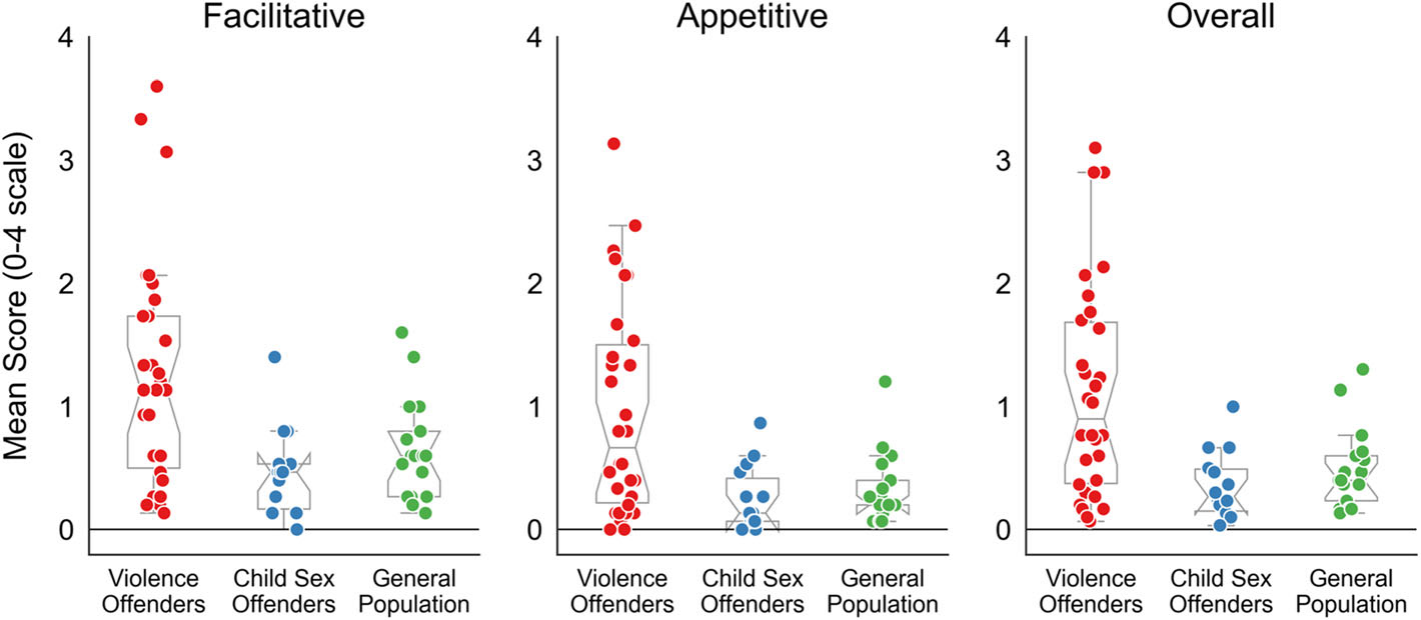
Mean scores of the AFAS questionnaire. Boxplots and raw data from the Appetitive and Facilitative Aggression Scale (AFAS) for all groups across the two subscales as well as the overall mean

On the AFAS subscales of facilitative and appetitive aggression, as well as on the overall mean score, the group of violent offenders scored significantly higher than child sex offenders or the general population, who did not differ from each other (Fig. 2, Table 2). This indicates that, regardless of the types of aggression, the questionnaire measures are elevated only for the violent offenders. Overall, the scores for facilitative aggression were higher than for appetitive aggression (*F*_(1,58)_ = 34.6; *p* < 0.001; *ŋ* ^*2*^ = 0.37), but there was no subscale by group interaction (*F*_(2,58)_ = 0.40; *p* = 0.671; *ŋ* ^*2*^ < 0.01), indicating that differences between groups are equally present on both aggression scales.

**Table 2:** Descriptive and Inferential Statistics for the AFAS questionnaire

	Mean (SD)			Inferential statistics		
Subscale	Violent offenders	Child sex offenders	General population	F_(2,58)_	P	ŋ^2^
Facilitative	1.26 (0.92)^a^	0.47 (0.36)^b^	0.65 (0.41)^b^	7.61	0.001	0.21
Appetitive	0.96 (0.87)^a^	0.25 (0.27)^b^	0.33 (0.29)^b^	8.18	<0.001	0.22
Overall	1.11 (0.88)^a^	0.36 (0.27)^b^	0.49 (0.33)^b^	8.41	<0.001	0.23

Statistical comparisons of the groups using a one-way ANOVA for each subscale of the AFAS questionnaire. In each row, superscript letters that match indicate non-significant difference between groups, while differing superscript letters indicate a group difference significant at *p* < 0.01

For the PPI-R questionnaire there was a group by scale interaction (*F*_(16,472_ = 2.76, *p* < 0.001, *ŋ* ^*2*^ = 0.05), with the violent offenders scoring higher on “social influence” than the child sex offenders and higher than the control population on “blame externalization” (all *p* < 0.05; see Table 4 in the Methods section for descriptive statistics).

For the SCID-II, there was also a group by scale interaction (*F*_(22,649)_ = 2.79, *p* < 0.001, *ŋ* ^*2*^ = 0.07), with the violent offenders scoring higher than the control population for the “antisocial”, “narcissistic” and “paranoid” items (all *p* < 0.05).

### Basic expression recognition task

**Fig. 3:**
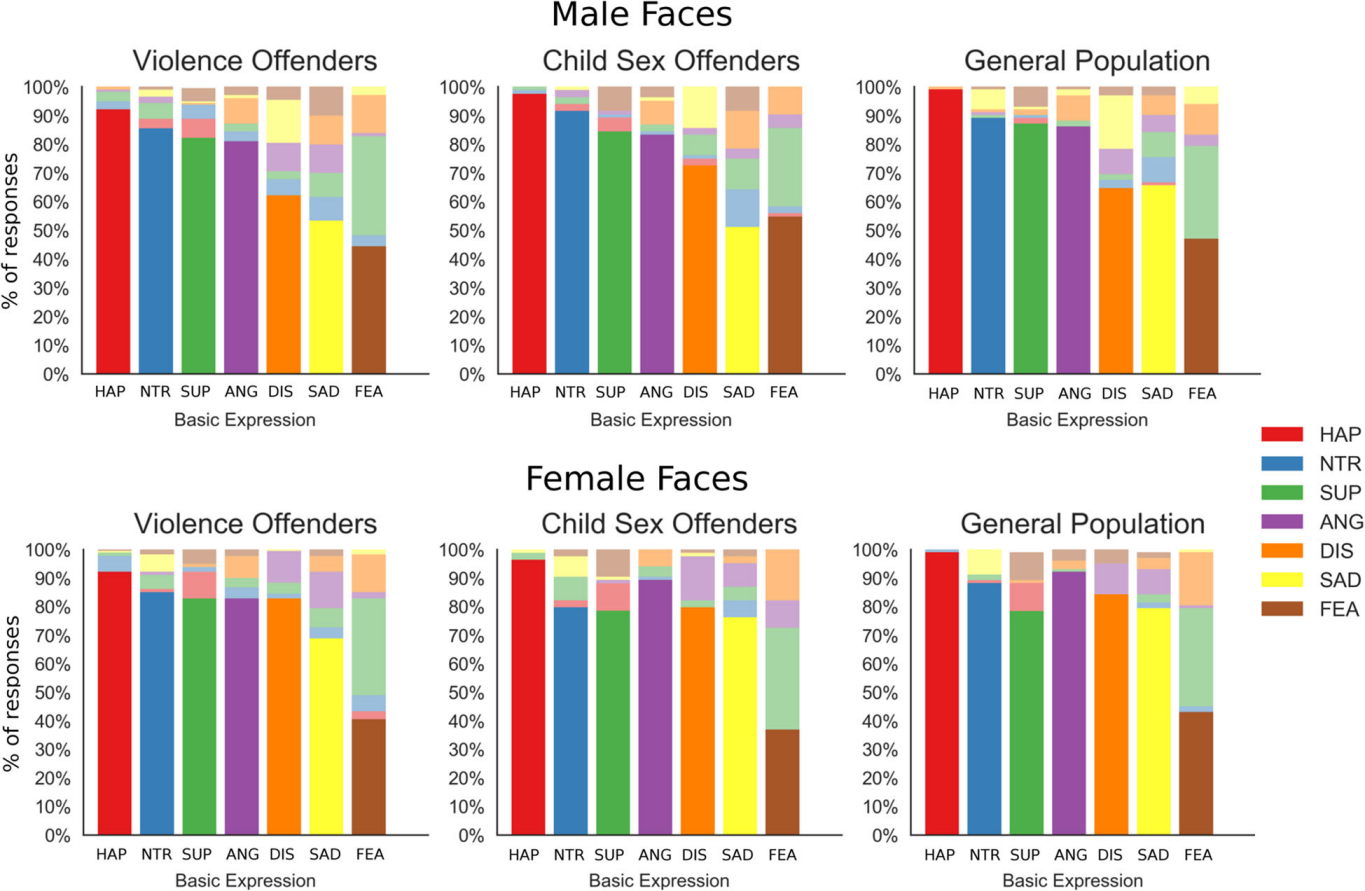
Results of the basic emotion recognition task. Correct responses are plotted in strong colours at the bottom of each bar. Incorrect responses are plotted in muted colours and are at the top of each bar. HAP, happy; NTR, neutral; SUP, surprised; ANG, angry; DIS, disgusted, SAD, sad; FEA, fearful

When the participants had to identify basic expressions in full-blown emotional faces, there was no difference between groups, as indicated by a 3×2×7 ANOVA (with the factors participant group, face gender and emotion expression; Fig. 3, Table 3). While groups did not differ from each other, there was an expected main effect for emotion expression, with highest accuracies for happy faces and lowest accuracies for fearful and sad faces.

**Table 3:** Inferential Statistics for the basic expression recognition task

	Main effects			Interaction effects			
	Gender	Expression	Group	Gender × expression	Gender × group	Expression × group	Gender × expression × group
F	11.88	58.59	0.94	16.70	2.71	0.37	1.47
df	1,58	6,348	2,58	6,348	2,58	12,348	12,348
p	<0.001	<0.001	0.398	<0.001	0.075	0.975	0.134
ŋ^2^	0.16	0.50	0.03	0.22	0.07	<0.01	0.04

Results of a 3 × 2 × 7 ANOVA with the factors participant group, face gender and emotion expression for the basic expression recognition task

There was also a main effect for face gender, in that the expressions of female faces were easier to recognize, across all participant groups (Table 3). This was especially true for disgust and sadness, as indicated by the face gender by expression interaction, as these were significantly easier to recognize in the female models. Overall, the results indicate that no inmate group showed grossly impaired recognition of full-blown facial expressions.

The types of confusions participants made (i.e. mislabel one expression as another) were not analysed statistically, due to their complexity. However, on a descriptive level a common pattern of confusions emerged for all groups, with fear being systematically confused with surprise or disgust with anger (Fig. 3).

### Raw data of face morphing task

In the main experiment with facial expressions morphed from fear to anger, data were first inspected on a single-participant level, which revealed that four violent offenders and one healthy participant performed at chance or exhibited an almost flat response function, indicative of non-compliance (cf. Additional file 1: Code S6). These data were excluded, leaving 26 violent offenders, 16 controls and all 15 child sex offenders for analysis.

**Fig. 4:**
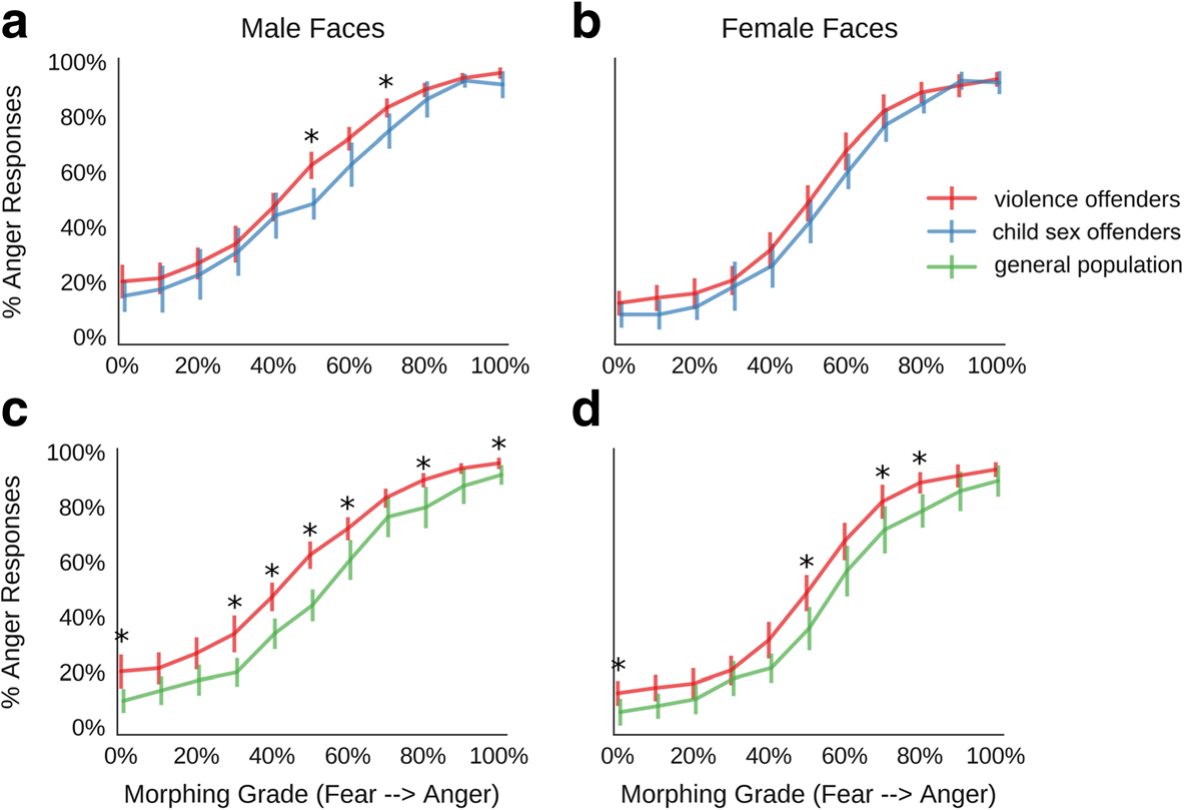
Results for the main experiment with morphed fear-anger expressions. Morphing grade from full-blown fear to full-blown anger on the x-axis; percentage of anger responses on the y-axis; **a**, responses for male faces in violence offenders group compared to child sex offenders; **b**, responses for female faces in violence offenders group compared to child sex offenders; **c**, responses for male faces in violence offenders group compared to control participants; **d**, responses for female faces in violence offenders group compared to control participants; * indicates *p* < 0.05

To analyse the responses in the face morphing task, a 3x2x11 ANOVA (group, face gender and morphing grade), was carried out, which revealed significant main effects for all factors, but no significant interactions (Fig. 4, Table 4). The main effect for morphing grade reflects that anger responses increase as the morphed faces become more angry, as would be expected. The main effect for gender indicates that male faces were overall perceived as more angry, compared to female faces. Finally, the main effect for group reflects that faces were perceived as more angry by the violent offenders, as compared to the other two groups, while child sex offenders and the general population did not differ from each other for any of the 11 morphing grades, as revealed by post-hoc tests.

**Table 4:** ANOVA for the fear-anger morphs

	Main Effects			Interaction effects			
	Gender	Morph	Group	Gender x Morph	Gender x Group	Morph x Group	Gender x Morph x Group
F	29.72	825.15	6.33	1.08	0.47	1.08	1.10
df	1,53	10,530	2,53	20,530	2,53	20,530	20,530
p	<0.001	<0.001	0.003	0.370	0.626	0.370	0.348
ŋ^2^	0.36	0.94	0.19	<0.01	0.01	<0.01	0.03

Results of a 3×2×11 ANOVA with the factors participant group, face gender and morphing grade for the emotion identification task with morphed fear-anger expressions

**Fig. 5:**
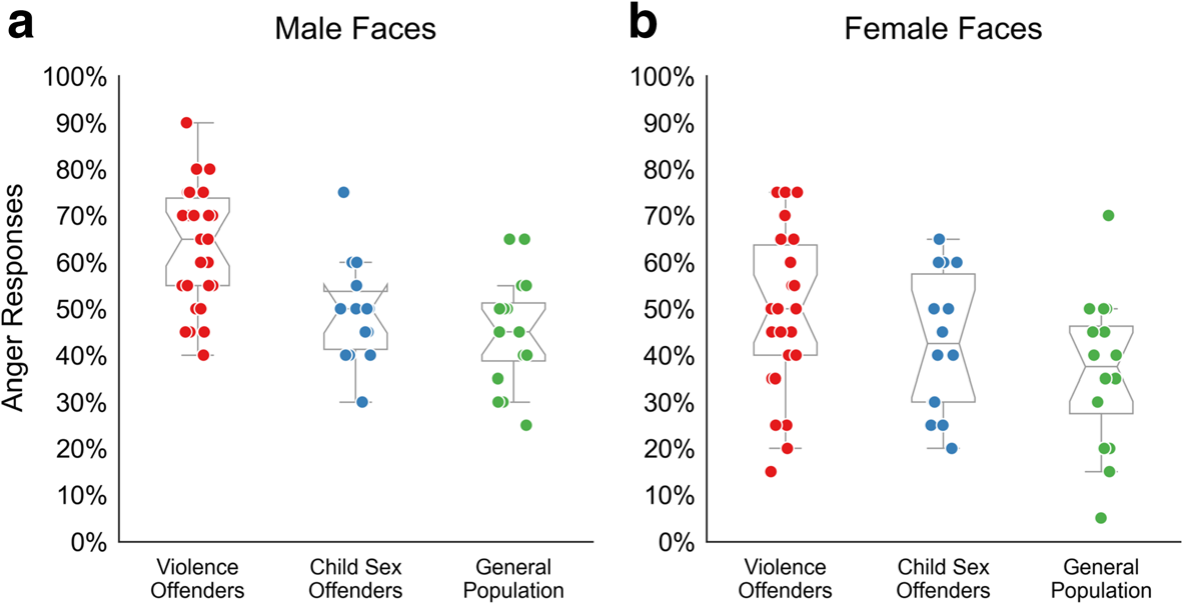
Results for the ambiguous expressions. Results show the percentage of anger responses to the most ambiguous 50% fear – 50% anger faces. *boxplots* are overlaid with raw data of each participant; **a**, responses for male faces; **b**, responses for female faces

As the hostile attribution bias can be expected to be most pronounced for ambiguous faces, the scores for the middle morph (50%fear-50% anger) were subjected to more detailed analysis (Fig. 5). The violent offenders differ significantly from the other two groups when viewing male faces (all *p* < 0.01) and differ from the general population (but not the child sex offenders) when viewing female faces (*p* < 0.05). However, there was no significant interaction of face gender and group membership (*F*_(2,53)_ = 1.65, *p* = 0.203, *ŋ* ^*2*^ = 0.04), indicating that more pronounced group differences for male faces exist only on a descriptive level.

**Fig. 6:**
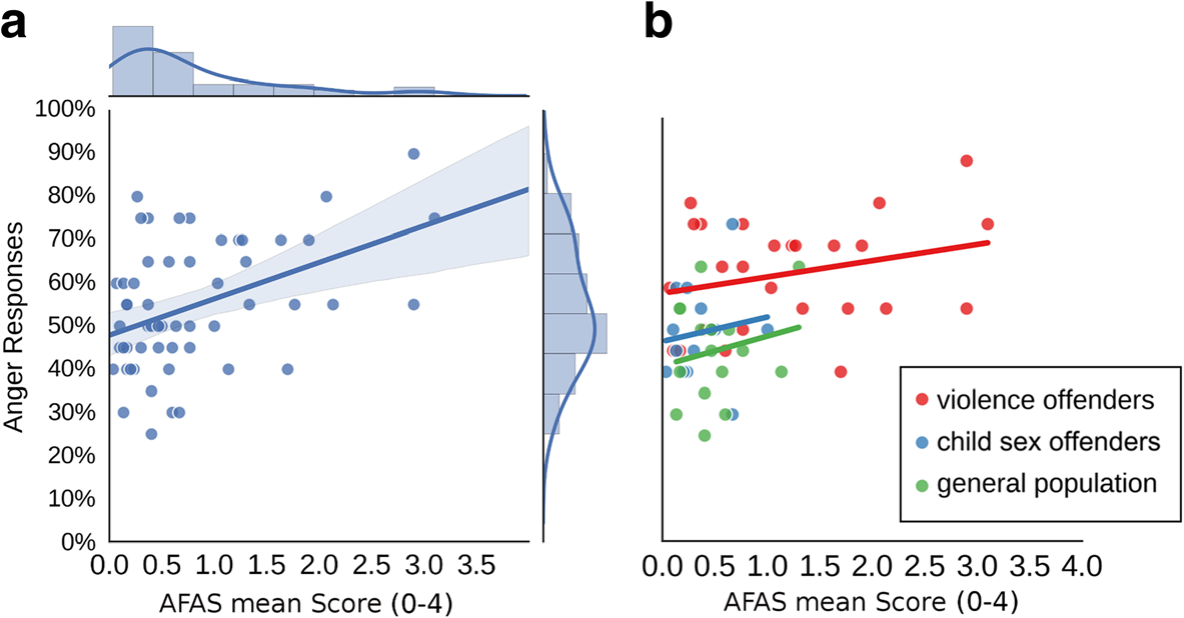
Correlations of face perception and aggressions scores. Scatterplots with the mean AFAS score of each participant on the x-axis and percentage of anger responses for the ambiguous 50–50 face on the y-axis; **a**, plotted for all participants with area around the regression line indicating the 95% confidence interval; **b**, for each group separately, with line length reflecting the range of each sample's data

To investigate the relationship of the rating of the ambiguous middle morph with self-reported aggression scores, a Spearman rank correlation was computed. Figure 6a shows that the higher the overall aggression scores on the AFAS, the more angry will an ambiguous face be rated (*r*_*S*_ = 0.37; *p <* 0.01). Similar correlations emerged when correlating the two AFAS subscales with the face ratings (facilitative aggression: *r*_*S*_ = 0.35; *p <* 0.01; appetitive aggression: *r*_*S*_ = 0.36; *p <* 0.01).

Given the low variability of AFAS scores in the non-violent groups, group differences are only presented descriptively (Fig. 6b).

### Fitting of psychometric functions

**Fig. 7:**
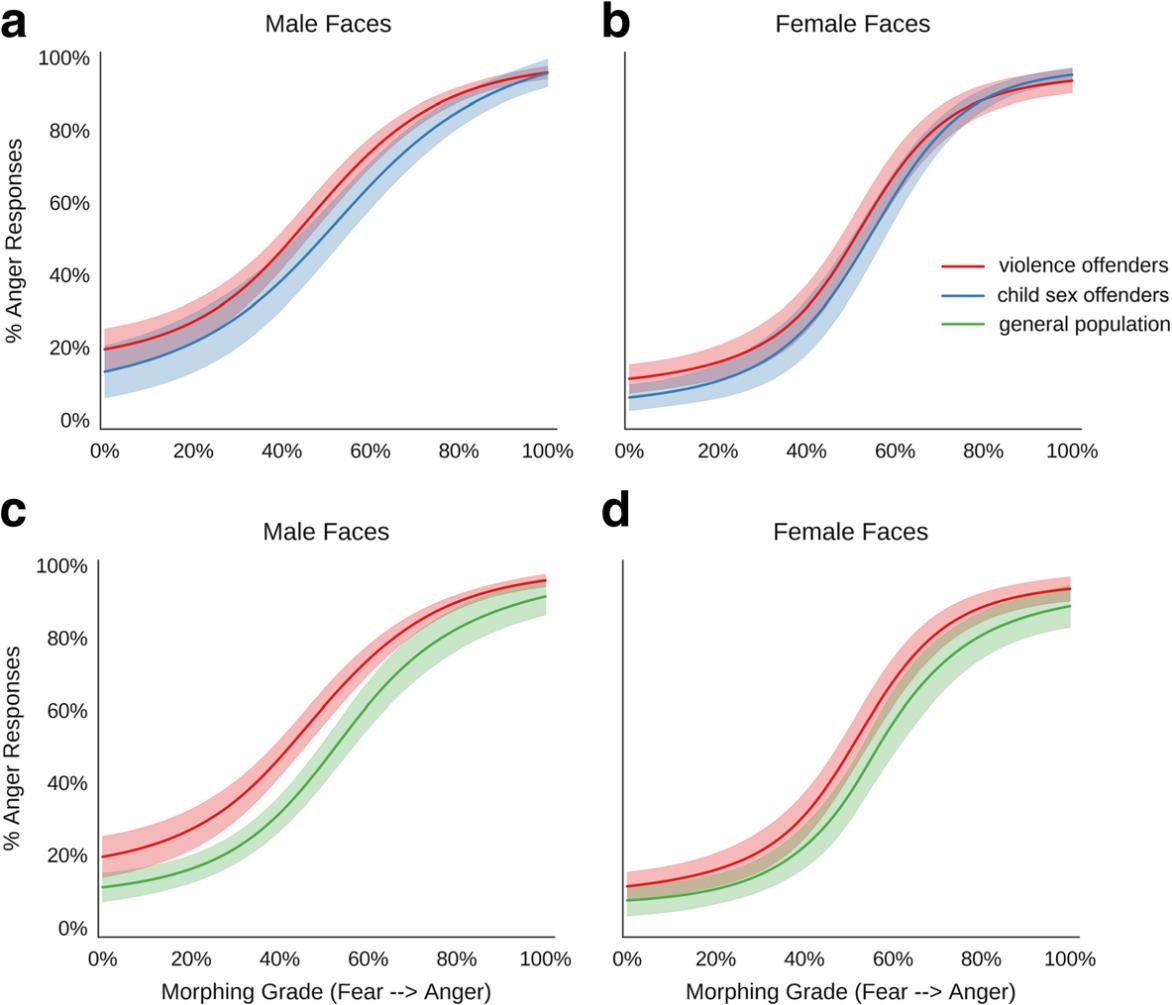
Fitted logistic functions for morphed faces. Logistic functions fitted to each participant’s data were reconstructed in fine-grained 1001 steps on the x-axis and 95% confidence intervals were drawn around each groups mean curve in muted colours; **a**, responses for male faces in violence offenders group compared to child sex offenders; **b**, responses for female faces in violence offenders group compared to child sex offenders; **c**, responses for male faces in violence offenders group compared to control participants; **d**, responses for female faces in violence offenders group compared to control participants

Logistic functions were fitted to the data of each participant and first analysed visually. In addition to the data excluded in the above analyses, one more violent offender and two child sex offenders had to be excluded, as a logistic function could not be fit to their data (e.g. because the threshold would be outside the actual stimulus range, cf. Additional file 2: Code S7). The remaining data were compared between groups using 95% confidence intervals. The results indicate that the psychometric curves only differed between violent offenders and the general population and only for male faces (Fig. 7).

As each psychometric curve has a threshold parameter which tells at which point on the x-axis the slope is steepest (indicating a shift from fear ratings to anger ratings), a low threshold of the curve would indicate that the shift from fear to anger judgements happens earlier, and hence the faces are rated as more angry.

**Fig. 8:**
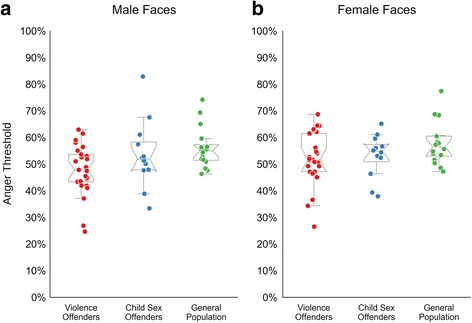
Threshold values for morphed faces. Results for the main experiment with morphed fear-anger expressions, showing the threshold values of the fitted psychometric curves. Boxplots are overlaid with raw data of each participant; **a**, responses for male faces; **b**, responses for female faces

When comparing the threshold values between groups, the violent offenders differed only from the general population and for male faces only (Fig. 8; *p* < 0.05), in that their threshold to perceive anger was significantly lower, in line with the results in Fig. 7.

**Fig. 9:**
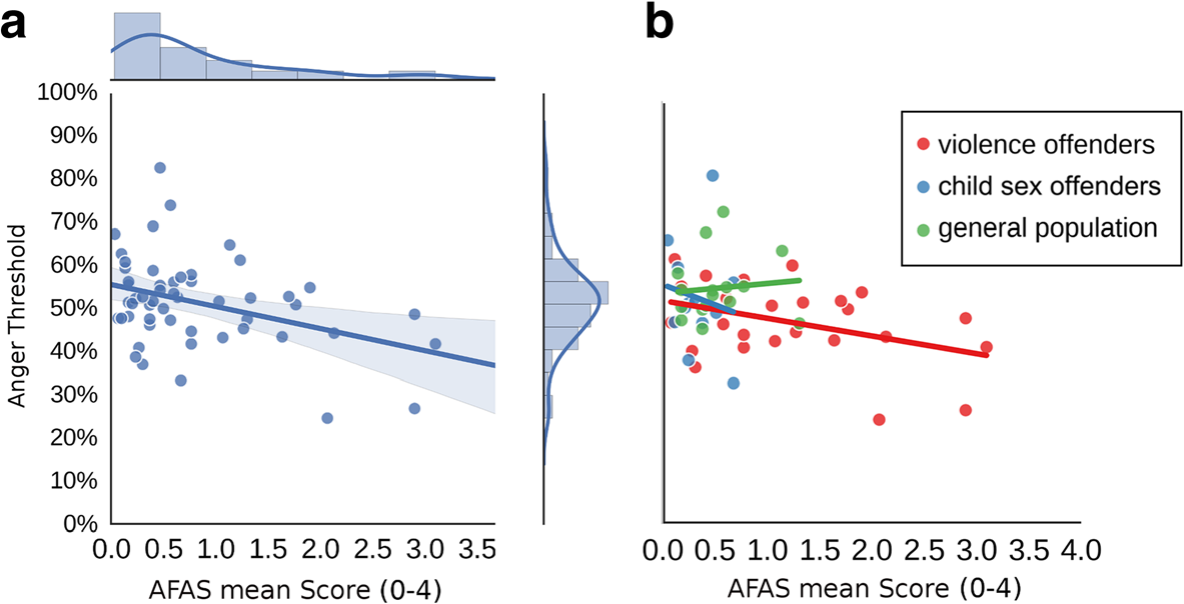
Correlations of threshold values and aggressions scores. Scatterplots with the mean AFAS score of each participant on the x-axis and threshold value of fitted psychometric curves on the y-axis; **a**, for all participants with area around the regression line indicating the 95% confidence interval; **b**, for each group separately, with line length reflecting the range of each sample's data

A correlation of AFAS scores and the threshold values revealed a significant negative correlation, indicating that the higher the self-reported aggression, the lower the threshold to perceive anger (*r*_*S*_ = −0.27,*p* < 0.05; Fig. 9). Similar correlations emerged when correlating the two AFAS subscales with the face ratings (facilitative aggression: *r*_*S*_ = −0.22.; *p =* 0.11; appetitive aggression: *r*_*S*_ = −0.29.; *p <* 0.05). These results are in line with the correlation results with the raw data above, since a lower threshold to recognize a face as angry will translate to more anger responses for the ambiguous morph.

## Discussion

The present study investigated the presence of a hostile attribution bias in violent offenders, as compared to child sex offenders and male controls from the general population. We demonstrated a specific anger bias for morphed fear-anger faces, in absence of a more general impairment in recognizing full-blown basic expressions of emotions. Regarding the morphed faces, differences between violent offenders and both comparison groups were found. These were explained by significant differences for the most ambiguous morph and confirms and extends a similar previous finding in antisocial violent offenders[12]. Analysis of psychometric functions confirmed the differences between violence offenders and the general population, while differences between violence offenders and child sex offenders showed only a trend in this analysis. Also, a tendency of this hostile attribution bias in violence offenders being more pronounced for male than for female faces was found [6, 40] although only as a trend. Finally, a correlation between the hostile attribution bias and self-reported aggression was revealed, in line with research showing that more aggressive individuals will rate ambiguous social signals as more provocative [17, 18]. Overall, the violence offenders showed some evidence of psychopathology, with elevated antisociality scores, while not differing from the control groups on most scales of PPI-R and SCID-II. Hence, this might help to strengthen the link between aggression and hostile attribution in the absence of psychopathology, expanding previous research that showed the hostile attribution bias only for violence offenders with a clinical diagnosis [10].

The results of the present study fit well with the findings by Schönenberg [12], which demonstrated group differences for ambiguous face stimuli, where one end of the emotion spectrum represents anger. There, biased processing was found only for happy-angry and fear-angry morphs but not for happy-fear morphs. This specific role of ambiguous angry faces is also reflected in our results, since no signs of biased perception were found for the basic full-blown emotion expressions. That violent offenders but not child sex offenders show such a specific bias in face recognition might be relevant for diagnostics in a clinical setting, where differentiating between a general deficit in recognizing emotions and a more specific hostile attribution bias might be valuable. Also, using such broad emotion recognition tasks in addition to the fear-anger morphs might help to identify other groups of criminals that have a more global deficit, for example related to psychiatric conditions like psychopathy [41–43] or antisocial personality disorder [9, 12, 18], which could be tapped with such additional tests. Also, given that there is large variance in the violent offenders group regarding their aggression levels and hostile attribution bias, identifying those individuals who exhibit the strongest bias might be important for therapeutic interventions or prognostics. This is especially true since it has been shown that interventions directly aiming to reduce biased perception of ambiguous faces can indeed be successful in reducing aggressive tendencies [13, 26]. At the same time, such a programme might be of little or no use for inmates who present with no hostile attribution bias to begin with. For these inmates, it would be interesting to understand what other factors explain their violent behaviour, allowing to group them into more homogeneous classes with possibly different underlying mechanisms driving their aggressive behaviour and different etiologies explaining why they ended up as inmates.

The face morphing task is also well-suited to identify non-compliance or dissimulation tendencies, as a completely flat psychometric curve is implausible, particularly in the absence of a pronounced overall deficit in expression classification, and an s-shaped function should almost always emerge [22, 30]. A number of violent offenders in the present study were found to perform the task almost at random. In a clinical context such information might be valuable to judge the reliability of other measures (e.g. questionnaires) or to follow-up the diagnostics with additional tests. That dissimulation tendencies in an inmate population should occur is not implausible, as inmates might be particularly concerned that test results, if they become known, will have negative influence on probation or similar decisions. However, one cannot exclude the possibility that the outlier results reflect a real and deep-seated problem with recognizing facial expressions [43], or other more basic cognitive impairments, possibly more frequent in an inmate population or associated with psychiatric conditions.

Therefore, it is important that the present study has compared the violent offenders not only to the general population, but also to inmates charged with sexually abusing children. These comparisons have shown that such finer distinctions between inmate groups are more difficult to draw, as would be expected. Also, both control groups are comparably small and hence future studies should try to replicate the results in larger samples. However, the inclusion of a control group that is also serving prison time, has inflicted serious harm unto others, but scores very low on measures of aggression (i.e. the AFAS), can be considered an important step to better understand the specific traits of violent offenders. That the child sex offenders might also show impaired recognition of facial expressions [44] or low empathy scores [16] has been shown previously. However, whether they would also present with a hostile attribution bias is more of an open question. The results of the present study can only be used to generate hypotheses in this regard. While child sex offenders scored in between violent offenders and general population regarding their hostile attribution bias, differences compared to the general population were too subtle to reach statistical significance. That child sex offenders could also present with a hostile attribution bias is not implausible, as they often had a traumatic childhood which included abuse [45]. This might have shaped their perception of the world as more hostile [11, 46], even though this bias is not directly linked to the nature of their offences. This certainly can also be true for some violent offenders, who might have developed a hostile attribution bias only after being imprisoned and having to deal with a presumably hostile environment. On the other hand, the correlations between self-rated aggression and the biased perception of faces found in the present study suggests that for violent offenders the hostile bias is related more to acting out violence, rather than being its victim. This is well in line with previous work showing similar relationships [15, 47]. Together, these points illustrate that more needs to be learned about the role of the hostile attribution bias for aggressive behavior and the way faces are perceived and judged. Violent offenders exhibited elevated aggression scores on both facilitative and appetitive aggression subscales and the sum score correlated with the hostile attribution bias, but no specific association between either type of aggression and the hostile attribution bias was found. On the basis of reports of “feeling provoked” by others, one might have supposed that elevated levels of facilitative aggression could have been particularly related to the hostile attribution bias. However, this was not borne out. Also, unlike heavily violence-exposed offender populations from crisis regions [20] the present violent offenders showed no specific elevation of appetitive aggression scores.

To better understand the underlying mechanisms of the hostile attribution bias for ambiguously angry faces, future studies could employ eye-tracking or partly masked faces to study whether the bias is due to abnormal inspection strategies of the face. In eye tracking studies with healthy controls, a prominent fixation of the eyes has been found when trying to recognize expressions of emotion [48]. When using masked faces of morphed fear-anger expressions, a strong reliance on the eyes rather then the mouth region has been found as well [30]. Therefore, it would be important to investigate whether a hostile attribution bias is linked to anomalies in the way that violent criminals scan faces or derive information from their different parts. This would be especially interesting for strongly ambiguous stimuli, like the ones used in the present study. Their ambiguity and the necessity to guess gives them a more projective nature, hence they might reveal more about idiosyncratic scanning patterns in violent offenders than full-blown expressions can. Changing such patterns of face inspection in therapeutic interventions could be one possibility to modify the hostile attribution bias, as it has been shown that changing fixation patterns can improve emotion recognition [49] and other studies showed that reducing biased perception can attenuate aggressive behaviour [13].

## Conclusions

Altogether, the present study showed a marked anger bias in violent offenders for ambiguous fear-anger face morphs, in the absence of a more general emotion recognition deficit. Results for these ambiguous faces suggests that the anecdotal self-characterization of violent offenders as individuals who have been provoked or acted merely to defend themselves cannot be taken at face value, but more likely is grounded in a biased perception of social signals. We suggest that this hostile attribution bias might be one mechanism which drives violent behaviour in aggressive delinquents and its better understanding could aid prognostics regarding repeated offences and the development of more specific therapeutic interventions.

## Additional files

Additional file 1: Code S6. Main analysis of morph experiment. (HTML 16207 kb)

Additional file 2: Code S7. Psychophysical analyses of morph experiment (HTML 24228 kb)

Additional file 3: Code S1. Experiment code in PsychoPy. (7Z 2705 kb)

Additional file 4: Data S2. Logfiles with participant data. (7Z 2698 kb)

Additional file 5: Code S3. Analysis of questionnaire data. (HTML 784 kb)

Additional file 6: Code S4. Analysis of basic expression recognition performance. (HTML 489 kb)

Additional file 7: Code S5. Data parsing for morph experiment. (HTML 232 kb)

Additional file 8: Code S8. Executable analysis scripts in.ipynb format. (7Z 37815 kb)
