# Supplementary material for: In your face: the biased judgement of fear-anger expressions in violent offenders
Source: BMC Psychol. 2017 May 12;5:16. doi: 10.1186/s40359-017-0186-z (PMC5429544; doi:10.1186/s40359-017-0186-z)
Supplement: Supplementary file 1 — Main analysis of morph experiment. (HTML 16207 kb) [file 40359_2017_186_MOESM1_ESM.html]

004\_basicPlotting


# In your face: Biased judgement of fear-anger expressions in violence offenders.

# 4. Basic Plotting and Statistics for morphed faces¶

### import libraries¶

In [1]:

```
import numpy as np
import pandas as pd

import os
import fnmatch

import matplotlib.pyplot as plt
import seaborn as sns
%matplotlib inline

from myBasics import *
```

In [2]:

```
bigDf = pd.read_csv('../outputs/genderTable.csv',
                    index_col=[0,1,2])
```

In [3]:

```
bigDf.head()
```

Out[3]:

|  |  |  | 00 | 01 | 02 | 03 | 04 | 05 | 06 | 07 | 08 | 09 | 10 | group |
| --- | --- | --- | --- | --- | --- | --- | --- | --- | --- | --- | --- | --- | --- | --- |
| g | p | fgender |  |  |  |  |  |  |  |  |  |  |  |  |
| A | A001 | F | 0.00 | 0.10 | 0.10 | 0.2 | 0.15 | 0.30 | 0.85 | 0.90 | 0.90 | 1.00 | 1.00 | 2 |
|  |  | M | 0.00 | 0.10 | 0.20 | 0.2 | 0.25 | 0.50 | 0.70 | 0.85 | 0.95 | 1.00 | 1.00 | 2 |
|  | A002 | F | 0.20 | 0.10 | 0.05 | 0.3 | 0.25 | 0.50 | 0.75 | 0.90 | 0.75 | 1.00 | 1.00 | 2 |
|  |  | M | 0.05 | 0.25 | 0.35 | 0.2 | 0.35 | 0.55 | 0.60 | 0.90 | 0.85 | 1.00 | 1.00 | 2 |
|  | A003 | F | 0.00 | 0.05 | 0.15 | 0.1 | 0.30 | 0.40 | 0.55 | 0.65 | 0.65 | 0.65 | 0.85 | 2 |

### One plot for each face gender¶

In [4]:

```
def makeGenderSpaghetti(bigDf,cond,count):
    
    ax = plt.subplot(1,2,count)
    
    # to loop through all cases but have only one legend without redundancies,
    # here we keep track of whether a condition is already labeled
    legTrack = []
    
    # looping  through the 3 groups
    for group in bigDf.index.levels[0]:
        # looping through the participants of each group
        for p in bigDf.ix[group].index:
            if p[0][0] in group:
                
                # color and legend setting for that group
                thisCol = myPal[labelCoding[p[0][0]]]
                thisLeg = myGLabels[p[0][0]]
                
                # get the data from the specified row
                thisList = list(bigDf.ix[group].ix[p[0]].ix[cond])[:-1] # last row is group membership; get rid of that

                # plotting with legend if this is the first instance,
                # otherwise plot without a legend
                if thisLeg not in legTrack:
                    ax.plot(thisList,
                            c=thisCol,
                            alpha=0.5,
                            linewidth=4,
                            label=thisLeg)
                else:
                    ax.plot(thisList,
                            c=thisCol,
                            linewidth=4,
                            alpha=0.5)            
                # keep track which conditions already have a legend
                legTrack.append(thisLeg)
        
    # plot formatting

    ax.set_xlabel('Morphing Grade (Fear --> Anger)')
    ax.set_ylabel('% Anger Responses')

    plt.xticks(np.arange(0,10.1,2), [str(a)+'%' for a in  np.arange(0,101,20)])
    plt.yticks(np.arange(0,1.01,0.2), [str(a)+'%' for a in  np.arange(0,101,20)])

    #plt.ylim(.0,1.); plt.xlim(-0.1,10.2)
    if cond == 'M':
        ax.set_title('Male Faces')
    elif cond == 'F':
        ax.set_title('Female Faces')
        ax.set_ylabel('')
        ax.set_yticks([])
        # one legend for both plots
        plt.legend(loc='best',bbox_to_anchor=[1.1, 1])
```

In [5]:

```
plt.figure(figsize=(12,4))
for i,fgender in enumerate(['M','F']):
    makeGenderSpaghetti(bigDf,fgender,i+1)
    sns.despine()
#plt.savefig('../figures/rawSpaghettiAll.png',dpi=300,bbox_inches="tight")
plt.show()
```

Same thing, but as interactive plot

In [6]:

```
def interactiveSpaghetti(i):
    
    mmDf = bigDf[1::2]
    mDf = mmDf[mmDf.columns[:-1]]
    mY = list( mmDf['group'] )
    
    ffDf = bigDf[0::2]
    fDf = ffDf[ffDf.columns[:-1]]
    fY = list( ffDf['group'])
  
    
    fig = plt.figure(figsize=(12,4))
    
    ### male
    ax = plt.subplot(1,2,1)
    
    selectDf = mDf.ix[i]
    otherDf = np.array( pd.concat([mDf[:i],mDf[i+1:] ] ) )
        
    for entry in range(otherDf.shape[0]):
        try: ax.plot(otherDf[entry],alpha=0.5,color=myPal[mY[entry]])
        except: print "!"
            
    ax.plot(selectDf,linewidth=10,color=myPal[mY[i]])
    
    ### female
    ax = plt.subplot(1,2,2)
    
    selectDf = fDf.ix[i]
    otherDf = np.array( pd.concat([fDf[:i],fDf[i+1:] ] ) )
        
    for entry in range(otherDf.shape[0]):
        try: ax.plot(otherDf[entry],alpha=0.5,color=myPal[fY[entry]])
        except: print "!"
            
    ax.plot(selectDf,linewidth=10,color=myPal[fY[i]])
    sns.despine()
    
    return fig
```

In [7]:

```
# Interactive plots for static html notebooks; using ipywidgets by Jake Vanderplas  
# https://github.com/jakevdp/ipywidgets-static

from ipywidgets_static import StaticInteract, RangeWidget, RadioWidget
```

In [8]:

```
StaticInteract(interactiveSpaghetti,
               i=RangeWidget(0, 60, 1)
              )
```

```
/opt/anaconda2/lib/python2.7/site-packages/matplotlib/pyplot.py:516: RuntimeWarning: More than 20 figures have been opened. Figures created through the pyplot interface (`matplotlib.pyplot.figure`) are retained until explicitly closed and may consume too much memory. (To control this warning, see the rcParam `figure.max_open_warning`).
  max_open_warning, RuntimeWarning)
```

Out[8]:

**i:**

### get rid of outliers¶

Here, some participants are removed whose response functions indicate guessing or non-compliance.  
We also visualise the data of the excluded participants here, to make clear why they had to be excluded.

In [9]:

```
eliminated = ['G011','G013','G016','G025','A004']
```

In [10]:

```
fig = plt.figure(figsize=(16,8))
for e,elim in enumerate(eliminated):
    ax = plt.subplot(2,len(eliminated),e+1)
    ax.plot( bigDf.ix[elim[0]].ix[elim].ix['M'][:-1] )
    ax.set_title(elim+' M')
    ax.set_ylim(0,1)
for e,elim in enumerate(eliminated):
    ax = plt.subplot(2,len(eliminated),len(eliminated)+e+1)
    ax.plot( bigDf.ix[elim[0]].ix[elim].ix['F'][:-1] )
    ax.set_title(elim+' F')
    ax.set_ylim(0,1)
    sns.despine()
plt.tight_layout()
plt.show()
```

### plot cleaned up data¶

In [11]:

```
for elim in eliminated:
    bigDf.drop([elim],level=1, inplace=True)
```

In [12]:

```
plt.figure(figsize=(12,4))
for i,fgender in enumerate(['M','F']):
    makeGenderSpaghetti(bigDf,fgender,i+1)
    sns.despine()
#plt.savefig('../figures/rawSpaghetti.png',dpi=300,bbox_inches="tight")
plt.show()
```

Changing the structure of the DataFrame to easily average over group

In [13]:

```
genderDf = bigDf.unstack(0).unstack(0).stack(1).stack(1)
```

In [14]:

```
genderDf.head()
```

Out[14]:

|  |  |  | 00 | 01 | 02 | 03 | 04 | 05 | 06 | 07 | 08 | 09 | 10 | group |
| --- | --- | --- | --- | --- | --- | --- | --- | --- | --- | --- | --- | --- | --- | --- |
| fgender | g | p |  |  |  |  |  |  |  |  |  |  |  |  |
| F | A | A001 | 0.00 | 0.10 | 0.10 | 0.20 | 0.15 | 0.30 | 0.85 | 0.90 | 0.90 | 1.00 | 1.00 | 2.0 |
|  |  | A002 | 0.20 | 0.10 | 0.05 | 0.30 | 0.25 | 0.50 | 0.75 | 0.90 | 0.75 | 1.00 | 1.00 | 2.0 |
|  |  | A003 | 0.00 | 0.05 | 0.15 | 0.10 | 0.30 | 0.40 | 0.55 | 0.65 | 0.65 | 0.65 | 0.85 | 2.0 |
|  |  | A005 | 0.35 | 0.20 | 0.15 | 0.55 | 0.40 | 0.70 | 0.90 | 1.00 | 1.00 | 1.00 | 1.00 | 2.0 |
|  |  | A006 | 0.00 | 0.20 | 0.05 | 0.25 | 0.20 | 0.35 | 0.65 | 0.55 | 0.85 | 0.85 | 0.95 | 2.0 |

In [15]:

```
# get rid of group column
genderDf = genderDf[genderDf.columns[:-1]]
```

In [16]:

```
genderDf.head()
```

Out[16]:

|  |  |  | 00 | 01 | 02 | 03 | 04 | 05 | 06 | 07 | 08 | 09 | 10 |
| --- | --- | --- | --- | --- | --- | --- | --- | --- | --- | --- | --- | --- | --- |
| fgender | g | p |  |  |  |  |  |  |  |  |  |  |  |
| F | A | A001 | 0.00 | 0.10 | 0.10 | 0.20 | 0.15 | 0.30 | 0.85 | 0.90 | 0.90 | 1.00 | 1.00 |
|  |  | A002 | 0.20 | 0.10 | 0.05 | 0.30 | 0.25 | 0.50 | 0.75 | 0.90 | 0.75 | 1.00 | 1.00 |
|  |  | A003 | 0.00 | 0.05 | 0.15 | 0.10 | 0.30 | 0.40 | 0.55 | 0.65 | 0.65 | 0.65 | 0.85 |
|  |  | A005 | 0.35 | 0.20 | 0.15 | 0.55 | 0.40 | 0.70 | 0.90 | 1.00 | 1.00 | 1.00 | 1.00 |
|  |  | A006 | 0.00 | 0.20 | 0.05 | 0.25 | 0.20 | 0.35 | 0.65 | 0.55 | 0.85 | 0.85 | 0.95 |

In [17]:

```
def makePlot(genderDf,groups):
    plt.figure(figsize=(12,4))

    for count,gender in enumerate( genderDf.index.levels[0][::-1] ):

        ax = plt.subplot(1,2,count+1)

        for j,group in enumerate( groups ):

            thisMean = genderDf.ix[gender].ix[group].mean()
            thisStd = genderDf.ix[gender].ix[group].std(ddof=1)

            thisCol = myPal[labelCoding[group]]
            thisLeg = myGLabels[group]
            
            plt.errorbar( np.linspace(0,10,len(thisMean))+j/15., # apply small shift so CI are better visible
                          thisMean,
                          yerr=thisStd/np.sqrt(len(genderDf.ix[gender].ix[group]) )*1.96,

                          label=thisLeg,
                          color=thisCol,
                          linewidth=3,
                          alpha=0.7
                        )

        # plot formatting

        ax.set_xlabel('Morphing Grade (Fear --> Anger)')
        ax.set_ylabel('% Anger Responses')

        plt.xticks(np.arange(0,10.1,2), [str(a)+'%' for a in  np.arange(0,101,20)])
        plt.yticks(np.arange(0,1.01,0.2), [str(a)+'%' for a in  np.arange(0,101,20)])

        plt.ylim(-0.02,1.02); plt.xlim(-0.1,10.2)
        if gender == 'M':
            ax.set_title('Male Faces')
        elif gender == 'F':
            ax.set_title('Female Faces')
            ax.set_ylabel('')
            ax.set_yticks([])
            # one legend for both plots
            plt.legend(loc='best',bbox_to_anchor=[1, 1])

    #plt.show()
```

In [18]:

```
makePlot(genderDf,['G','A'])
sns.despine()
#plt.savefig('../figures/meanGenderPlotViolenceGeneral.png',dpi=300,bbox_inches="tight")
plt.show()
```

In [19]:

```
makePlot(genderDf,['G','K'])
sns.despine()
#plt.savefig('../figures/meanGenderPlotViolenceMolesters.png',dpi=300,bbox_inches="tight")
plt.show()
```

### Restructure for seaborn plots¶

In [20]:

```
genderStacked = genderDf.unstack(0).stack(0).unstack(2)
```

In [21]:

```
groupCol = []
for entry in genderStacked.index.levels[1]:
    try:
        genderStacked.ix[entry[0]].ix[entry]
        groupCol.append(labelCoding[entry[0]] )
    except:
        print entry # previously eliminated cases are excluded
genderStacked['group'] = groupCol
```

In [22]:

```
genderStacked.head()
```

Out[22]:

|  | fgender | F | | | | | | | | | | ... | M | | | | | | | | | group |
| --- | --- | --- | --- | --- | --- | --- | --- | --- | --- | --- | --- | --- | --- | --- | --- | --- | --- | --- | --- | --- | --- | --- |
|  |  | 00 | 01 | 02 | 03 | 04 | 05 | 06 | 07 | 08 | 09 | ... | 02 | 03 | 04 | 05 | 06 | 07 | 08 | 09 | 10 |  |
| g | p |  |  |  |  |  |  |  |  |  |  |  |  |  |  |  |  |  |  |  |  |  |
| A | A001 | 0.00 | 0.10 | 0.10 | 0.20 | 0.15 | 0.30 | 0.85 | 0.90 | 0.90 | 1.00 | ... | 0.20 | 0.20 | 0.25 | 0.50 | 0.70 | 0.85 | 0.95 | 1.00 | 1.00 | 2 |
|  | A002 | 0.20 | 0.10 | 0.05 | 0.30 | 0.25 | 0.50 | 0.75 | 0.90 | 0.75 | 1.00 | ... | 0.35 | 0.20 | 0.35 | 0.55 | 0.60 | 0.90 | 0.85 | 1.00 | 1.00 | 2 |
|  | A003 | 0.00 | 0.05 | 0.15 | 0.10 | 0.30 | 0.40 | 0.55 | 0.65 | 0.65 | 0.65 | ... | 0.05 | 0.05 | 0.30 | 0.25 | 0.45 | 0.50 | 0.80 | 0.95 | 0.95 | 2 |
|  | A005 | 0.35 | 0.20 | 0.15 | 0.55 | 0.40 | 0.70 | 0.90 | 1.00 | 1.00 | 1.00 | ... | 0.30 | 0.45 | 0.40 | 0.65 | 0.90 | 1.00 | 1.00 | 1.00 | 1.00 | 2 |
|  | A006 | 0.00 | 0.20 | 0.05 | 0.25 | 0.20 | 0.35 | 0.65 | 0.55 | 0.85 | 0.85 | ... | 0.30 | 0.30 | 0.45 | 0.50 | 0.85 | 0.85 | 0.90 | 0.90 | 1.00 | 2 |

5 rows × 23 columns

In [23]:

```
fig = plt.figure(figsize=(12,6))

for i,fGender in enumerate(['M','F']):
    ax = fig.add_subplot('12'+str(i+1))

    sns.boxplot(x='group',y=(fGender,'05'),data=genderStacked,
                    width=0.4,linewidth=1,color='white',whis=True,notch=True,fliersize=0,ax=ax)
    sns.stripplot(x='group',y=(fGender,'05'),data=genderStacked,
                      jitter=True, edgecolor='white',palette=myPal,size=9,linewidth=1,ax=ax)

    if fGender == 'M':
        ax.set_title('Male Faces')
    else :
        ax.set_title('Female Faces')        
        
    ax.set_ylim(0,1)
    ax.set_xticklabels(['Violence\nOffenders','Child\nMolesters','General\nPopulation'],fontsize=15)
    ax.set_xlabel('')
    if i==0:
        ax.set_ylabel('Anger Responses')
    else:
        ax.set_ylabel('')
    sns.despine()
    plt.yticks(np.arange(0,1.01,0.1), [str(a)+'%' for a in  np.arange(0,101,10)])
#plt.suptitle('Threshold of fitted function',fontsize=20, position=(0.51,1.1))
plt.tight_layout()
#plt.savefig('../figures/fifty_fifty.png',dpi=300)
plt.show()
```

### inferential statistics¶

In [24]:

```
from scipy import stats
```

In [25]:

```
def makeMannUTests(df):
    bigDf = pd.DataFrame()
    d = {}
    for comp in [('G','K'),('G','A'),('K','A')]:
        for variable in df.columns:
            group1 = df.ix[comp[0]][variable]
            group2 = df.ix[comp[1]][variable]
            U,p = stats.mannwhitneyu(group1,group2)
            if p<0.05:
                thisSig = '*'
            else:
                thisSig = 'n.s.'
            d[variable] = {'U':round(U,2),'p':round(p,3),'sig':thisSig}
        thisDf = pd.DataFrame(d)
        thisDf = thisDf.reindex_axis(['U','p','sig'], axis=0)
        thisDf.index = [ [comp[0]+' > '+comp[1]]*len(thisDf.index),thisDf.index ]

        bigDf = pd.concat([bigDf,thisDf])
    return bigDf.T
```

In [26]:

```
makeMannUTests(genderDf.unstack(0))[1::2]
```

Out[26]:

|  |  | G > K | | | G > A | | | K > A | | |
| --- | --- | --- | --- | --- | --- | --- | --- | --- | --- | --- |
|  |  | U | p | sig | U | p | sig | U | p | sig |
| 00 | M | 207.5 | 0.473 | n.s. | 293.5 | 0.026 | \* | 144.5 | 0.177 | n.s. |
| 01 | M | 211.5 | 0.408 | n.s. | 280.5 | 0.06 | n.s. | 123 | 0.659 | n.s. |
| 02 | M | 207.5 | 0.475 | n.s. | 283.5 | 0.05 | n.s. | 131.5 | 0.424 | n.s. |
| 03 | M | 195.5 | 0.709 | n.s. | 312 | 0.007 | \* | 156.5 | 0.064 | n.s. |
| 04 | M | 218 | 0.309 | n.s. | 325.5 | 0.002 | \* | 155 | 0.073 | n.s. |
| 05 | M | 286.5 | 0.003 | \* | 352.5 | 0 | \* | 133.5 | 0.377 | n.s. |
| 06 | M | 244 | 0.077 | n.s. | 302 | 0.015 | \* | 122 | 0.691 | n.s. |
| 07 | M | 255.5 | 0.036 | \* | 250.5 | 0.271 | n.s. | 96 | 0.516 | n.s. |
| 08 | M | 196.5 | 0.686 | n.s. | 286.5 | 0.039 | \* | 143 | 0.2 | n.s. |
| 09 | M | 201.5 | 0.572 | n.s. | 252 | 0.241 | n.s. | 128 | 0.507 | n.s. |
| 10 | M | 227 | 0.167 | n.s. | 279 | 0.049 | \* | 118 | 0.812 | n.s. |

In [27]:

```
makeMannUTests(genderDf.unstack(0))[::2]
```

Out[27]:

|  |  | G > K | | | G > A | | | K > A | | |
| --- | --- | --- | --- | --- | --- | --- | --- | --- | --- | --- |
|  |  | U | p | sig | U | p | sig | U | p | sig |
| 00 | F | 218 | 0.306 | n.s. | 290 | 0.03 | \* | 140 | 0.23 | n.s. |
| 01 | F | 228.5 | 0.185 | n.s. | 264 | 0.142 | n.s. | 117 | 0.847 | n.s. |
| 02 | F | 206 | 0.5 | n.s. | 252 | 0.254 | n.s. | 124 | 0.626 | n.s. |
| 03 | F | 201 | 0.597 | n.s. | 238 | 0.438 | n.s. | 110.5 | 0.967 | n.s. |
| 04 | F | 209 | 0.448 | n.s. | 273 | 0.092 | n.s. | 132 | 0.4 | n.s. |
| 05 | F | 222.5 | 0.254 | n.s. | 298 | 0.02 | \* | 136 | 0.326 | n.s. |
| 06 | F | 238 | 0.114 | n.s. | 276.5 | 0.077 | n.s. | 126 | 0.572 | n.s. |
| 07 | F | 234.5 | 0.138 | n.s. | 284 | 0.049 | \* | 130.5 | 0.45 | n.s. |
| 08 | F | 240.5 | 0.095 | n.s. | 313 | 0.006 | \* | 146 | 0.158 | n.s. |
| 09 | F | 178 | 0.918 | n.s. | 254.5 | 0.22 | n.s. | 141.5 | 0.216 | n.s. |
| 10 | F | 202 | 0.56 | n.s. | 240 | 0.391 | n.s. | 118.5 | 0.796 | n.s. |

### save with new header, for ANOVA in JASP¶

In [28]:

```
jaspGender = genderDf.unstack(0)
jaspGender.columns = jaspGender.columns.swaplevel(0, 1)
jaspGender.sortlevel(0, axis=1, inplace=True)
jaspGender.columns = ['fm'[jaspGender.columns.labels[0][x]]+'_'+str(jaspGender.columns.labels[1][x]) for x in range(jaspGender.shape[1]) ]
```

In [29]:

```
jaspGender.head()
```

Out[29]:

|  |  | f\_0 | f\_1 | f\_2 | f\_3 | f\_4 | f\_5 | f\_6 | f\_7 | f\_8 | f\_9 | ... | m\_1 | m\_2 | m\_3 | m\_4 | m\_5 | m\_6 | m\_7 | m\_8 | m\_9 | m\_10 |
| --- | --- | --- | --- | --- | --- | --- | --- | --- | --- | --- | --- | --- | --- | --- | --- | --- | --- | --- | --- | --- | --- | --- |
| g | p |  |  |  |  |  |  |  |  |  |  |  |  |  |  |  |  |  |  |  |  |  |
| A | A001 | 0.00 | 0.10 | 0.10 | 0.20 | 0.15 | 0.30 | 0.85 | 0.90 | 0.90 | 1.00 | ... | 0.10 | 0.20 | 0.20 | 0.25 | 0.50 | 0.70 | 0.85 | 0.95 | 1.00 | 1.00 |
|  | A002 | 0.20 | 0.10 | 0.05 | 0.30 | 0.25 | 0.50 | 0.75 | 0.90 | 0.75 | 1.00 | ... | 0.25 | 0.35 | 0.20 | 0.35 | 0.55 | 0.60 | 0.90 | 0.85 | 1.00 | 1.00 |
|  | A003 | 0.00 | 0.05 | 0.15 | 0.10 | 0.30 | 0.40 | 0.55 | 0.65 | 0.65 | 0.65 | ... | 0.00 | 0.05 | 0.05 | 0.30 | 0.25 | 0.45 | 0.50 | 0.80 | 0.95 | 0.95 |
|  | A005 | 0.35 | 0.20 | 0.15 | 0.55 | 0.40 | 0.70 | 0.90 | 1.00 | 1.00 | 1.00 | ... | 0.20 | 0.30 | 0.45 | 0.40 | 0.65 | 0.90 | 1.00 | 1.00 | 1.00 | 1.00 |
|  | A006 | 0.00 | 0.20 | 0.05 | 0.25 | 0.20 | 0.35 | 0.65 | 0.55 | 0.85 | 0.85 | ... | 0.40 | 0.30 | 0.30 | 0.45 | 0.50 | 0.85 | 0.85 | 0.90 | 0.90 | 1.00 |

5 rows × 22 columns

In [30]:

```
jaspGender.to_csv('../outputs/genderMorphsJASP.csv')
```

## Correlation of 50/50 with AFAS¶

Get the AFAS data

In [31]:

```
afasDf = pd.read_csv('../outputs/meanAFAS.csv',index_col=[0,1])
```

Get rid of the participants that were excluded from the morphing task

In [32]:

```
for elim in eliminated:
    afasDf.drop([elim],level=1, inplace=True)
```

In [33]:

```
afasDf.head()
```

Out[33]:

|  |  | Facilitative | Appetitive | Overall | group |
| --- | --- | --- | --- | --- | --- |
|  | id |  |  |  |  |
| A | A001 | 1.000000 | 0.266667 | 0.633333 | 2 |
|  | A002 | 0.266667 | 0.066667 | 0.166667 | 2 |
|  | A003 | 0.600000 | 0.200000 | 0.400000 | 2 |
|  | A005 | 1.400000 | 1.200000 | 1.300000 | 2 |
|  | A006 | 0.466667 | 0.266667 | 0.366667 | 2 |

Merge together:

In [34]:

```
assert ( afasDf.index == genderStacked.index ).all()
```

In [35]:

```
mergeDf = pd.DataFrame([ afasDf['Overall'], genderStacked['M']['05'], afasDf['group'] ]).T
```

In [36]:

```
sns.jointplot("Overall", "05", data=mergeDf, kind="reg",
              xlim=(0, 4), ylim=(0, 1),stat_func=None,
              scatter_kws={"s": 70,"edgecolor":"white","linewidth":1}
             )

#plt.savefig('../figures/afasFiftyCorrelationAll.png',dpi=300)


sns.lmplot(x="Overall", y="05", data=mergeDf,hue="group",ci=None,truncate=True,palette=myPal,
           scatter_kws={"s": 70,"edgecolor":"white","linewidth":1}
          )
plt.ylim(0,1);plt.xlim(0,4)
#plt.savefig('../figures/afasFiftyCorrelationGroups.png',dpi=300)
```

Out[36]:

```
(0, 4)
```

In [37]:

```
for scale in afasDf.columns[:-1]:
    print "\n",scale
    r, p = stats.spearmanr(afasDf[scale], genderStacked['M']['05'])
    print "Spearman r:",r,"p:",p
    r, p = stats.pearsonr(afasDf[scale], genderStacked['M']['05'])
    print "Pearson r:",r,"p:",p
```

```
Facilitative
Spearman r: 0.348177427724 p: 0.00854869903188
Pearson r: 0.423191595328 p: 0.00115564812856

Appetitive
Spearman r: 0.360013818649 p: 0.00642285626403
Pearson r: 0.423972371242 p: 0.00112899301368

Overall
Spearman r: 0.365846912775 p: 0.00555700546699
Pearson r: 0.435032544467 p: 0.000806331193605
```

In [ ]:

```

```
