# Supplementary material for: In your face: the biased judgement of fear-anger expressions in violent offenders
Source: BMC Psychol. 2017 May 12;5:16. doi: 10.1186/s40359-017-0186-z (PMC5429544; doi:10.1186/s40359-017-0186-z)
Supplement: Supplementary file 2 — Psychophysical analyses of morph experiment (HTML 24228 kb) [file 40359_2017_186_MOESM2_ESM.html]

 
 
  
 005_fittingFunctions 

 
 

 
 


 

 
 

 
 
     
     
     
      
 
   
     

 
 
 
 
 
  In your face: Biased judgement of fear-anger expressions in violence offenders.   5. Fitting Functions ¶  
 
 
 
 
 
 In [1]: 
 
     
     import   numpy   as   np 

 from   scipy   import   stats 
 from   scipy.special   import   erf 
 from   scipy.optimize   import   curve_fit 
 from   scipy.optimize   import   leastsq 

 from   sklearn.linear_model   import   LogisticRegression 
 from   sklearn   import   metrics 

 import   pandas   as   pd 
 pd  .  set_option  (  'max_columns'  ,   100  ) 
 pd  .  set_option  (  'max_rows'  ,   1000  ) 

 import   os 
 import   fnmatch 

 import   matplotlib.pyplot   as   plt 
 %  matplotlib  inline

 import   seaborn   as   sns 

 from   myBasics   import   * 
  

 
 
 

 
 
 
 
 
 
 Defining psychometric functions and plotting some examples ¶  
 
 
 
 
 
 
 
 
 Here, we define a logistic psychometric function and first show some examples how it looks like.  Later, we fit the function to single participant data and extract the parameters (threshold,slope,guess,lapse) that best describe it. These parameters can be used to reconstruct the functions for each participant and they can be used for other computations, like e.g. comparing thresholds between groups. 

 
 
 
 
 
 
 
 
 Dummy x-scale for plotting: 

 
 
 
 
 
 In [2]: 
 
     
     x   =   np  .  linspace  (  0  ,  1  ,  1000  ) 
  

 
 
 

 
 
 
 
 
 
 Logistic Function: ¶   $F_{L}(x;{\alpha},{\beta})=\dfrac{1}{1+exp(- {\beta}(x-{\alpha}))}$
  ; or with guess and lapse parameters: 
$F_{L}(x;{\alpha},{\beta},{\gamma},{\lambda})=\gamma + (1 - \gamma - \lambda)*\dfrac{1}{1+exp(- {\beta}(x-{\alpha}))}$ 

 
 
 
 
 
 In [3]: 
 
     
     # adapted for Python from the Palamedes Toolbox by Nicolaas Prins and Frederick Kingdom (PAL_Logistic.m) 
 # Prins, N & Kingdom, F. A. A. (2009) Palamedes:  Matlab routines for analyzing psychophysical data. 
 # http://www.palamedestoolbox.org 

 def   logisticFunction  (  x  ,  threshold  ,  slope  ,  guess  ,  lapse  ): 
     return   guess   +   (  1.   -   guess   -   lapse  )  *  (  1.  /  (  1.  +  np  .  exp  (  -  slope  *  (  x  -  threshold  )))) 
  

 
 
 

 
 
 
 In [4]: 
 
     
     plt  .  plot  (  x  ,  logisticFunction  (  x  ,  0.5  ,  10  ,  0  ,  0  )); 
 sns  .  despine  () 
  

 
 
 

 
 


   


 
 
 

 

 
 

 
 
 
 
 
 
 Interactive plot to see the effects of wiggling each parameter ¶  
 
 
 
 
 
 In [5]: 
 
     
     # Interactive plots for static html notebooks; using ipywidgets by Jake Vanderplas   
 # https://github.com/jakevdp/ipywidgets-static 

 from   ipywidgets_static   import   StaticInteract  ,   RangeWidget  ,   RadioWidget 
  

 
 
 

 
 
 
 In [6]: 
 
     
     def   iPlot  (  thresh  ,  slope  ,  guess  ,  lapse  ): 
    
     # make x-axis fine-grained 
     x   =   np  .  linspace  (  0  ,   1  ,   1000  ) 
    
     fig  ,   ax   =   plt  .  subplots  (  figsize  =  (  8  ,   6  )) 
     ax  .  plot  (  x  ,   logisticFunction  (  x  ,  thresh  ,  slope  ,  guess  ,  lapse  ), 
             lw  =  5  ,   alpha  =  0.8  ) 

     ax  .  set_xlim  (  0  ,  1  ) 
     ax  .  set_ylim  (  0  ,  1  ) 
    
     thisEquation   =   '$F_{L}=  %s   + (1 -   %s   -   %s  )*  \\  frac{1}{1+exp(- {  %s  }(x-{  %s  }))}$'   %  (  guess  ,  guess  ,  lapse  ,  slope  ,  thresh  ) 
    
     ax  .  text  (  1.1  ,   0.5  ,   thisEquation  ,   fontsize  =  25  ) 
     sns  .  despine  () 
     return   fig 
  

 
 
 

 
 
 
 In [7]: 
 
     
     StaticInteract  (  iPlot  , 
                thresh  =  RangeWidget  (  .  2  ,   0.8  ,   0.1  ), 
                slope  =  RangeWidget  (  10  ,   20  ,   2  ), 
                guess  =  RangeWidget  (  .  0  ,   .  2  ,   0.1  ), 
                lapse  =  RangeWidget  (  .  0  ,   .  2  ,   0.1  ), 
               ) 
  

 
 
 

 
 


   
 
 /opt/anaconda2/lib/python2.7/site-packages/matplotlib/pyplot.py:516: RuntimeWarning: More than 20 figures have been opened. Figures created through the pyplot interface (`matplotlib.pyplot.figure`) are retained until explicitly closed and may consume too much memory. (To control this warning, see the rcParam `figure.max_open_warning`).
  max_open_warning, RuntimeWarning)
 
 
 

  Out[7]: 

 

     
    
     
      
     
       
     
    
     
       
     
    
     
       
     
    
     
       
     
    
     
       
     
    
     
       
     
    
     
       
     
    
     
       
     
    
     
       
     
    
     
       
     
    
     
       
     
    
     
       
     
    
     
       
     
    
     
       
     
    
     
       
     
    
     
       
     
    
     
       
     
    
     
       
     
    
     
       
     
    
     
       
     
    
     
       
     
    
     
       
     
    
     
       
     
    
     
       
     
    
     
       
     
    
     
       
     
    
     
       
     
    
     
       
     
    
     
       
     
    
     
       
     
    
     
       
     
    
     
       
     
    
     
       
     
    
     
       
     
    
     
       
     
    
     
       
     
    
     
       
     
    
     
       
     
    
     
       
     
    
     
       
     
    
     
       
     
    
     
       
     
    
     
       
     
    
     
       
     
    
     
       
     
    
     
       
     
    
     
       
     
    
     
       
     
    
     
       
     
    
     
       
     
    
     
       
     
    
     
       
     
    
     
       
     
    
     
       
     
    
     
       
     
    
     
       
     
    
     
       
     
    
     
       
     
    
     
       
     
    
     
       
     
    
     
       
     
    
     
       
     
    
     
       
     
    
     
       
     
    
     
       
     
    
     
       
     
    
     
       
     
    
     
       
     
    
     
       
     
    
     
       
     
    
     
       
     
    
     
       
     
    
     
       
     
    
     
       
     
    
     
       
     
    
     
       
     
    
     
       
     
    
     
       
     
    
     
       
     
    
     
       
     
    
     
       
     
    
     
       
     
    
     
       
     
    
     
       
     
    
     
       
     
    
     
       
     
    
     
       
     
    
     
       
     
    
     
       
     
    
     
       
     
    
     
       
     
    
     
       
     
    
     
       
     
    
     
       
     
    
     
       
     
    
     
       
     
    
     
       
     
    
     
       
     
    
     
       
     
    
     
       
     
    
     
       
     
    
     
       
     
    
     
       
     
    
     
       
     
    
     
       
     
    
     
       
     
    
     
       
     
    
     
       
     
    
     
       
     
    
     
       
     
    
     
       
     
    
     
       
     
    
     
       
     
    
     
       
     
    
     
       
     
    
     
       
     
    
     
       
     
    
     
       
     
    
     
       
     
    
     
       
     
    
     
       
     
    
     
       
     
    
     
       
     
    
     
       
     
    
     
       
     
    
     
       
     
    
     
       
     
    
     
       
     
    
     
       
     
    
     
       
     
    
     
       
     
    
     
       
     
    
     
       
     
    
     
       
     
    
     
       
     
    
     
       
     
    
     
       
     
    
     
       
     
    
     
       
     
    
     
       
     
    
     
       
     
    
     
       
     
    
     
       
     
    
     
       
     
    
     
       
     
    
     
       
     
    
     
       
     
    
     
       
     
    
     
       
     
    
     
       
     
    
     
       
     
    
     
       
     
    
     
       
     
    
     
       
     
    
     
       
     
    
     
       
     
    
     
       
     
    
     
       
     
    
     
       
     
    
     
       
     
    
     
       
     
    
     
       
     
    
     
       
     
    
     
       
     
    
     
       
     
    
     
       
     
    
     
       
     
    
     
       
     
    
     
       
     
    
     
       
     
    
     
       
     
    
     
       
     
    
     
       
     
    
     
       
     
    
     
       
     
    
     
       
     
    
     
       
     
    
     
       
     
    
     
       
     
    
     
       
     
    
     
       
     
    
     
       
     
    
     
       
     
    
     
       
     
    
     
       
     
    
     
       
     
    
     
       
     
    
     
       
     
    
     
       
     
    
     
       
     
    
     
       
     
    
     
       
     
    
     
       
     
    
     
       
     
    
     
       
     
    
     
       
     
    
     
       
     
    
     
       
     
    
     
       
     
    
     
       
     
    
     
       
     
    
     
       
     
    
     
       
     
    
     
       
     
    
     
       
     
    
     
       
     
    
     
       
     
    
     
       
     
    
     
       
     
    
     
       
     
    
     
       
     
    
     
       
     
    
     
       
     
    
     
       
     
    
     
       
     
    
     
       
     
    
     
       
     
    
     
       
     
    
     
       
     
    
     
       
     
    
     
       
     
    
     
       
     
    
     
       
     
    
     
       
     
    
     
       
     
    
     
       
     
    
     
       
     
    
     
       
     
    
     
       
     
    
     
       
     
    
     
       
     
    
     
       
     
    
     
       
     
    
     
       
     
    
     
       
     
    
     
       
     
    
     
       
     
    
     
       
     
    
     
       
     
    
     
       
     
    
     
       
     
    
     
       
     
    
     
       
     
    
     
       
     
    
     
       
     
    
     
       
     
    
     
       
     
    
     
       
     
    
     
       
     
    
     
       
     
    
     
       
     
    
     
       
     
    
     
       
     
    
     
       
     
    
     
       
     
    
     
       
     
    
     
       
     
    
     
       
     
    
     
       
     
    
     
       
     
    
     
       
     
    
     
       
     
    
     
       
     
    
     
       
     
    
     
       
     
    
     
       
     
    
     
       
     
    
     
       
     
    
     
       
     
    
     
       
     
    
     
       
     
    
     
       
     
    
     
       
     
    
     
       
     
    
     
       
     
    
     
       
     
    
     
       
     
    
     
       
     
    
     
       
     
    
     
       
     
    
     
       
     
    
     
       
     
    
     
       
     
    
     
       
     
    
     
       
     
    
     
       
     
    
     
       
     
    
     
       
     
    
     
       
     
    
     
       
     
    
     
       
     
    
     
       
     
    
     
       
     
    
     
       
     
    
     
       
     
    
     
       
     
    
     
       
     
    
     
       
     
    
     
       
     
    
     
       
     
    
     
       
     
    
     
       
     
    
     
       
     
    
     
       
     
    
     
       
     
    
     
       
     
    
     
       
     
    
     
       
     
    
     
       
     
    
     
       
     
    
     
       
     
    
     
       
     
    
     
       
     
    
     
       
     
    
     
       
     
    
     
       
     
    
     
       
     
    
     
       
     
    
     
       
     
    
     
       
     
    
     
       
     
    
     
       
     
    
     
       
     
    
     
       
     
    
     
       
     
    
     
       
     
    
     
       
     
    
     
       
     
    
     
       
     
    
     
       
     
    
     
       
     
    
     
       
     
    
     
       
     
    
     
       
     
    
     
       
     
    
     
       
     
    
     
       
     
    
     
       
     
    
     
       
     
    
     
       
     
    
     
       
     
    
     
       
     
    
     
       
     
    
     
       
     
    
     
       
     
    
     
       
     
    
     
       
     
    
     
       
     
    
     
       
     
    
     
       
     
    
     
       
     
    
     
       
     
    
     
       
     
    
     
       
     
    
     
       
     
    
     
       
     
    
     
       
     
    
     
       
     
    
     
       
     
    
     
       
     
    
     
       
     
    
     
       
     
    
     
       
     
    
     
       
     
    
     
       
     
    
     
       
     
    
     
       
     
    
     
       
     
    
     
       
     
    
     
       
     
    
     
       
     
    
     
       
     
    
     
       
     
    
     
       
     
    
     
       
     
    
     
       
     
    
     
       
     
    
     
       
     
    
     
       
     
    
     
       
     
    
     
       
     
    
     
       
     
    
     
       
     
    
     
       
     
    
     
       
     
    
     
       
     
    
     
       
     
    
     
       
     
    
     
       
     
    
     
       
     
    
     
       
     
    
     
       
     
    
     
       
     
    
     
       
     
    
     
       
     
    
     
       
     
    
     
       
     
    
     
       
     
    
     
       
     
    
     
       
     
    
     
       
     
    
     
       
     
    
     
       
     
    
     
       
     
    
     
       
     
    
     
       
     
    
     
       
     
    
     
       
     
    
     
       
     
    
     
       
     
    
     
       
     
    
     
       
     
    
     
       
     
    
     
       
     
    
     
       
     
    
     
       
     
    
     
       
     
    
     
       
     
    
     
       
     
    
     
       
     
    
     
       
     
    
     
       
     
    
     
       
     
    
     
       
     
    
     
       
     
    
     
       
     
    
     
       
     
    
     
       
     
    
     
       
     
    
     
       
     
    
     
       
     
    
     
       
     
    
     
       
     
    
     
       
     
    
     
       
     
    
     
       
     
    
     
       
     
    
     
       
     
    
     
       
     
    
     
       
     
    
     
       
     
    
     
       
     
    
     
       
     
    
     
       
     
    
     
       
     
    
     
       
     
    
     
       
     
    
     
       
     
    
     
       
     
    
     
       
     
    
     
       
     
    
     
       
     
    
     
       
     
    
     
       
     
    
     
       
     
    
     
       
     
    
     
       
     
    
     
       
     
    
     
       
     
    
     
       
     
    
     
       
     
    
     
       
     
    
     
       
     
    
     
       
     
    
     
       
     
    
     
       
     
    
     
       
     
    
     
       
     
    
     
       
     
    
     
       
     
    
     
       
     
    
     
       
     
    
     
       
     
    
     
       
     
    
     
       
     
    
     
       
     
    
     
       
     
    
     
       
     
    
     
       
     
    
     
       
     
    
     
       
     
    
     
       
     
    
     
       
     
    
     
       
     
    
     
       
     
    
     
       
     
    
     
       
     
    
     
       
     
    
     
       
     
    
     
       
     
    
     
       
     
    
     
       
     
    
     
       
     
    
     
       
     
    
     
       
     
    
     
       
     
    
     
       
     
    
     
       
     
    
     
       
     
    
     
       
     
    
     
       
     
    
     
       
     
    
     
       
     
    
     
       
     
    
     
       
     
    
     
       
     
    
     
       
     
    
     
       
     
    
     
       
     
    
     
       
     
    
     
       
     
    
     
       
     
    
     
       
     
    
     
       
     
    
     
       
     
    
     
       
     
    
     
       
     
    
     
       
     
    
     
       
     
    
     
       
     
    
     
       
     
    
     
       
     
    
     
       
     
    
     
       
     
    
     
       
     
    
     
       
     
    
     
       
     
    
     
       
     
    
     
       
     
    
     
       
     
    
     
       
     
    
     
       
     
    
     
       
     
    
     
       
     
    
     
       
     
    
     
       
     
    
     
       
     
    
     
       
     
    
     
       
     
    
     
       
     
    
     
       
     
    
     
       
     
    
     
       
     
    
     
       
     
    
     
       
     
    
     
       
     
    
     
       
     
    
     
       
     
    
     
       
     
    
     
       
     
    
     
       
     
    
     
       
     
    
     
       
     
    
     
       
     
    
     
       
     
    
     
       
     
    
     
       
     
    
     
       
     
    
     
       
     
    
     
       
     
    
     
       
     
    
     
       
     
    
     
       
     
    
     
       
     
    
     
       
     
    
     
       
     
    
     
       
     
    
     
       
     
    
     
       
     
    
     
       
     
    
     
       
     
    
     
       
     
    
     
       
     
    
     
       
     
    
     
       
     
    
     
       
     
    
     
       
     
    
     
       
     
    
     
       
     
    
     
       
     
    
     
       
     
    
     
       
     
    
     
       
     
    
     
       
     
    
     
       
     
    
     
       
     
    
     
       
     
    
     
       
     
    
     
       
     
    
     
       
     
    
     
       
     
    
     
       
     
    
     
       
     
    
     
       
     
    
     
       
     
    
     
       
     
    
     
       
     
    
     
       
     
    
     
       
     
    
     
       
     
    
     
       
     
    
     
       
     
    
     
       
     
    
     
       
     
    
     
       
     
    
     
       
     
    
     
       
     
    
     
       
     
    
     
       
     
    
     
       
     
    
     
       
     
    
     
       
     
    
     
       
     
    
     
       
     
    
     
       
     
    
     
       
     
    
     
       
     
    
     
       
     
    
     
       
     
    
     
       
     
    
     
       
     
    
     
       
     
    
     
       
     
    
     
       
     
    
     
       
     
    
     
       
     
    
     
       
     
    
     
       
     
    
     
       
     
    
     
       
     
    
     
       
     
    
     
       
     
    
     
       
     
    
     
       
     
    
     
       
     
    
     
       
     
    
     
       
     
    
     
       
     
    
     
       
     
    
     
       
     
    
     
       
     
    
     
       
     
    
     
       
     
    
     
       
     
    
     
       
     
    
     
       
     
    
     
       
     
    
     
       
     
    
     
       
     
    
     
       
     
    
     
       
     
    
     
       
     
    
     
       
     
    
     
       
     
    
     
       
     
    
     
       
     
    
     
       
     
    
     
       
     
    
     
       
     
    
     
       
     
    
     
       
     
    
     
       
     
    
     
       
     
    
     
       
     
    
     
       
     
    
     
       
     
    
     
       
     
    
     
       
     
    
     
       
     
    
     
       
     
    
     
       
     
    
     
       
     
    
       guess:   
 
 lapse:   
 
 slope:   
 
 thresh:   
     
    
 

 

 
 

 
 
 
 
 
 
 Fitting Functions: ¶  
 
 
 
 
 
 In [8]: 
 
     
     # taken from: Ariel Rokem; 
 # https://github.com/arokem/teach_optimization/blob/master/optimization.ipynb 
 def   err_func  (  params  ,   x  ,   y  ,   func  ): 
         return   y   -   func  (  x  ,   *  params  ) 
  

 
 
 

 
 
 
 In [9]: 
 
     
     def   getFit  (  x  ,  y  ,  func  ,  initalGuesses  ): 
     params  ,   _   =   leastsq  (  err_func  , 
                         initalGuesses  , 
                         args  =  (  x  ,   y  ,   func  ), 
                         maxfev  =  1000000000   # a high number, for safety 
                        ) 
     return   params 
  

 
 
 

 
 
 
 
 
 
 Apply functions to data: ¶  
 
 
 
 
 
 In [10]: 
 
     
     df   =   pd  .  read_csv  (  '../outputs/genderTable.csv'  , 
                  index_col  =  [  0  ,  1  ,  2  ]) 

 df   =   df  [  df  .  columns  [:  -  1  ]] 
  

 
 
 

 
 
 
 In [11]: 
 
     
     df  .  tail  () 
  

 
 
 

 
 


  Out[11]: 

 
 
 
   
     
        
        
        
       00 
       01 
       02 
       03 
       04 
       05 
       06 
       07 
       08 
       09 
       10 
     
     
       g 
       p 
       fgender 
        
        
        
        
        
        
        
        
        
        
        
     
   
   
     
       K 
       K013 
       M 
       0.25 
       0.50 
       0.40 
       0.55 
       0.60 
       0.55 
       0.80 
       0.80 
       1.00 
       0.90 
       1.00 
     
     
       K014 
       F 
       0.00 
       0.00 
       0.05 
       0.00 
       0.00 
       0.25 
       0.45 
       0.70 
       0.85 
       0.95 
       0.95 
     
     
       M 
       0.20 
       0.20 
       0.30 
       0.35 
       0.35 
       0.40 
       0.45 
       0.65 
       0.75 
       0.90 
       0.85 
     
     
       K015 
       F 
       0.10 
       0.15 
       0.20 
       0.20 
       0.30 
       0.45 
       0.70 
       0.75 
       0.90 
       1.00 
       0.95 
     
     
       M 
       0.00 
       0.00 
       0.05 
       0.20 
       0.35 
       0.30 
       0.65 
       0.70 
       0.85 
       1.00 
       1.00 
     
   
 
 
 

 

 
 

 
 
 
 
 
 
 parameters need to be initalized. Here, we set them as following: 
 
 threshold: 0.5 (steepest point at 50%) 
 slope: 5 (not equivalent with the first derivative; instad the meaning will differ for each function) 
 guessing: 0 (assuming no guessing) 
 lapses: 0 (assuming no lapses) 
 

 
 
 
 
 
 In [12]: 
 
     
     initalGuesses   =   0.5  ,  5  ,  0  ,  0 
  

 
 
 

 
 
 
 
 
 
 Example fitting with mean over all control participants: ¶  
 
 
 
 
 
 In [13]: 
 
     
     y   =   np  .  array  (   df  .  ix  [  'A'  ]  .  mean  ()   ) 
 x   =   np  .  linspace  (  0  ,  1  ,  len  (  y  )) 
  

 
 
 

 
 
 
 In [14]: 
 
     
     params   =   getFit  (  x  ,  y  ,  logisticFunction  ,  initalGuesses  ) 
  

 
 
 

 
 
 
 In [15]: 
 
     
     params 
  

 
 
 

 
 


  Out[15]: 


 
 array([ 0.55462598,  7.4084308 ,  0.09465109,  0.08281134]) 
 

 

 
 

 
 
 
 In [16]: 
 
     
     plt  .  ylim  (  0  ,  1  ) 
 plt  .  plot  (   x  ,   y  ,   'o'   ) 
 params   =   getFit  (  x  ,  y  ,  logisticFunction  ,  initalGuesses  ) 
 plt  .  plot  (   x  ,   logisticFunction  (   x  ,   *  params   )   ) 
 plt  .  text  (  1.05  ,   0.5  ,  
          'threshold:   %s  \n  slope:   %s  \n  guess:   %s  \n  lapse:   %s  \n  '  
          %   (  round  (  params  [  0  ],  2  ),  round  (  params  [  1  ],  2  ),  round  (  params  [  2  ],  2  ),  round  (  params  [  3  ],  2  ))  
         ) 
 sns  .  despine  () 
 plt  .  show  () 
  

 
 
 

 
 


   


 
 
 

 

 
 

 
 
 
 
 
 
 Interactive plot, to inspect the fitted function for each participant! ¶  
 
 
 
 
 
 In [17]: 
 
     
     def   makeSinglePlot  (  groupName  ,  participant  ): 

     if   groupName   ==   "violence offenders"  : 
         group   =   'G' 
     elif   groupName   ==   "child molesters"  : 
         group   =   'K'  
     elif   groupName   ==   "general population"  : 
         group   =   'A'  
        
     fig   =   plt  .  figure  (  figsize  =  (  16  ,   6  )) 
    
     myCol   =   myPal  [  labelCoding  [  group  ]] 
    
     for   i  ,  fGender   in   enumerate  ([  'M'  ,  'F'  ]): 
         ax   =   plt  .  subplot  (  1  ,  2  ,  i  +  1  ) 
         try  :  
             #select a participant 
             thisGroup  =   group 
             groupEntries   =   [   n   for   n  ,  x   in   enumerate  (   df  .  index  .  levels  [  1  ]   )   if   x  [  0  ]  ==  thisGroup   ]  
             p   =   df  .  index  .  levels  [  1  ][  groupEntries  [  participant  ]] 

             # get the data and transform to numpy array 
             y   =   np  .  array  (   df  .  ix  [  p  [  0  ]]  .  ix  [  p  ]  .  ix  [  fGender  ]   ) 
             x   =   np  .  linspace  (  0  ,  1  ,  len  (  y  )) 

             # plot the raw data 
             ax  .  plot  (   x  ,   y  , 
                         'o'  ,  markersize  =  12  ,   markeredgecolor  =  'grey'  ,  markerfacecolor  =  myCol   ) 
             # plot the fitted data 
             params   =   getFit  (  x  ,  y  ,  logisticFunction  ,  initalGuesses  ) 
             ax  .  plot  (   x  ,   logisticFunction  (   x  ,   *  params   ), 
                          linewidth  =  8  ,   alpha  =  0.6  ,   color  =  myCol  ) 
             ax  .  text  (  .  6  ,   .  1  ,  
                              'threshold:   %s  \n  slope:   %s  \n  guess:   %s  \n  lapse:   %s  \n  '  
                              %   (  round  (  params  [  0  ],  2  ),  round  (  params  [  1  ],  2  ),  round  (  params  [  2  ],  2  ),  round  (  params  [  3  ],  2  ))  
                             ) 
             ax  .  set_xlim  (  0  ,  1  ) 
             ax  .  set_ylim  (  0  ,  1  ) 

             ax  .  set_title  (  fGender  ) 

             plt  .  suptitle  (  p  ,  fontsize  =  20  ) 
             sns  .  despine  () 
         except  : 

             ax  .  set_xlim  (  0  ,  1  ) 
             ax  .  set_ylim  (  0  ,  1  ) 

             ax  .  set_title  (  'no data'  ) 

             plt  .  suptitle  (  'no data'  ,  fontsize  =  20  ) 
             sns  .  despine  () 
        
     return   fig    
  

 
 
 

 
 
 
 In [18]: 
 
     
     StaticInteract  (  makeSinglePlot  , 
                groupName  =  RadioWidget  ([  'violence offenders'  ,  'child molesters'  ,  'general population'  ]), 
                participant  =  RangeWidget  (  0  ,   29  ,   1  ) 
               ) 
  

 
 
 

 
 


  Out[18]: 

 

     
    
     
      
     
       
     
    
     
       
     
    
     
       
     
    
     
       
     
    
     
       
     
    
     
       
     
    
     
       
     
    
     
       
     
    
     
       
     
    
     
       
     
    
     
       
     
    
     
       
     
    
     
       
     
    
     
       
     
    
     
       
     
    
     
       
     
    
     
       
     
    
     
       
     
    
     
       
     
    
     
       
     
    
     
       
     
    
     
       
     
    
     
       
     
    
     
       
     
    
     
       
     
    
     
       
     
    
     
       
     
    
     
       
     
    
     
       
     
    
     
       
     
    
     
       
     
    
     
       
     
    
     
       
     
    
     
       
     
    
     
       
     
    
     
       
     
    
     
       
     
    
     
       
     
    
     
       
     
    
     
       
     
    
     
       
     
    
     
       
     
    
     
       
     
    
     
       
     
    
     
       
     
    
     
       
     
    
     
       
     
    
     
       
     
    
     
       
     
    
     
       
     
    
     
       
     
    
     
       
     
    
     
       
     
    
     
       
     
    
     
       
     
    
     
       
     
    
     
       
     
    
     
       
     
    
     
       
     
    
     
       
     
    
     
       
     
    
     
       
     
    
     
       
     
    
     
       
     
    
     
       
     
    
     
       
     
    
     
       
     
    
     
       
     
    
     
       
     
    
     
       
     
    
     
       
     
    
     
       
     
    
     
       
     
    
     
       
     
    
     
       
     
    
     
       
     
    
     
       
     
    
     
       
     
    
     
       
     
    
     
       
     
    
     
       
     
    
     
       
     
    
     
       
     
    
     
       
     
    
     
       
     
    
     
       
     
    
     
       
     
    
     
       
     
    
     
       
     
    
     
       
     
    
       groupName:  violence offenders:        child molesters:        general population:  
 
 participant:   
     
    
 

 

 
 

 
 
 
 
 
 
 Get curve parameters ¶  
 
 
 
 
 
 In [19]: 
 
     
     def   makeParamsDict  (  df  ): 
     # dict to write to 
     d   =   {} 
    
     # loop through group 
     for   group   in   df  .  index  .  levels  [  0  ]: 
         # loop through participants of that group 
         for   p   in   df  .  ix  [  group  ]  .  index  .  levels  [  0  ]: 
             if   p  [  0  ]   ==   group  : 
                 # loop through face gender 
                 for   fgender   in   df  .  ix  [  group  ]  .  ix  [  p  ]  .  index  : 
                    
                     # get the data 
                     y   =   np  .  array  (   df  .  ix  [  group  ]  .  ix  [  p  ]  .  ix  [  fgender  ]   ) 
                     x   =   np  .  linspace  (  0  ,  1  ,  len  (  y  )) 

                     # get parameters from fitting 
                     threshold  ,  slope  ,  guess  ,  lapse   =   getFit  (  x  ,  y  ,  logisticFunction  ,  initalGuesses  ) 
                    
                     # either fill dict entry or make the entry and fill then 
                     try  : 
                         d  [  p  ][  fgender  ]   =   {  'threshold'  :  threshold  , 
                                 'slope'  :  slope  , 
                                 'guess'  :  guess  , 
                                 'lapse'  :  lapse 
                                } 
                     except  : 
                         d  [  p  ]   =   {   'F'  :{},   'M'  :{}   } 
                         d  [  p  ][  fgender  ]   =   {  'threshold'  :  threshold  , 
                                          'slope'  :  slope  , 
                                          'guess'  :  guess  , 
                                          'lapse'  :  lapse 
                                          } 
     # output is the dict 
     return   d 
  

 
 
 

 
 
 
 In [20]: 
 
     
     def   makeParamsDf  (  d  ): 
     # empty dict to write to 
     paramsDf   =   pd  .  DataFrame  () 
     # loop through dict 
     for   entry   in   d  : 
         # make a table out of the data of one participant 
         thisDf   =   pd  .  DataFrame  (  d  [  entry  ]) 
         thisDf  .  index   =   [   [  entry  ]  *  len  (  thisDf  ),  thisDf  .  index  ] 
         # reshape, so that results are all in one row 
         thisDf   =   thisDf  .  unstack  () 
         # append to big df 
         paramsDf   =   pd  .  concat  ([  paramsDf  ,  thisDf  ]) 
     # making index nice 
     paramsDf   =   paramsDf  .  sort_index  () 
     paramsDf  .  index   =   [   [  name  [  0  ]   for   name   in   paramsDf  .  index   ],   paramsDf  .  index   ] 
    
     return   paramsDf 
  

 
 
 

 
 
 
 
 
 
 We get a table with all four curve parameters for each participant and each face type (female,male). These data can be compared between groups or used to reconstruct the respective logistic function. 

 
 
 
 
 
 In [21]: 
 
     
     paramsDf   =   makeParamsDf  (  makeParamsDict  (  df  )) 
  

 
 
 

 
 
 
 In [22]: 
 
     
     paramsDf  .  head  () 
  

 
 
 

 
 


  Out[22]: 

 
 
 
   
     
        
        
       F 
       M 
     
     
        
        
       guess 
       lapse 
       slope 
       threshold 
       guess 
       lapse 
       slope 
       threshold 
     
   
   
     
       A 
       A001 
       0.106096 
       0.045968 
       30.023075 
       0.538619 
       0.060214 
       -0.032920 
       8.437888 
       0.527474 
     
     
       A002 
       0.128144 
       0.050533 
       11.404248 
       0.515501 
       0.141569 
       -0.056500 
       6.744358 
       0.556187 
     
     
       A003 
       -0.041611 
       0.187950 
       5.700279 
       0.485627 
       -0.037189 
       -0.202456 
       5.246829 
       0.693392 
     
     
       A004 
       0.403708 
       0.180693 
       17.472763 
       0.907479 
       0.356250 
       0.500000 
       197.740393 
       0.796821 
     
     
       A005 
       0.262098 
       -0.017575 
       11.898831 
       0.471887 
       0.230314 
       -0.026271 
       10.781355 
       0.476161 
     
   
 
 
 

 

 
 

 
 
 
 
 
 
 Spaghetti plots of fitted functions ¶  
 
 
 
 
 
 In [23]: 
 
     
     def   makeGenderSpaghetti  (  paramsDf  ,  cond  ,  count  ): 
    
     # resolution of fitted data 
     x   =   np  .  linspace  (  0  ,  1  ,  1000  ) 
    
     ax   =   plt  .  subplot  (  3  ,  2  ,  count  ) 
    
     # to loop through all cases but have only one legend without redundancies, 
     # here we keep track of whether a condition is already labeled 
     legTrack   =   [] 
    
     # looping  through the 3 groups 
     for   group   in   paramsDf  .  index  .  levels  [  0  ]: 
         # looping through the participants of each group 
         for   p   in   paramsDf  .  ix  [  group  ]  .  index  : 
             if   p  [  0  ]   in   group  : 
                
                 # color and legend setting for that group 
                 thisCol   =   myPal  [  labelCoding  [  p  [  0  ]]] 
                 thisLeg   =   myGLabels  [  p  [  0  ]] 
                
                 # get the data from the specified row 
                 theseParams   =   paramsDf  .  ix  [  group  ]  .  ix  [  p  ]  .  ix  [  cond  ] 
                
                 t   =   theseParams  [  'threshold'  ] 
                 s   =   theseParams  [  'slope'  ] 
                 g   =   theseParams  [  'guess'  ] 
                 l   =   theseParams  [  'lapse'  ] 

                 thisList   =   logisticFunction  (   x  ,   t  ,  s  ,  g  ,  l   ) 

                 # plotting with legend if this is the first instance, 
                 # otherwise plot without a legend 
                 if   thisLeg   not   in   legTrack  : 
                     ax  .  plot  (  thisList  , 
                             c  =  thisCol  , 
                             alpha  =  0.4  , 
                             linewidth  =  3  , 
                             label  =  thisLeg  ) 
                 else  : 
                     ax  .  plot  (  thisList  , 
                             c  =  thisCol  , 
                             linewidth  =  3  , 
                             alpha  =  0.4  )             
                 # keep track which conditions already have a legend 
                 legTrack  .  append  (  thisLeg  ) 
        
     # plot formatting 
     ax  .  set_xlabel  (  'Morphing Grade (Fear --> Anger)'  ) 
     ax  .  set_ylabel  (  '% Anger Responses'  ) 

     plt  .  xticks  (  np  .  arange  (  0  ,  1001  ,  200  ),   [  str  (  a  )  +  '%'   for   a   in    np  .  arange  (  0  ,  101  ,  20  )]) 
     plt  .  yticks  (  np  .  arange  (  0  ,  1.01  ,  0.2  ),   [  str  (  a  )  +  '%'   for   a   in    np  .  arange  (  0  ,  101  ,  20  )]) 

     plt  .  ylim  (  -  0.02  ,  1.02  );   plt  .  xlim  (  -  0.1  ,  1001  ) 
     if   cond   ==   'M'  : 
         ax  .  set_title  (  'Male Faces'  ) 
     elif   cond   ==   'F'  : 
         ax  .  set_title  (  'Female Faces'  ) 
         # one legend for both plots 
         plt  .  legend  (  loc  =  'best'  ,  bbox_to_anchor  =  [  1  ,   1  ]) 
  

 
 
 

 
 
 
 In [24]: 
 
     
     plt  .  figure  (  figsize  =  (  12  ,  12  )) 
 for   i  ,  fgender   in   enumerate  ([  'M'  ,  'F'  ]): 
     makeGenderSpaghetti  (  paramsDf  ,  fgender  ,  i  +  1  ) 
 sns  .  despine  () 
 plt  .  tight_layout  () 
 plt  .  show  () 
  

 
 
 

 
 


   


 
 
 

 

 
 

 
 
 
 
 
 
 eliminate data for which fitting is not possible ¶  
 
 
 
 
 
 In [25]: 
 
     
     eliminated   =   [  'G002'  ,  'G011'  ,  'G013'  ,  'G016'  ,  'G025'  ,  'K003'  ,  'K004'  ,  'A004'  ] 
    
  

 
 
 

 
 
 
 In [26]: 
 
     
     def   showEliminated  (  paramsDf  ,  eliminated  ): 
    
     fig   =   plt  .  figure  (  figsize  =  (  16  ,  6  )) 

     for   e  ,  elim   in   enumerate  (  eliminated  ): 

         thisDf   =   paramsDf  .  ix  [  elim  [  0  ]]  .  ix  [  elim  ]  .  ix  [  'M'  ] 
         guess   =   thisDf  .  ix  [  'guess'  ] 
         lapse   =   thisDf  .  ix  [  'lapse'  ] 
         slope   =   thisDf  .  ix  [  'slope'  ] 
         threshold   =   thisDf  .  ix  [  'threshold'  ] 

         ax   =   plt  .  subplot  (  2  ,  len  (  eliminated  ),  e  +  1  ) 
         ax  .  plot  (   logisticFunction  (  x  ,  threshold  ,  slope  ,  guess  ,  lapse  )   )     

         ax  .  set_title  (  elim  +  ' M'  ) 
         ax  .  set_ylim  (  0  ,  1  ) 

     for   e  ,  elim   in   enumerate  (  eliminated  ): 

         thisDf   =   paramsDf  .  ix  [  elim  [  0  ]]  .  ix  [  elim  ]  .  ix  [  'F'  ] 
         guess   =   thisDf  .  ix  [  'guess'  ] 
         lapse   =   thisDf  .  ix  [  'lapse'  ] 
         slope   =   thisDf  .  ix  [  'slope'  ] 
         threshold   =   thisDf  .  ix  [  'threshold'  ] 

         ax   =   plt  .  subplot  (  2  ,  len  (  eliminated  ),  len  (  eliminated  )  +  e  +  1  ) 
         ax  .  plot  (   logisticFunction  (  x  ,  threshold  ,  slope  ,  guess  ,  lapse  )   ) 

         ax  .  set_title  (  elim  +  ' F'  ) 
         ax  .  set_ylim  (  0  ,  1  ) 
    
         sns  .  despine  () 
        
     plt  .  tight_layout  () 
    
     plt  .  show  () 
  

 
 
 

 
 
 
 In [27]: 
 
     
     showEliminated  (  paramsDf  ,  eliminated  ) 
  

 
 
 

 
 


   


 
 
 

 

 
 

 
 
 
 In [28]: 
 
     
     for   elim   in   eliminated  : 
     paramsDf  .  drop  ([  elim  ],  level  =  1  ,   inplace  =  True  ) 
  

 
 
 

 
 
 
 In [29]: 
 
     
     plt  .  figure  (  figsize  =  (  12  ,  12  )) 
 for   i  ,  fgender   in   enumerate  ([  'M'  ,  'F'  ]): 
     makeGenderSpaghetti  (  paramsDf  ,  fgender  ,  i  +  1  ) 
     sns  .  despine  () 
 plt  .  show  () 
  

 
 
 

 
 


   


 
 
 

 

 
 

 
 
 
 
 
 
 save  cleaned-up as  csv ¶  
 
 
 
 
 
 In [30]: 
 
     
     paramsDf  .  to_csv  (  '../outputs/paramsTable.csv'  ) 
  

 
 
 

 
 
 
 
 
 
 Plott averaged fitted functions ¶  
 
 
 
 
 
 In [31]: 
 
     
     def   makeFittedValuesDf  (  paramsDf  ,  fGender  ): 
    
     funcDf   =   pd  .  DataFrame  () 
     for   i  ,  group   in   enumerate  (   paramsDf  .  index  .  levels  [  0  ]   ): 
         for   p   in   paramsDf  .  index  .  levels  [  1  ]: 
             # we need a try/except here, because some participants might have been  
             # removed from the df (but are still in the non-updated index...) 
             try  : 
                 if   group   in   p  : 

                     # choose the parameters of one participant 
                     thisDf   =   paramsDf  .  ix  [  p  [  0  ]]  .  ix  [  p  ]  .  ix  [  fGender  ] 
                     guess   =   thisDf  .  ix  [  'guess'  ] 
                     lapse   =   thisDf  .  ix  [  'lapse'  ] 
                     slope   =   thisDf  .  ix  [  'slope'  ] 
                     threshold   =   thisDf  .  ix  [  'threshold'  ] 

                     # get the data 
                     yFunc   =   logisticFunction  (  np  .  linspace  (  0  ,  1  ,  1001  ),  threshold  ,  slope  ,  guess  ,  lapse  )  
                     thisDf   =   pd  .  DataFrame  (  yFunc  )  .  T 
                     thisDf  .  index   =   [[  group  ],[  p  ]] 
                     funcDf   =   pd  .  concat  ([  funcDf  ,  thisDf  ]) 

             except  : 
                 print   "...participant   %s   excluded"   %   p 
                
     return   funcDf 
  

 
 
 

 
 
 
 
 
 
 This is a large df, which stores not the raw data of the 11 original steps, but the fitted logistic function's y-values in a 1/1000 resolution. 

 
 
 
 
 
 In [32]: 
 
     
     funcDf   =   makeFittedValuesDf  (  paramsDf  ,  'M'  ) 
  

 
 
 

 
 


   
 
 ...participant A004 excluded
...participant G002 excluded
...participant G011 excluded
...participant G013 excluded
...participant G016 excluded
...participant G025 excluded
...participant K003 excluded
...participant K004 excluded
 
 
 

 
 

 
 
 
 In [33]: 
 
     
     funcDf  .  head  () 
  

 
 
 

 
 


  Out[33]: 

 
 
 
   
     
        
        
       0 
       1 
       2 
       3 
       4 
       5 
       6 
       7 
       8 
       9 
       10 
       11 
       12 
       13 
       14 
       15 
       16 
       17 
       18 
       19 
       20 
       21 
       22 
       23 
       24 
       25 
       26 
       27 
       28 
       29 
       30 
       31 
       32 
       33 
       34 
       35 
       36 
       37 
       38 
       39 
       40 
       41 
       42 
       43 
       44 
       45 
       46 
       47 
       48 
       49 
       ... 
       951 
       952 
       953 
       954 
       955 
       956 
       957 
       958 
       959 
       960 
       961 
       962 
       963 
       964 
       965 
       966 
       967 
       968 
       969 
       970 
       971 
       972 
       973 
       974 
       975 
       976 
       977 
       978 
       979 
       980 
       981 
       982 
       983 
       984 
       985 
       986 
       987 
       988 
       989 
       990 
       991 
       992 
       993 
       994 
       995 
       996 
       997 
       998 
       999 
       1000 
     
   
   
     
       A 
       A001 
       0.071434 
       0.071528 
       0.071623 
       0.071718 
       0.071815 
       0.071912 
       0.072010 
       0.072108 
       0.072208 
       0.072308 
       0.072410 
       0.072512 
       0.072614 
       0.072718 
       0.072823 
       0.072928 
       0.073035 
       0.073142 
       0.073250 
       0.073359 
       0.073469 
       0.073579 
       0.073691 
       0.073804 
       0.073917 
       0.074032 
       0.074147 
       0.074263 
       0.074381 
       0.074499 
       0.074618 
       0.074739 
       0.074860 
       0.074982 
       0.075105 
       0.075229 
       0.075355 
       0.075481 
       0.075608 
       0.075737 
       0.075866 
       0.075997 
       0.076128 
       0.076261 
       0.076394 
       0.076529 
       0.076665 
       0.076802 
       0.076940 
       0.077080 
       ... 
       1.006378 
       1.006595 
       1.006810 
       1.007023 
       1.007235 
       1.007445 
       1.007654 
       1.007861 
       1.008066 
       1.008270 
       1.008471 
       1.008672 
       1.008870 
       1.009068 
       1.009263 
       1.009457 
       1.009650 
       1.009840 
       1.010030 
       1.010218 
       1.010404 
       1.010589 
       1.010772 
       1.010954 
       1.011135 
       1.011313 
       1.011491 
       1.011667 
       1.011842 
       1.012015 
       1.012187 
       1.012358 
       1.012527 
       1.012695 
       1.012861 
       1.013026 
       1.013190 
       1.013352 
       1.013513 
       1.013673 
       1.013832 
       1.013989 
       1.014145 
       1.014300 
       1.014453 
       1.014606 
       1.014757 
       1.014906 
       1.015055 
       1.015202 
     
     
       A002 
       0.162568 
       0.162707 
       0.162847 
       0.162988 
       0.163129 
       0.163272 
       0.163415 
       0.163559 
       0.163704 
       0.163851 
       0.163998 
       0.164146 
       0.164295 
       0.164445 
       0.164596 
       0.164747 
       0.164900 
       0.165054 
       0.165209 
       0.165365 
       0.165522 
       0.165679 
       0.165838 
       0.165998 
       0.166159 
       0.166321 
       0.166484 
       0.166648 
       0.166813 
       0.166979 
       0.167146 
       0.167314 
       0.167484 
       0.167654 
       0.167825 
       0.167998 
       0.168172 
       0.168346 
       0.168522 
       0.168699 
       0.168877 
       0.169057 
       0.169237 
       0.169418 
       0.169601 
       0.169785 
       0.169970 
       0.170156 
       0.170344 
       0.170532 
       ... 
       0.996840 
       0.997215 
       0.997588 
       0.997958 
       0.998327 
       0.998693 
       0.999057 
       0.999419 
       0.999779 
       1.000137 
       1.000493 
       1.000846 
       1.001198 
       1.001547 
       1.001894 
       1.002240 
       1.002583 
       1.002924 
       1.003263 
       1.003600 
       1.003936 
       1.004269 
       1.004600 
       1.004929 
       1.005256 
       1.005582 
       1.005905 
       1.006226 
       1.006546 
       1.006863 
       1.007179 
       1.007493 
       1.007805 
       1.008115 
       1.008423 
       1.008729 
       1.009033 
       1.009336 
       1.009637 
       1.009936 
       1.010233 
       1.010528 
       1.010822 
       1.011114 
       1.011404 
       1.011692 
       1.011979 
       1.012263 
       1.012546 
       1.012828 
     
     
       A003 
       -0.005419 
       -0.005256 
       -0.005093 
       -0.004928 
       -0.004763 
       -0.004597 
       -0.004430 
       -0.004262 
       -0.004094 
       -0.003924 
       -0.003754 
       -0.003583 
       -0.003411 
       -0.003238 
       -0.003064 
       -0.002890 
       -0.002714 
       -0.002538 
       -0.002361 
       -0.002183 
       -0.002004 
       -0.001824 
       -0.001643 
       -0.001462 
       -0.001279 
       -0.001096 
       -0.000911 
       -0.000726 
       -0.000540 
       -0.000353 
       -0.000165 
       0.000024 
       0.000214 
       0.000405 
       0.000596 
       0.000789 
       0.000983 
       0.001177 
       0.001373 
       0.001569 
       0.001767 
       0.001965 
       0.002165 
       0.002365 
       0.002566 
       0.002769 
       0.002972 
       0.003177 
       0.003382 
       0.003588 
       ... 
       0.947579 
       0.948640 
       0.949697 
       0.950751 
       0.951802 
       0.952850 
       0.953894 
       0.954935 
       0.955973 
       0.957007 
       0.958038 
       0.959066 
       0.960091 
       0.961112 
       0.962131 
       0.963145 
       0.964157 
       0.965165 
       0.966170 
       0.967172 
       0.968171 
       0.969166 
       0.970158 
       0.971147 
       0.972133 
       0.973115 
       0.974094 
       0.975070 
       0.976042 
       0.977012 
       0.977978 
       0.978941 
       0.979900 
       0.980857 
       0.981810 
       0.982760 
       0.983707 
       0.984651 
       0.985591 
       0.986528 
       0.987462 
       0.988393 
       0.989321 
       0.990245 
       0.991166 
       0.992084 
       0.992999 
       0.993911 
       0.994819 
       0.995725 
     
     
       A005 
       0.234979 
       0.235029 
       0.235080 
       0.235131 
       0.235183 
       0.235235 
       0.235288 
       0.235342 
       0.235396 
       0.235451 
       0.235506 
       0.235562 
       0.235619 
       0.235676 
       0.235733 
       0.235792 
       0.235851 
       0.235910 
       0.235970 
       0.236031 
       0.236093 
       0.236155 
       0.236218 
       0.236281 
       0.236346 
       0.236411 
       0.236476 
       0.236542 
       0.236609 
       0.236677 
       0.236745 
       0.236815 
       0.236884 
       0.236955 
       0.237027 
       0.237099 
       0.237172 
       0.237245 
       0.237320 
       0.237395 
       0.237471 
       0.237548 
       0.237626 
       0.237704 
       0.237783 
       0.237864 
       0.237945 
       0.238027 
       0.238109 
       0.238193 
       ... 
       1.021540 
       1.021591 
       1.021640 
       1.021690 
       1.021739 
       1.021787 
       1.021835 
       1.021882 
       1.021929 
       1.021975 
       1.022021 
       1.022066 
       1.022111 
       1.022156 
       1.022200 
       1.022243 
       1.022286 
       1.022328 
       1.022371 
       1.022412 
       1.022453 
       1.022494 
       1.022534 
       1.022574 
       1.022614 
       1.022653 
       1.022691 
       1.022730 
       1.022767 
       1.022805 
       1.022842 
       1.022878 
       1.022915 
       1.022951 
       1.022986 
       1.023021 
       1.023056 
       1.023090 
       1.023124 
       1.023158 
       1.023191 
       1.023224 
       1.023256 
       1.023289 
       1.023321 
       1.023352 
       1.023383 
       1.023414 
       1.023445 
       1.023475 
     
     
       A006 
       0.252291 
       0.252324 
       0.252357 
       0.252390 
       0.252424 
       0.252458 
       0.252492 
       0.252527 
       0.252562 
       0.252598 
       0.252634 
       0.252670 
       0.252707 
       0.252744 
       0.252782 
       0.252819 
       0.252858 
       0.252896 
       0.252935 
       0.252975 
       0.253015 
       0.253055 
       0.253096 
       0.253137 
       0.253179 
       0.253221 
       0.253264 
       0.253307 
       0.253350 
       0.253394 
       0.253438 
       0.253483 
       0.253528 
       0.253574 
       0.253621 
       0.253667 
       0.253715 
       0.253762 
       0.253811 
       0.253859 
       0.253909 
       0.253959 
       0.254009 
       0.254060 
       0.254111 
       0.254163 
       0.254216 
       0.254269 
       0.254322 
       0.254377 
       ... 
       0.947198 
       0.947264 
       0.947329 
       0.947394 
       0.947458 
       0.947521 
       0.947583 
       0.947645 
       0.947706 
       0.947767 
       0.947827 
       0.947886 
       0.947944 
       0.948003 
       0.948060 
       0.948117 
       0.948173 
       0.948229 
       0.948284 
       0.948338 
       0.948392 
       0.948446 
       0.948498 
       0.948551 
       0.948602 
       0.948653 
       0.948704 
       0.948754 
       0.948804 
       0.948853 
       0.948901 
       0.948949 
       0.948997 
       0.949044 
       0.949090 
       0.949136 
       0.949182 
       0.949227 
       0.949272 
       0.949316 
       0.949359 
       0.949403 
       0.949445 
       0.949488 
       0.949530 
       0.949571 
       0.949612 
       0.949653 
       0.949693 
       0.949733 
     
   
 
 5 rows × 1001 columns 
 
 

 

 
 

 
 
 
 
 
 
 Do this for both female and male faces, then concatenate. 

 
 
 
 
 
 In [34]: 
 
     
     def   mergeFuncDf  (  paramsDf  ):     
     outFunc   =   pd  .  DataFrame  () 
     for   fGender   in   [  'M'  ,  'F'  ]: 
         thisFunc   =   makeFittedValuesDf  (  paramsDf  ,  fGender  ) 
         thisFunc  .  columns   =   [   [  fGender  ]  *  len  (  thisFunc  .  columns  ),   thisFunc  .  columns   ] 
         outFunc   =   pd  .  concat  ([  outFunc  ,  thisFunc  ],  axis  =  1  ) 
     return   outFunc 
  

 
 
 

 
 
 
 In [35]: 
 
     
     logFuncDf   =   mergeFuncDf  (  paramsDf  ) 
  

 
 
 

 
 


   
 
 ...participant A004 excluded
...participant G002 excluded
...participant G011 excluded
...participant G013 excluded
...participant G016 excluded
...participant G025 excluded
...participant K003 excluded
...participant K004 excluded
...participant A004 excluded
...participant G002 excluded
...participant G011 excluded
...participant G013 excluded
...participant G016 excluded
...participant G025 excluded
...participant K003 excluded
...participant K004 excluded
 
 
 

 
 

 
 
 
 In [36]: 
 
     
     logFuncDf  .  head  () 
  

 
 
 

 
 


  Out[36]: 

 
 
 
   
     
        
        
       M 
       ... 
       F 
     
     
        
        
       0 
       1 
       2 
       3 
       4 
       5 
       6 
       7 
       8 
       9 
       10 
       11 
       12 
       13 
       14 
       15 
       16 
       17 
       18 
       19 
       20 
       21 
       22 
       23 
       24 
       25 
       26 
       27 
       28 
       29 
       30 
       31 
       32 
       33 
       34 
       35 
       36 
       37 
       38 
       39 
       40 
       41 
       42 
       43 
       44 
       45 
       46 
       47 
       48 
       49 
       ... 
       951 
       952 
       953 
       954 
       955 
       956 
       957 
       958 
       959 
       960 
       961 
       962 
       963 
       964 
       965 
       966 
       967 
       968 
       969 
       970 
       971 
       972 
       973 
       974 
       975 
       976 
       977 
       978 
       979 
       980 
       981 
       982 
       983 
       984 
       985 
       986 
       987 
       988 
       989 
       990 
       991 
       992 
       993 
       994 
       995 
       996 
       997 
       998 
       999 
       1000 
     
   
   
     
       A 
       A001 
       0.071434 
       0.071528 
       0.071623 
       0.071718 
       0.071815 
       0.071912 
       0.072010 
       0.072108 
       0.072208 
       0.072308 
       0.072410 
       0.072512 
       0.072614 
       0.072718 
       0.072823 
       0.072928 
       0.073035 
       0.073142 
       0.073250 
       0.073359 
       0.073469 
       0.073579 
       0.073691 
       0.073804 
       0.073917 
       0.074032 
       0.074147 
       0.074263 
       0.074381 
       0.074499 
       0.074618 
       0.074739 
       0.074860 
       0.074982 
       0.075105 
       0.075229 
       0.075355 
       0.075481 
       0.075608 
       0.075737 
       0.075866 
       0.075997 
       0.076128 
       0.076261 
       0.076394 
       0.076529 
       0.076665 
       0.076802 
       0.076940 
       0.077080 
       ... 
       0.954028 
       0.954028 
       0.954028 
       0.954028 
       0.954028 
       0.954029 
       0.954029 
       0.954029 
       0.954029 
       0.954029 
       0.954029 
       0.954029 
       0.954029 
       0.954029 
       0.954029 
       0.954029 
       0.954029 
       0.954030 
       0.954030 
       0.954030 
       0.954030 
       0.954030 
       0.954030 
       0.954030 
       0.954030 
       0.954030 
       0.954030 
       0.954030 
       0.954030 
       0.954030 
       0.954030 
       0.954030 
       0.954030 
       0.954030 
       0.954030 
       0.954030 
       0.954030 
       0.954030 
       0.954031 
       0.954031 
       0.954031 
       0.954031 
       0.954031 
       0.954031 
       0.954031 
       0.954031 
       0.954031 
       0.954031 
       0.954031 
       0.954031 
     
     
       A002 
       0.162568 
       0.162707 
       0.162847 
       0.162988 
       0.163129 
       0.163272 
       0.163415 
       0.163559 
       0.163704 
       0.163851 
       0.163998 
       0.164146 
       0.164295 
       0.164445 
       0.164596 
       0.164747 
       0.164900 
       0.165054 
       0.165209 
       0.165365 
       0.165522 
       0.165679 
       0.165838 
       0.165998 
       0.166159 
       0.166321 
       0.166484 
       0.166648 
       0.166813 
       0.166979 
       0.167146 
       0.167314 
       0.167484 
       0.167654 
       0.167825 
       0.167998 
       0.168172 
       0.168346 
       0.168522 
       0.168699 
       0.168877 
       0.169057 
       0.169237 
       0.169418 
       0.169601 
       0.169785 
       0.169970 
       0.170156 
       0.170344 
       0.170532 
       ... 
       0.943784 
       0.943848 
       0.943911 
       0.943974 
       0.944036 
       0.944097 
       0.944157 
       0.944217 
       0.944276 
       0.944335 
       0.944393 
       0.944450 
       0.944506 
       0.944562 
       0.944617 
       0.944672 
       0.944726 
       0.944780 
       0.944832 
       0.944885 
       0.944936 
       0.944987 
       0.945038 
       0.945088 
       0.945137 
       0.945186 
       0.945234 
       0.945282 
       0.945329 
       0.945376 
       0.945422 
       0.945468 
       0.945513 
       0.945558 
       0.945602 
       0.945645 
       0.945688 
       0.945731 
       0.945773 
       0.945815 
       0.945856 
       0.945897 
       0.945937 
       0.945977 
       0.946017 
       0.946055 
       0.946094 
       0.946132 
       0.946170 
       0.946207 
     
     
       A003 
       -0.005419 
       -0.005256 
       -0.005093 
       -0.004928 
       -0.004763 
       -0.004597 
       -0.004430 
       -0.004262 
       -0.004094 
       -0.003924 
       -0.003754 
       -0.003583 
       -0.003411 
       -0.003238 
       -0.003064 
       -0.002890 
       -0.002714 
       -0.002538 
       -0.002361 
       -0.002183 
       -0.002004 
       -0.001824 
       -0.001643 
       -0.001462 
       -0.001279 
       -0.001096 
       -0.000911 
       -0.000726 
       -0.000540 
       -0.000353 
       -0.000165 
       0.000024 
       0.000214 
       0.000405 
       0.000596 
       0.000789 
       0.000983 
       0.001177 
       0.001373 
       0.001569 
       0.001767 
       0.001965 
       0.002165 
       0.002365 
       0.002566 
       0.002769 
       0.002972 
       0.003177 
       0.003382 
       0.003588 
       ... 
       0.755863 
       0.756162 
       0.756459 
       0.756754 
       0.757048 
       0.757341 
       0.757632 
       0.757922 
       0.758210 
       0.758497 
       0.758782 
       0.759066 
       0.759349 
       0.759630 
       0.759910 
       0.760188 
       0.760465 
       0.760741 
       0.761015 
       0.761288 
       0.761559 
       0.761829 
       0.762098 
       0.762365 
       0.762631 
       0.762896 
       0.763160 
       0.763422 
       0.763682 
       0.763942 
       0.764200 
       0.764457 
       0.764712 
       0.764966 
       0.765219 
       0.765471 
       0.765721 
       0.765971 
       0.766218 
       0.766465 
       0.766710 
       0.766955 
       0.767197 
       0.767439 
       0.767679 
       0.767919 
       0.768157 
       0.768393 
       0.768629 
       0.768863 
     
     
       A005 
       0.234979 
       0.235029 
       0.235080 
       0.235131 
       0.235183 
       0.235235 
       0.235288 
       0.235342 
       0.235396 
       0.235451 
       0.235506 
       0.235562 
       0.235619 
       0.235676 
       0.235733 
       0.235792 
       0.235851 
       0.235910 
       0.235970 
       0.236031 
       0.236093 
       0.236155 
       0.236218 
       0.236281 
       0.236346 
       0.236411 
       0.236476 
       0.236542 
       0.236609 
       0.236677 
       0.236745 
       0.236815 
       0.236884 
       0.236955 
       0.237027 
       0.237099 
       0.237172 
       0.237245 
       0.237320 
       0.237395 
       0.237471 
       0.237548 
       0.237626 
       0.237704 
       0.237783 
       0.237864 
       0.237945 
       0.238027 
       0.238109 
       0.238193 
       ... 
       1.015058 
       1.015088 
       1.015117 
       1.015146 
       1.015175 
       1.015203 
       1.015231 
       1.015259 
       1.015286 
       1.015313 
       1.015340 
       1.015366 
       1.015392 
       1.015418 
       1.015443 
       1.015468 
       1.015493 
       1.015518 
       1.015542 
       1.015566 
       1.015590 
       1.015613 
       1.015636 
       1.015659 
       1.015682 
       1.015704 
       1.015726 
       1.015748 
       1.015770 
       1.015791 
       1.015812 
       1.015833 
       1.015853 
       1.015874 
       1.015894 
       1.015914 
       1.015933 
       1.015953 
       1.015972 
       1.015991 
       1.016009 
       1.016028 
       1.016046 
       1.016064 
       1.016082 
       1.016100 
       1.016117 
       1.016134 
       1.016151 
       1.016168 
     
     
       A006 
       0.252291 
       0.252324 
       0.252357 
       0.252390 
       0.252424 
       0.252458 
       0.252492 
       0.252527 
       0.252562 
       0.252598 
       0.252634 
       0.252670 
       0.252707 
       0.252744 
       0.252782 
       0.252819 
       0.252858 
       0.252896 
       0.252935 
       0.252975 
       0.253015 
       0.253055 
       0.253096 
       0.253137 
       0.253179 
       0.253221 
       0.253264 
       0.253307 
       0.253350 
       0.253394 
       0.253438 
       0.253483 
       0.253528 
       0.253574 
       0.253621 
       0.253667 
       0.253715 
       0.253762 
       0.253811 
       0.253859 
       0.253909 
       0.253959 
       0.254009 
       0.254060 
       0.254111 
       0.254163 
       0.254216 
       0.254269 
       0.254322 
       0.254377 
       ... 
       0.913736 
       0.914347 
       0.914954 
       0.915560 
       0.916162 
       0.916762 
       0.917360 
       0.917954 
       0.918546 
       0.919136 
       0.919722 
       0.920307 
       0.920888 
       0.921467 
       0.922044 
       0.922617 
       0.923189 
       0.923757 
       0.924324 
       0.924887 
       0.925448 
       0.926007 
       0.926563 
       0.927116 
       0.927667 
       0.928216 
       0.928762 
       0.929305 
       0.929846 
       0.930385 
       0.930921 
       0.931454 
       0.931986 
       0.932514 
       0.933041 
       0.933565 
       0.934086 
       0.934605 
       0.935122 
       0.935636 
       0.936148 
       0.936658 
       0.937165 
       0.937670 
       0.938172 
       0.938672 
       0.939170 
       0.939665 
       0.940159 
       0.940649 
     
   
 
 5 rows × 2002 columns 
 
 

 

 
 

 
 
 
 
 
 
 Plot fitted functions ¶  
 
 
 
 
 
 In [37]: 
 
     
     def   plotSmoothFuncs  (  funcDf  ,  myGroups  ,  numSteps  =  1001  ,  myMax  =  1.02  ,  myPal  =  myPal  ,  myLabels  =  labelCoding  ): 
     fig   =   plt  .  figure  (  figsize  =  (  16  ,  6  )) 

    
     for   j  ,  fGender   in   enumerate  ([  'M'  ,  'F'  ]): 
         ax   =   plt  .  subplot  (  1  ,  2  ,  j  +  1  ) 
         # now loop through all groups, including the violence offenders (G) 
         for   i  ,  group   in   enumerate  (  myGroups  ): 

             # get mean value of that group 
             meanFunc   =   funcDf  [  fGender  ]  .  ix  [  group  ]  .  mean  () 
             # get standard deviation of that group 
             stdFunc   =   funcDf  [  fGender  ]  .  ix  [  group  ]  .  std  (  ddof  =  1  ) 
             # get group size 
             n   =   len  (  funcDf  [  fGender  ]  .  ix  [  group  ]) 
             # compute 95% confidence interval 
             ciFunc   =   (  stdFunc  /  np  .  sqrt  (   n   )   )  *  1.96 
             # compute the lower and upper bounds of the CI 
             upperBound   =   meanFunc   +   ciFunc 
             lowerBound   =   meanFunc   -   ciFunc 
             # plot the mean value 
             ax  .  plot  (  np  .  linspace  (  0  ,  1  ,  numSteps  ),   meanFunc  , 
                     color  =  myPal  [  myLabels  [  group  ]], 
                     label  =  myGLabels  [  group  ]) 
            
             # plot the error/CI bounds 
             ax  .  fill_between  (   np  .  linspace  (  0  ,  1  ,  numSteps  )   ,   lowerBound  ,   upperBound  ,  
                              color  =  myPal  [  myLabels  [  group  ]],   alpha  =  0.3  ) 
            
             ax  .  set_xlabel  (  'Morphing Grade (Fear --> Anger)'  ) 
             ax  .  set_ylabel  (  '% Anger Responses'  ) 

             plt  .  xticks  (  np  .  arange  (  0  ,  1.01  ,  0.2  ),   [  str  (  a  )  +  '%'   for   a   in    np  .  arange  (  0  ,  101  ,  20  )]) 
             plt  .  yticks  (  np  .  arange  (  0  ,  1.01  ,  0.2  ),   [  str  (  a  )  +  '%'   for   a   in    np  .  arange  (  0  ,  101  ,  20  )]) 

             plt  .  ylim  (  -  0.02  ,  myMax  );   plt  .  xlim  (  -  0.01  ,  1.02  ) 
             if   fGender   ==   'M'  : 
                 ax  .  set_title  (  'Male Faces'  ) 
             elif   fGender   ==   'F'  : 
                 ax  .  set_title  (  'Female Faces'  ) 
                 ax  .  set_ylabel  (  ''  ) 
                 ax  .  set_yticks  ([]) 
                
     plt  .  legend  (  loc  =  'best'  ) 
     sns  .  despine  () 
  

 
 
 

 
 
 
 In [38]: 
 
     
     plotSmoothFuncs  (  logFuncDf  ,[  'G'  ,  'K'  ]); 
 #plt.savefig('../figures/fittedFuncViolenceChildM.png',dpi=300) 
  

 
 
 

 
 


   


 
 
 

 

 
 

 
 
 
 In [39]: 
 
     
     plotSmoothFuncs  (  logFuncDf  ,[  'G'  ,  'A'  ]) 
 #plt.savefig('../figures/fittedFuncViolenceGenPop.png',dpi=300) 
  

 
 
 

 
 


   


 
 
 

 

 
 

 
 
 
 
 
 
 plot curve parameters ¶  
 
 
 
 
 
 
 
 
 Get a new column indicating group membership 

 
 
 
 
 
 In [40]: 
 
     
     groupCol   =   [] 
 for   entry   in   paramsDf  .  index  .  levels  [  1  ]: 
     try  : 
         paramsDf  .  ix  [  entry  [  0  ]]  .  ix  [  entry  ] 
         groupCol  .  append  (  labelCoding  [  entry  [  0  ]]   ) 
     except  : 
         print   entry   # previously eliminated cases are excluded 
  

 
 
 

 
 


   
 
 A004
G002
G011
G013
G016
G025
K003
K004
 
 
 

 
 

 
 
 
 In [41]: 
 
     
     paramsDf  [  'group'  ]   =   groupCol 
  

 
 
 

 
 
 
 
 
 
 Table now has new column: 

 
 
 
 
 
 In [42]: 
 
     
     paramsDf  .  head  () 
  

 
 
 

 
 


  Out[42]: 

 
 
 
   
     
        
        
       F 
       M 
       group 
     
     
        
        
       guess 
       lapse 
       slope 
       threshold 
       guess 
       lapse 
       slope 
       threshold 
        
     
   
   
     
       A 
       A001 
       0.106096 
       0.045968 
       30.023075 
       0.538619 
       0.060214 
       -0.032920 
       8.437888 
       0.527474 
       2 
     
     
       A002 
       0.128144 
       0.050533 
       11.404248 
       0.515501 
       0.141569 
       -0.056500 
       6.744358 
       0.556187 
       2 
     
     
       A003 
       -0.041611 
       0.187950 
       5.700279 
       0.485627 
       -0.037189 
       -0.202456 
       5.246829 
       0.693392 
       2 
     
     
       A005 
       0.262098 
       -0.017575 
       11.898831 
       0.471887 
       0.230314 
       -0.026271 
       10.781355 
       0.476161 
       2 
     
     
       A006 
       0.035116 
       -0.034113 
       5.780194 
       0.607114 
       0.249233 
       0.046546 
       10.672619 
       0.509249 
       2 
     
   
 
 
 

 

 
 

 
 
 
 
 
 
 Plot the curve parameters ¶  
 
 
 
 
 
 
 
 
 Only the threshold value: 

 
 
 
 
 
 In [43]: 
 
     
     fig   =   plt  .  figure  (  figsize  =  (  12  ,  8  )) 

 for   i  ,  fGender   in   enumerate  ([  'M'  ,  'F'  ]): 
     ax   =   fig  .  add_subplot  (  '12'  +  str  (  i  +  1  )) 

     sns  .  boxplot  (  x  =  'group'  ,  y  =  (  fGender  ,  'threshold'  ),  data  =  paramsDf  , 
                 width  =  0.4  ,  linewidth  =  1  ,  color  =  'white'  ,  whis  =  True  ,  notch  =  True  ,  fliersize  =  0  ,  ax  =  ax  ) 
     sns  .  stripplot  (  x  =  'group'  ,  y  =  (  fGender  ,  'threshold'  ),  data  =  paramsDf  , 
                   jitter  =  True  ,   edgecolor  =  'white'  ,  palette  =  myPal  ,  size  =  9  ,  linewidth  =  1  ,  ax  =  ax  ) 
    
     if   fGender   ==   'M'  : 
         ax  .  set_title  (  'Male Faces'  ) 
     else   : 
         ax  .  set_title  (  'Female Faces'  )         
        
     ax  .  set_ylim  (  0  ,  1  ) 
     ax  .  set_xticklabels  ([  'Violence  \n  Offenders'  ,  'Child  \n  Molesters'  ,  'General  \n  Population'  ],  fontsize  =  15  ) 
     ax  .  set_xlabel  (  ''  ) 
     if   i  ==  0  : 
         ax  .  set_ylabel  (  'Anger Threshold'  ) 
     else  : 
         ax  .  set_ylabel  (  ''  ) 
     sns  .  despine  () 
     plt  .  yticks  (  np  .  arange  (  0  ,  1.01  ,  0.1  ),   [  str  (  a  )  +  '%'   for   a   in    np  .  arange  (  0  ,  101  ,  10  )]) 

 #plt.suptitle('Threshold of fitted function',fontsize=20, position=(0.51,1.1)) 
 plt  .  tight_layout  () 
 #plt.savefig('../figures/thresholdPic.png',dpi=300) 
 plt  .  show  () 
  

 
 
 

 
 


   


 
 
 

 

 
 

 
 
 
 
 
 
 All curve parameters: 

 
 
 
 
 
 In [44]: 
 
     
     fig   =   plt  .  figure  (  figsize  =  (  20  ,  12  )) 
 i   =   1 
 for   fGender   in   [  'M'  ,  'F'  ]: 
     for   param   in   [  'threshold'  ,  'slope'  ,  'guess'  ,  'lapse'  ]: 

         ax   =   fig  .  add_subplot  (  int  (  '24'  +  str  (  i  ))) 

         sns  .  boxplot  (  x  =  'group'  ,  y  =  (  fGender  ,  param  ),  data  =  paramsDf  , 
                         width  =  0.4  ,  linewidth  =  1  ,  color  =  'white'  ,  whis  =  True  ,  notch  =  True  ,  fliersize  =  0  ,  ax  =  ax  ) 
         sns  .  stripplot  (  x  =  'group'  ,  y  =  (  fGender  ,  param  ),  data  =  paramsDf  , 
                           jitter  =  True  ,   edgecolor  =  'white'  ,  palette  =  myPal  ,  ax  =  ax  ) 
         sns  .  despine  () 
        
         i  +=   1 
 plt  .  tight_layout  () 
 plt  .  show  () 
  

 
 
 

 
 


   


 
 
 

 

 
 

 
 
 
 
 
 
 inferential statistics ¶  
 
 
 
 
 
 In [45]: 
 
     
     def   makeMannUTests  (  df  ): 
     bigDf   =   pd  .  DataFrame  () 
     d   =   {} 
     for   comp   in   [(  'G'  ,  'K'  ),(  'G'  ,  'A'  ),(  'K'  ,  'A'  )]: 
         for   variable   in   df  .  columns  : 
             group1   =   df  .  ix  [  comp  [  0  ]][  variable  ] 
             group2   =   df  .  ix  [  comp  [  1  ]][  variable  ] 
             U  ,  p   =   stats  .  mannwhitneyu  (  group1  ,  group2  ) 
             if   p  <  0.05  : 
                 thisSig   =   '*' 
             else  : 
                 thisSig   =   'n.s.' 
             d  [  variable  ]   =   {  'U'  :  round  (  U  ,  2  ),  'p'  :  round  (  p  ,  3  ),  'sig'  :  thisSig  } 
         thisDf   =   pd  .  DataFrame  (  d  ) 
         thisDf   =   thisDf  .  reindex_axis  ([  'U'  ,  'p'  ,  'sig'  ],   axis  =  0  ) 
         thisDf  .  index   =   [   [  comp  [  0  ]  +  ' > '  +  comp  [  1  ]]  *  len  (  thisDf  .  index  ),  thisDf  .  index   ] 

         bigDf   =   pd  .  concat  ([  bigDf  ,  thisDf  ]) 
     return   bigDf  .  T 
  

 
 
 

 
 
 
 In [46]: 
 
     
     makeMannUTests  (  paramsDf  [  'M'  ]) 
  

 
 
 

 
 


  Out[46]: 

 
 
 
   
     
        
       G > K 
       G > A 
       K > A 
     
     
        
       U 
       p 
       sig 
       U 
       p 
       sig 
       U 
       p 
       sig 
     
   
   
     
       guess 
       197 
       0.131 
       n.s. 
       252 
       0.169 
       n.s. 
       89 
       0.763 
       n.s. 
     
     
       lapse 
       193 
       0.168 
       n.s. 
       218 
       0.64 
       n.s. 
       78 
       0.417 
       n.s. 
     
     
       slope 
       206 
       0.072 
       n.s. 
       207 
       0.862 
       n.s. 
       64 
       0.144 
       n.s. 
     
     
       threshold 
       114 
       0.249 
       n.s. 
       104 
       0.011 
       * 
       79 
       0.444 
       n.s. 
     
   
 
 
 

 

 
 

 
 
 
 In [47]: 
 
     
     makeMannUTests  (  paramsDf  [  'F'  ]) 
  

 
 
 

 
 


  Out[47]: 

 
 
 
   
     
        
       G > K 
       G > A 
       K > A 
     
     
        
       U 
       p 
       sig 
       U 
       p 
       sig 
       U 
       p 
       sig 
     
   
   
     
       guess 
       193 
       0.168 
       n.s. 
       244 
       0.245 
       n.s. 
       90 
       0.798 
       n.s. 
     
     
       lapse 
       157 
       0.833 
       n.s. 
       167 
       0.385 
       n.s. 
       76 
       0.365 
       n.s. 
     
     
       slope 
       179 
       0.355 
       n.s. 
       229 
       0.446 
       n.s. 
       98 
       0.944 
       n.s. 
     
     
       threshold 
       133 
       0.592 
       n.s. 
       137 
       0.095 
       n.s. 
       74 
       0.318 
       n.s. 
     
   
 
 
 

 

 
 

 
 
 
 
 
 
 Correlate with AFAS ¶  
 
 
 
 
 
 
 
 
 get the AFAS data, clean up and merge: 

 
 
 
 
 
 In [48]: 
 
     
     afasDf   =   pd  .  read_csv  (  '../outputs/meanAFAS.csv'  ,  index_col  =  [  0  ,  1  ]) 
  

 
 
 

 
 
 
 In [49]: 
 
     
     for   elim   in   eliminated  : 
     afasDf  .  drop  ([  elim  ],  level  =  1  ,   inplace  =  True  ) 
  

 
 
 

 
 
 
 In [50]: 
 
     
     assert   (   afasDf  .  index   ==   paramsDf  .  index   )  .  all  () 
  

 
 
 

 
 
 
 In [51]: 
 
     
     mergeDf   =   pd  .  DataFrame  ([   afasDf  [  'Overall'  ],   paramsDf  [  'M'  ][  'threshold'  ],   afasDf  [  'group'  ]   ])  .  T 
  

 
 
 

 
 
 
 In [52]: 
 
     
     sns  .  jointplot  (  "Overall"  ,   "threshold"  ,   data  =  mergeDf  ,   kind  =  "reg"  , 
               xlim  =  (  0  ,   4  ),   ylim  =  (  0  ,   1  ),  stat_func  =  None  , 
               scatter_kws  =  {  "s"  :   70  ,  "edgecolor"  :  "white"  ,  "linewidth"  :  1  } 
              ) 

 #plt.savefig('../figures/afasThreshCorrelationAll.png',dpi=300) 


 sns  .  lmplot  (  x  =  "Overall"  ,   y  =  "threshold"  ,   data  =  mergeDf  ,  hue  =  "group"  ,  ci  =  None  ,  truncate  =  True  ,  palette  =  myPal  , 
            scatter_kws  =  {  "s"  :   70  ,  "edgecolor"  :  "white"  ,  "linewidth"  :  1  } 
           ) 
 plt  .  ylim  (  0  ,  1  );  plt  .  xlim  (  0  ,  4  ) 
 #plt.savefig('../figures/afasThreshCorrelationGroups.png',dpi=300) 
  

 
 
 

 
 


  Out[52]: 


 
 (0, 4) 
 

 

   


 
 
 

 

   


 
 
 

 

 
 

 
 
 
 In [53]: 
 
     
     r  ,   p   =   stats  .  spearmanr  (  afasDf  [  'Overall'  ],   paramsDf  [  'M'  ][  'threshold'  ]) 
 print   "r:"  ,  r  ,  "p:"  ,  p 
  

 
 
 

 
 


   
 
 r: -0.271747990354 p: 0.049022490739
 
 
 

 
 

 
 
 
 In [54]: 
 
     
     r  ,   p   =   stats  .  pearsonr  (  afasDf  [  'Overall'  ],   paramsDf  [  'M'  ][  'threshold'  ]) 
 print   "r:"  ,  r  ,  "p:"  ,  p 
  

 
 
 

 
 


   
 
 r: -0.378826197719 p: 0.00515447315191
 
 
 

 
 

 
 
 
 In [55]: 
 
     
     for   scale   in   afasDf  .  columns  [:  -  1  ]: 
     print   "  \n  "  ,  scale 
     r  ,   p   =   stats  .  spearmanr  (  afasDf  [  scale  ],   paramsDf  [  'M'  ][  'threshold'  ]) 
     print   "Spearman r:"  ,  r  ,  "p:"  ,  p 
     r  ,   p   =   stats  .  pearsonr  (  afasDf  [  scale  ],   paramsDf  [  'M'  ][  'threshold'  ]) 
     print   "Pearson r:"  ,  r  ,  "p:"  ,  p 
  

 
 
 

 
 


   
 
 
Facilitative
Spearman r: -0.221010906663 p: 0.11175521498
Pearson r: -0.355250849129 p: 0.00904502534878

Appetitive
Spearman r: -0.295655082027 p: 0.0316041082614
Pearson r: -0.384477110938 p: 0.0044769476685

Overall
Spearman r: -0.271747990354 p: 0.049022490739
Pearson r: -0.378826197719 p: 0.00515447315191
 
 
 

 
 

 
 
 
 
 
 
 Export threshold parameters to JASP ¶  
 
 
 
 
 
 In [56]: 
 
     
     jaspParams   =   paramsDf  .  copy  (  deep  =  True  ) 
 jaspParams  .  columns   =   jaspParams  .  columns  .  reorder_levels  ([  1  ,  0  ]) 
 jaspParams   =   jaspParams  [  'threshold'  ]  .  copy  (  deep  =  True  ) 

 group   =   [] 
 for   x   in   jaspParams  .  index  .  levels  [  1  ]: 
     try  : 
         jaspParams  .  ix  [  x  [  0  ]]  .  ix  [  x  ] 
         group  .  append  (  labelCoding  [  x  [  0  ]]) 
     except  : 
         print   x 

 jaspParams  [  'group'  ]   =   group 
  

 
 
 

 
 


   
 
 A004
G002
G011
G013
G016
G025
K003
K004
 
 
 

 
 

 
 
 
 In [57]: 
 
     
     jaspParams  .  head  () 
  

 
 
 

 
 


  Out[57]: 

 
 
 
   
     
        
        
       F 
       M 
       group 
     
   
   
     
       A 
       A001 
       0.538619 
       0.527474 
       2 
     
     
       A002 
       0.515501 
       0.556187 
       2 
     
     
       A003 
       0.485627 
       0.693392 
       2 
     
     
       A005 
       0.471887 
       0.476161 
       2 
     
     
       A006 
       0.607114 
       0.509249 
       2 
     
   
 
 
 

 

 
 

 
 
 
 In [58]: 
 
     
     jaspParams  .  to_csv  (  '../outputs/thresholdJASP.csv'  ) 
  

 
 
 

 
     
   
 
 
