# Supplementary material for: In your face: the biased judgement of fear-anger expressions in violent offenders
Source: BMC Psychol. 2017 May 12;5:16. doi: 10.1186/s40359-017-0186-z (PMC5429544; doi:10.1186/s40359-017-0186-z)
Supplement: Supplementary file 5 — Analysis of questionnaire data. (HTML 784 kb) [file 40359_2017_186_MOESM5_ESM.html]

001\_questionnaires


# In your face: Biased judgement of fear-anger expressions in violence offenders.

# 1. Questionnaire Data¶

This is an anlaysis of the AFAS, PPI-R and SCID-II scores of the participants.

### import modules¶

In [1]:

```
import numpy as np
from scipy import stats
import pandas as pd

import os
import fnmatch

import seaborn as sns
import matplotlib.pyplot as plt
%matplotlib inline

from myBasics import *
```

# AFAS¶

### Overview of the questions the AFAS contains¶

In [2]:

```
for entry in open('../experiment/quest/app/forms.py','r'):
    if 'u"' in entry:
        print entry[entry.find('u"')+2:-3]
```

```
1. Traten Sie aus Frust einen Gegenstand oder warfen ihn umher?
2. Fanden Sie Sportarten aufregender, in denen Blut floss?
3. Waren Sie so gereizt, dass Sie sich an anderen Menschen abreagierten?
4. Wollten Sie jemanden noch mehr einschüchtern, wenn Sie merkten, dass diese Person Angst vor Ihnen hatte?
5. Schlugen Sie aus Ärger so fest auf den Tisch, dass Ihnen die Faust danach weh tat?
6. Wollten Sie möglichst vor Ort sein, wenn Sie von einem grausamen Ereignis hörten?
7. Provozierten Sie andere, einfach weil es Ihnen Spaß machte?
8. Wenn Sie wütend auf jemanden waren, malten Sie sich schlimme Dinge aus, die der betreffenden Person zustoßen könnten?
9. Schauten Sie einer Schlägerei zu, auch wenn Sie sich selbst dabei in Gefahr gebracht haben?
10. Rutschte Ihnen die Hand aus, weil jemand Sie provozierte?
11. Machte es Ihnen Spaß, sich zu prügeln?
12. Wenn Sie sich bedroht fühlten, ging es Ihnen besser, wenn Sie sich vorstellten der betreffenden Person zu schaden?
13. Gewöhnten Sie sich an die Bilder von Gewalt, sodass Sie immer grausamere Bilder anschauen mussten, um genauso fasziniert zu sein?
14. Wenn Sie frustriert waren, suchten Sie körperliche Auseinandersetzungen, um den Frust abzubauen?
15. Fanden Sie Gewaltfilme aufregend, in denen die Opfer richtig leiden mussten?
16. Machte es Ihnen Spaß, mit anderen Ihre Kräfte zu messen, auch wenn Sie den anderen weh taten?
17. Fühlten Sie sich für einen Moment besser, wenn Sie Ihren Frust an anderen ausließen?
18. Stachelten Sie andere dazu an, jemanden zu beleidigen oder zu mobben?
19. Standen Sie so unter Druck, dass Sie andere Menschen beschimpften, um diesen Druck abzubauen?
20. Fühlten Sie sich stark, wenn Sie jemanden körperlich angriffen?
21. Schlugen Sie zu, wenn Sie sich in die Enge getrieben fühlten?
22. Waren Sie gemein und mussten Sie das nächste Mal gemeiner sein, um wieder die positive Aufregung zu spüren?
23. Fühlten Sie sich erleichtert, wenn Sie jemanden anschrien?
24. Faszinierte Sie eine Prügelei so sehr, dass Sie nicht aufhören konnten, sich zu prügeln?
25. Wenn Sie sich ärgerten, schlugen Sie zu, um Ihrem Ärger Luft zu machen?
26. Beschimpften oder beleidigten Sie andere, um sich gut zu fühlen?
27. Ließen Sie andere Ihren Ärger spüren, wenn Sie wegen denen nicht das bekamen, was Sie wollten?
28. Gab Ihnen eine Prügelei ein so gutes Gefühl, dass Sie auch Verletzungen in Kauf nahmen?
29. Fühlten Sie sich besser, wenn Sie jemanden schlugen, durch den Sie sich bedroht fühlten?
30. Zerstörten Sie Dinge, wenn Ihnen etwas weh tat
```

### find all files¶

In [3]:

```
def get_logfile(whichfolder, whichexperiment):

    loglist = []
    for fileName in os.listdir(whichfolder):
        if fnmatch.fnmatch(fileName, whichexperiment):
            loglist.append(whichfolder+fileName)
    return loglist
```

In [4]:

```
loglist = get_logfile('../experiment/quest/app/static/logfiles/','log*.txt')
loglist.sort()
```

Example:

In [5]:

```
loglist[:5]
```

Out[5]:

```
['../experiment/quest/app/static/logfiles/logfile_A1.txt',
 '../experiment/quest/app/static/logfiles/logfile_A10.txt',
 '../experiment/quest/app/static/logfiles/logfile_A11.txt',
 '../experiment/quest/app/static/logfiles/logfile_A12.txt',
 '../experiment/quest/app/static/logfiles/logfile_A13.txt']
```

### get data of one participant¶

In [6]:

```
def makeThis(logfile):

    pName = logfile[logfile.find('_')+1:logfile.rfind('.')]
    pName = pName[0] + ('000'+ pName[1:])[-3:]
    
    fullDf = pd.read_csv(logfile,
                         skiprows=2,
                         index_col=[0]
                        )
    
    fullDf.index = [[pName]*len(fullDf.index),fullDf.index]
    fullDf.index.names = ['id', 'f']
    fullDf.columns = ['ans']
    
    withinDf = fullDf.unstack(1)
    
    # sort columns
    newCols = []
    for entry in withinDf.columns.levels[1]:
        newName = '00'+str(entry[1:])
        newCols.append('f'+newName[-3:])
    withinDf.columns = newCols
    withinDf = withinDf.reindex_axis(sorted(withinDf.columns), axis=1)
    
    return withinDf
```

Example:

In [7]:

```
makeThis(loglist[-1])
```

Out[7]:

|  | f001 | f002 | f003 | f004 | f005 | f006 | f007 | f008 | f009 | f010 | ... | f021 | f022 | f023 | f024 | f025 | f026 | f027 | f028 | f029 | f030 |
| --- | --- | --- | --- | --- | --- | --- | --- | --- | --- | --- | --- | --- | --- | --- | --- | --- | --- | --- | --- | --- | --- |
| id |  |  |  |  |  |  |  |  |  |  |  |  |  |  |  |  |  |  |  |  |  |
| K009 | 1 | 0 | 0 | 2 | 0 | 0 | 0 | 1 | 1 | 1 | ... | 1 | 0 | 1 | 0 | 1 | 0 | 1 | 0 | 1 | 1 |

1 rows × 30 columns

### get data of all participants¶

In [8]:

```
def makeAll(loglist):
    
    for p in loglist:
        thisDf = makeThis(p)
        try:
            bigDf = pd.concat([bigDf,thisDf])
            
        except:
            bigDf = thisDf
    
    group = [a[0] for a in bigDf.index]
    
    bigDf.index = [group,bigDf.index]
    bigDf = bigDf.sort_index(axis=0,level=1)
    
    return bigDf
```

In [9]:

```
bigDf = makeAll(loglist)
```

The final DataFrame:

In [10]:

```
bigDf.head()
```

Out[10]:

|  |  | f001 | f002 | f003 | f004 | f005 | f006 | f007 | f008 | f009 | f010 | ... | f021 | f022 | f023 | f024 | f025 | f026 | f027 | f028 | f029 | f030 |
| --- | --- | --- | --- | --- | --- | --- | --- | --- | --- | --- | --- | --- | --- | --- | --- | --- | --- | --- | --- | --- | --- | --- |
|  | id |  |  |  |  |  |  |  |  |  |  |  |  |  |  |  |  |  |  |  |  |  |
| A | A001 | 1 | 0 | 1 | 0 | 1 | 0 | 0 | 0 | 0 | 1 | ... | 1 | 0 | 2 | 0 | 0 | 1 | 1 | 0 | 2 | 0 |
|  | A002 | 1 | 0 | 0 | 0 | 1 | 0 | 1 | 0 | 0 | 0 | ... | 0 | 0 | 1 | 0 | 0 | 0 | 0 | 0 | 0 | 0 |
|  | A003 | 2 | 0 | 0 | 0 | 1 | 0 | 1 | 0 | 1 | 1 | ... | 1 | 0 | 1 | 0 | 0 | 0 | 0 | 0 | 0 | 2 |
|  | A004 | 0 | 0 | 0 | 1 | 0 | 0 | 2 | 1 | 0 | 0 | ... | 0 | 0 | 1 | 0 | 0 | 0 | 1 | 0 | 0 | 0 |
|  | A005 | 3 | 0 | 1 | 3 | 2 | 3 | 1 | 4 | 4 | 0 | ... | 0 | 0 | 1 | 0 | 0 | 3 | 1 | 1 | 2 | 3 |

5 rows × 30 columns

### Export as CSV for re-use¶

In [11]:

```
bigDf.to_csv('../outputs/fullAFAS.csv')
```

Example of re-importing:

In [12]:

```
pd.read_csv('../outputs/fullAFAS.csv',
            index_col =[0,1]).head()
```

Out[12]:

|  |  | f001 | f002 | f003 | f004 | f005 | f006 | f007 | f008 | f009 | f010 | ... | f021 | f022 | f023 | f024 | f025 | f026 | f027 | f028 | f029 | f030 |
| --- | --- | --- | --- | --- | --- | --- | --- | --- | --- | --- | --- | --- | --- | --- | --- | --- | --- | --- | --- | --- | --- | --- |
|  | id |  |  |  |  |  |  |  |  |  |  |  |  |  |  |  |  |  |  |  |  |  |
| A | A001 | 1 | 0 | 1 | 0 | 1 | 0 | 0 | 0 | 0 | 1 | ... | 1 | 0 | 2 | 0 | 0 | 1 | 1 | 0 | 2 | 0 |
|  | A002 | 1 | 0 | 0 | 0 | 1 | 0 | 1 | 0 | 0 | 0 | ... | 0 | 0 | 1 | 0 | 0 | 0 | 0 | 0 | 0 | 0 |
|  | A003 | 2 | 0 | 0 | 0 | 1 | 0 | 1 | 0 | 1 | 1 | ... | 1 | 0 | 1 | 0 | 0 | 0 | 0 | 0 | 0 | 2 |
|  | A004 | 0 | 0 | 0 | 1 | 0 | 0 | 2 | 1 | 0 | 0 | ... | 0 | 0 | 1 | 0 | 0 | 0 | 1 | 0 | 0 | 0 |
|  | A005 | 3 | 0 | 1 | 3 | 2 | 3 | 1 | 4 | 4 | 0 | ... | 0 | 0 | 1 | 0 | 0 | 3 | 1 | 1 | 2 | 3 |

5 rows × 30 columns

## Get avarage scores for each participant¶

Facilitative Aggression:
Items 1,3,5,8,10,12,14,17,19,21,23,25,27,29,30

Appetitive Aggression:
Items 2,4,6,7,9,11,13,15,16,18,20,22,24,26,28

In [13]:

```
fAgr=np.array([1,3,5,8,10,12,14,17,19,21,23,25,27,29,30])-1 # because we start counting from zero, we subtract 1
aAgr=np.array([2,4,6,7,9,11,13,15,16,18,20,22,24,26,28])-1

print fAgr
print aAgr
```

```
[ 0  2  4  7  9 11 13 16 18 20 22 24 26 28 29]
[ 1  3  5  6  8 10 12 14 15 17 19 21 23 25 27]
```

Example: facilitative aggression

In [14]:

```
bigDf[bigDf.columns[fAgr]].head()
```

Out[14]:

|  |  | f001 | f003 | f005 | f008 | f010 | f012 | f014 | f017 | f019 | f021 | f023 | f025 | f027 | f029 | f030 |
| --- | --- | --- | --- | --- | --- | --- | --- | --- | --- | --- | --- | --- | --- | --- | --- | --- |
|  | id |  |  |  |  |  |  |  |  |  |  |  |  |  |  |  |
| A | A001 | 1 | 1 | 1 | 0 | 1 | 2 | 1 | 1 | 1 | 1 | 2 | 0 | 1 | 2 | 0 |
|  | A002 | 1 | 0 | 1 | 0 | 0 | 0 | 0 | 0 | 1 | 0 | 1 | 0 | 0 | 0 | 0 |
|  | A003 | 2 | 0 | 1 | 0 | 1 | 1 | 0 | 0 | 0 | 1 | 1 | 0 | 0 | 0 | 2 |
|  | A004 | 0 | 0 | 0 | 1 | 0 | 0 | 0 | 1 | 0 | 0 | 1 | 0 | 1 | 0 | 0 |
|  | A005 | 3 | 1 | 2 | 4 | 0 | 3 | 0 | 1 | 0 | 0 | 1 | 0 | 1 | 2 | 3 |

In [15]:

```
def makeMean(df,fAgr=fAgr,aAgr=aAgr,labelCoding=labelCoding):
    # get the mean on both scales
    meanF = df[df.columns[fAgr]].T.mean()
    meanA = df[df.columns[aAgr]].T.mean()
    # get the overall mean
    meanBig = df.T.mean()
    
    assert meanBig.all() == ((meanA+meanF)/2.).all() , 'mean values corrupted; please check'
    
    # put them in one df
    meanDf = pd.DataFrame([meanF,meanA,meanBig]).T
    # structure the df
    meanDf['group'] = [ labelCoding[x[0]] for x in meanDf.index.levels[1] ]
    meanDf.columns = ['Facilitative','Appetitive','Overall','group']
    meanDf = meanDf.sort_index()
   
    return meanDf
```

In [16]:

```
meanDf = makeMean(bigDf)
```

In [17]:

```
meanDf.head()
```

Out[17]:

|  |  | Facilitative | Appetitive | Overall | group |
| --- | --- | --- | --- | --- | --- |
|  | id |  |  |  |  |
| A | A001 | 1.000000 | 0.266667 | 0.633333 | 2 |
|  | A002 | 0.266667 | 0.066667 | 0.166667 | 2 |
|  | A003 | 0.600000 | 0.200000 | 0.400000 | 2 |
|  | A004 | 0.266667 | 0.200000 | 0.233333 | 2 |
|  | A005 | 1.400000 | 1.200000 | 1.300000 | 2 |

### Save for re-use:¶

In [18]:

```
meanDf.to_csv('../outputs/meanAFAS.csv')
```

## Plot of the results¶

In [19]:

```
sns.palplot(myPal)
```

In [20]:

```
sortDf = meanDf.sort_values('group')
```

In [21]:

```
sortDf.tail()
```

Out[21]:

|  |  | Facilitative | Appetitive | Overall | group |
| --- | --- | --- | --- | --- | --- |
|  | id |  |  |  |  |
| A | A014 | 1.000000 | 0.533333 | 0.766667 | 2 |
|  | A015 | 0.200000 | 0.066667 | 0.133333 | 2 |
|  | A016 | 0.133333 | 0.200000 | 0.166667 | 2 |
|  | A010 | 0.600000 | 0.200000 | 0.400000 | 2 |
|  | A001 | 1.000000 | 0.266667 | 0.633333 | 2 |

In [22]:

```
fig = plt.figure(figsize=(15,6))

for i,metric in enumerate(meanDf.columns[:-1]):
    
    ax = fig.add_subplot(int('13'+str(i+1)))

    sns.boxplot(x='group',y=metric,data=sortDf,
                width=0.4,linewidth=1,color='white',whis=True,notch=True,fliersize=0,ax=ax)

    sns.stripplot(x='group', y=metric, data=sortDf,
                  jitter=True, edgecolor='white',palette=myPal,size=9,linewidth=1,ax=ax)

    ax.axhline(0,color='k',linewidth=1)
    ax.set_title(metric)
    
    ax.set_xticklabels(['Violence\nOffenders','Child\nMolesters','General\nPopulation'],fontsize=15)
    #ax.set_xticks(range(3),['Violence\nOffenders','Child\nMolesters','General\nPopulation'])
    ax.set_xlabel('')
    if i ==  0: 
        ax.set_ylabel('Mean Score (0-4 scale)')
    else:
        ax.set_ylabel('')
    ax.set_ylim(-0.2,4)
    sns.despine()
    
plt.suptitle('AFAS Scores',fontsize=20, position=(0.51,1.1))
# save to file
plt.savefig('../figures/afasFig.png',dpi=300) 
# show in notebook
plt.show()
```

### Inferential statistics¶

In [23]:

```
def makeUTests(df):
    bigDf = pd.DataFrame()
    d = {}
    for comp in [('G','K'),('G','A'),('K','A')]:
        for variable in df.columns[:-1]:
            group1 = df.ix[comp[0]][variable]
            group2 = df.ix[comp[1]][variable]
            U,p = stats.mannwhitneyu(group1,group2)
            if p<0.05:
                thisSig = '*'
            else:
                thisSig = 'n.s.'
            d[variable] = {'U':round(U,2),'p':round(p,3),'sig':thisSig}
        thisDf = pd.DataFrame(d)
        thisDf = thisDf.reindex_axis(['U','p','sig'], axis=0)
        thisDf.index = [ [comp[0]+' > '+comp[1]]*len(thisDf.index),thisDf.index ]

        bigDf = pd.concat([bigDf,thisDf])
    return bigDf.T
```

In [24]:

```
makeUTests(meanDf)
```

Out[24]:

|  | G > K | | | G > A | | | K > A | | |
| --- | --- | --- | --- | --- | --- | --- | --- | --- | --- |
|  | U | p | sig | U | p | sig | U | p | sig |
| Appetitive | 322.5 | 0.005 | \* | 360 | 0.02 | \* | 90 | 0.253 | n.s. |
| Facilitative | 329 | 0.003 | \* | 357.5 | 0.024 | \* | 80 | 0.125 | n.s. |
| Overall | 331 | 0.002 | \* | 362.5 | 0.018 | \* | 89 | 0.241 | n.s. |

### Investigating how the subscales correlate with other¶

In [25]:

```
# Draw a categorical scatterplot to show each observation
sns.lmplot(x="Facilitative", y="Appetitive", hue="group", data=sortDf,palette=myPal);
```

In [26]:

```
meanDf.groupby("group").describe().T[[1,2,9,10,17,18]]
```

Out[26]:

| group | 0 | | 1 | | 2 | |
| --- | --- | --- | --- | --- | --- | --- |
|  | mean | std | mean | std | mean | std |
| Appetitive | 0.964444 | 0.874103 | 0.252381 | 0.265611 | 0.329412 | 0.288165 |
| Facilitative | 1.264444 | 0.924504 | 0.466667 | 0.361384 | 0.650980 | 0.411319 |
| Overall | 1.114444 | 0.876893 | 0.359524 | 0.272744 | 0.490196 | 0.329723 |

# PPI-R¶

In [27]:

```
ppiDf = pd.read_csv('../experiment/ppi_r.csv',
                   index_col=[0,1])

ppiDf['group'] = [ labelCoding[x[0]] for x in ppiDf.index.levels[1]]
ppiDf = ppiDf.replace(-99,np.nan)
ppiDf = ppiDf.dropna()
ppiDf = ppiDf
```

In [28]:

```
ppiDf.head()
```

Out[28]:

|  |  | Blame externalization | Rebellious nonconformity | Stress immunity | Social influence | Coldheartedness | Machiavellian egocentricity | Carefree nonplanfulness | Fearlessness | Sum PPI-R | Insincere Answers | group |
| --- | --- | --- | --- | --- | --- | --- | --- | --- | --- | --- | --- | --- |
| g | id |  |  |  |  |  |  |  |  |  |  |  |
| A | A001 | 18.0 | 57.0 | 46.0 | 53.0 | 27.0 | 35.0 | 24.0 | 25.0 | 285.0 | 53 | 2 |
|  | A002 | 22.0 | 41.0 | 46.0 | 46.0 | 30.0 | 32.0 | 30.0 | 9.0 | 256.0 | 43 | 2 |
|  | A003 | 34.0 | 56.0 | 37.0 | 46.0 | 32.0 | 31.0 | 30.0 | 24.0 | 290.0 | 41 | 2 |
|  | A004 | 19.0 | 46.0 | 51.0 | 34.0 | 38.0 | 32.0 | 22.0 | 18.0 | 260.0 | 47 | 2 |
|  | A005 | 45.0 | 82.0 | 37.0 | 37.0 | 37.0 | 46.0 | 38.0 | 27.0 | 349.0 | 31 | 2 |

In [29]:

```
ppiDf.to_csv('../outputs/ppiDf.csv')
```

In [30]:

```
def makeBoxPlot(df):
    fig = plt.figure(figsize=(15,8))

    sortDf = df.sort_values('group')
    
    myColumns = df.columns[:-1]
    numColumns = len(myColumns)
    
    for i,metric in enumerate(myColumns):

        ax = fig.add_subplot(2,numColumns/2.,i+1)

        sns.boxplot(x='group',y=metric,data=sortDf,
                    width=0.4,linewidth=1,color='white',whis=True,notch=True,fliersize=0,ax=ax)

        sns.stripplot(x='group', y=metric, data=sortDf,
                      jitter=True, edgecolor='white',palette=myPal,ax=ax)

        ax.set_title(metric,fontsize=15)

        ax.set_xticklabels(['Violence\nOffenders','Child\nMolesters','General\nPopulation'],fontsize=9)
        ax.set_xlabel('')
        if i%5 ==  0: 
            ax.set_ylabel('Sum Score',fontsize=15)
        else:
            ax.set_ylabel('')
        #ax.set_ylim(-0.2,4)
        sns.despine()

    plt.suptitle('PPI-R Scores',fontsize=20, position=(0.51,1.02))
    # save to file
    #plt.savefig('../figures/afasFig.png',dpi=300) 
    # show in notebook
    plt.tight_layout()
    plt.show()
```

In [31]:

```
makeBoxPlot(ppiDf)
```

In [32]:

```
ppiDf.groupby("group").describe().T[[1,2,9,10,17,18]]
```

Out[32]:

| group | 0 | | 1 | | 2 | |
| --- | --- | --- | --- | --- | --- | --- |
|  | mean | std | mean | std | mean | std |
| Blame externalization | 33.172414 | 9.606802 | 37.923077 | 8.836115 | 26.647059 | 7.565887 |
| Carefree nonplanfulness | 27.551724 | 5.520004 | 31.153846 | 6.053183 | 29.647059 | 6.660904 |
| Coldheartedness | 32.758621 | 5.841081 | 29.615385 | 3.819652 | 30.529412 | 5.917323 |
| Fearlessness | 17.827586 | 5.910457 | 15.384615 | 5.058631 | 18.176471 | 5.491303 |
| Insincere Answers | 41.758621 | 6.456753 | 45.153846 | 7.278349 | 41.588235 | 7.141943 |
| Machiavellian egocentricity | 34.620690 | 6.008410 | 33.846154 | 5.459642 | 33.647059 | 4.755801 |
| Rebellious nonconformity | 54.310345 | 16.444937 | 50.538462 | 10.689679 | 53.000000 | 13.486104 |
| Social influence | 44.827586 | 8.888472 | 34.538462 | 8.903327 | 45.588235 | 5.916701 |
| Stress immunity | 44.517241 | 9.649651 | 44.538462 | 8.809232 | 43.000000 | 6.855655 |
| Sum PPI-R | 289.586207 | 34.983177 | 277.538462 | 29.452265 | 280.235294 | 27.148963 |

### Inferential statistics¶

In [33]:

```
makeUTests(ppiDf)
```

Out[33]:

|  | G > K | | | G > A | | | K > A | | |
| --- | --- | --- | --- | --- | --- | --- | --- | --- | --- |
|  | U | p | sig | U | p | sig | U | p | sig |
| Blame externalization | 132 | 0.127 | n.s. | 349 | 0.02 | \* | 182.5 | 0.003 | \* |
| Carefree nonplanfulness | 120 | 0.064 | n.s. | 195.5 | 0.249 | n.s. | 124.5 | 0.571 | n.s. |
| Coldheartedness | 254.5 | 0.074 | n.s. | 284.5 | 0.392 | n.s. | 94 | 0.501 | n.s. |
| Fearlessness | 241 | 0.156 | n.s. | 246.5 | 0.991 | n.s. | 78.5 | 0.185 | n.s. |
| Insincere Answers | 121.5 | 0.07 | n.s. | 249 | 0.964 | n.s. | 145.5 | 0.148 | n.s. |
| Machiavellian egocentricity | 193 | 0.913 | n.s. | 272 | 0.568 | n.s. | 121 | 0.674 | n.s. |
| Rebellious nonconformity | 203 | 0.703 | n.s. | 237.5 | 0.847 | n.s. | 102 | 0.737 | n.s. |
| Social influence | 294 | 0.004 | \* | 220 | 0.553 | n.s. | 34 | 0.001 | \* |
| Stress immunity | 212 | 0.531 | n.s. | 285.5 | 0.38 | n.s. | 117 | 0.801 | n.s. |
| Sum PPI-R | 219 | 0.414 | n.s. | 289.5 | 0.333 | n.s. | 108 | 0.933 | n.s. |

# SCID-II¶

In [34]:

```
scidDf = pd.read_csv('../experiment/scid_ii.csv',
                   index_col=[0,1])

scidDf = scidDf.replace(-99,np.nan)
scidDf = scidDf.dropna()
scidDf = scidDf[:-1]
```

In [35]:

```
scidDf.to_csv('../outputs/scidDf.csv')
```

In [36]:

```
scidDf.tail()
```

Out[36]:

|  |  | Avoidant | Dependent | OCD | Negativistic | Depressive | Paranoid | Schizotypal | Schizoid | Histrionic | Narcissitic | Borderline | Antisocial | group |
| --- | --- | --- | --- | --- | --- | --- | --- | --- | --- | --- | --- | --- | --- | --- |
| g | id |  |  |  |  |  |  |  |  |  |  |  |  |  |
| K | K010 | 5 | 5 | 7 | 7 | 7 | 8 | 7 | 4 | 0 | 11 | 10 | 7 | 1 |
|  | K011 | 1 | 0 | 3 | 0 | 0 | 1 | 0 | 1 | 0 | 1 | 0 | 0 | 1 |
|  | K012 | 2 | 0 | 2 | 2 | 2 | 5 | 7 | 1 | 0 | 2 | 3 | 0 | 1 |
|  | K013 | 4 | 6 | 5 | 0 | 3 | 4 | 2 | 2 | 1 | 4 | 0 | 2 | 1 |
|  | K014 | 0 | 2 | 5 | 2 | 2 | 0 | 0 | 0 | 0 | 0 | 0 | 0 | 1 |

In [37]:

```
def makeScidPlot(df):
    fig = plt.figure(figsize=(15,9))
    sns.set_style('ticks')

    sortDf = df.sort_values('group')
    
    myColumns = df.columns[:-1]
    numColumns = len(myColumns)
    
    for i,metric in enumerate(myColumns):

        ax = fig.add_subplot(3,4,i+1)

        sns.boxplot(x='group',y=metric,data=sortDf,
                    width=0.4,linewidth=1,color='white',whis=True,notch=True,fliersize=0,ax=ax)

        sns.stripplot(x='group', y=metric, data=sortDf,
                      jitter=True, edgecolor='white',palette=myPal,ax=ax)

        ax.set_title(metric)

        ax.set_xticklabels(['Violence\nOffenders','Child\nMolesters','General\nPopulation'],fontsize=9)
        #ax.set_xticks(range(3),['Violence\nOffenders','Child\nMolesters','General\nPopulation'])
        ax.set_xlabel('')
        if i%4 ==  0: 
            ax.set_ylabel('Sum Score',fontsize=15)
        else:
            ax.set_ylabel('')
        #ax.set_ylim(-0.2,4)
        sns.despine()
    plt.suptitle('SCID-II',fontsize=20, position=(0.51,1.02))
    # save to file
    #plt.savefig('../figures/afasFig.png',dpi=300) 
    # show in notebook
    plt.tight_layout()
    plt.show()
```

In [38]:

```
makeScidPlot(scidDf)
```

In [39]:

```
scidDf.groupby("group").describe().T[[1,2,9,10,17,18]]
```

Out[39]:

| group | 0 | | 1 | | 2 | |
| --- | --- | --- | --- | --- | --- | --- |
|  | mean | std | mean | std | mean | std |
| Antisocial | 4.233333 | 4.190986 | 2.428571 | 2.208873 | 1.823529 | 2.811479 |
| Avoidant | 1.500000 | 1.696853 | 2.357143 | 1.984833 | 0.764706 | 1.091410 |
| Borderline | 3.533333 | 3.234868 | 2.214286 | 2.887069 | 2.470588 | 2.648529 |
| Dependent | 1.400000 | 1.522249 | 2.142857 | 1.833750 | 1.117647 | 1.053705 |
| Depressive | 1.833333 | 2.069205 | 3.071429 | 2.335818 | 0.882353 | 1.536325 |
| Histrionic | 1.600000 | 1.811838 | 0.571429 | 0.755929 | 1.411765 | 1.325652 |
| Narcissitic | 4.000000 | 2.936101 | 3.071429 | 3.149219 | 2.176471 | 1.776066 |
| Negativistic | 1.566667 | 1.675036 | 2.071429 | 1.899971 | 1.235294 | 1.437420 |
| OCD | 3.600000 | 1.631585 | 4.428571 | 1.785165 | 4.294118 | 2.114377 |
| Paranoid | 2.566667 | 2.095699 | 2.857143 | 2.567763 | 1.352941 | 1.656094 |
| Schizoid | 1.800000 | 1.423933 | 2.642857 | 1.984833 | 1.588235 | 1.003670 |
| Schizotypal | 1.233333 | 0.935261 | 2.142857 | 2.381245 | 1.411765 | 1.371989 |

### inferential statistics¶

In [40]:

```
makeUTests(scidDf)
```

Out[40]:

|  | G > K | | | G > A | | | K > A | | |
| --- | --- | --- | --- | --- | --- | --- | --- | --- | --- |
|  | U | p | sig | U | p | sig | U | p | sig |
| Antisocial | 250.5 | 0.308 | n.s. | 355 | 0.025 | \* | 147.5 | 0.252 | n.s. |
| Avoidant | 154 | 0.151 | n.s. | 314 | 0.171 | n.s. | 177.5 | 0.016 | \* |
| Borderline | 273.5 | 0.108 | n.s. | 309.5 | 0.227 | n.s. | 107.5 | 0.653 | n.s. |
| Dependent | 155.5 | 0.15 | n.s. | 275 | 0.642 | n.s. | 159 | 0.1 | n.s. |
| Depressive | 137 | 0.063 | n.s. | 331.5 | 0.077 | n.s. | 194 | 0.002 | \* |
| Histrionic | 279 | 0.069 | n.s. | 256 | 0.991 | n.s. | 76 | 0.074 | n.s. |
| Narcissitic | 255.5 | 0.253 | n.s. | 349.5 | 0.036 | \* | 132 | 0.615 | n.s. |
| Negativistic | 169.5 | 0.3 | n.s. | 285.5 | 0.49 | n.s. | 153.5 | 0.164 | n.s. |
| OCD | 152.5 | 0.145 | n.s. | 195 | 0.182 | n.s. | 120 | 0.984 | n.s. |
| Paranoid | 198.5 | 0.778 | n.s. | 348.5 | 0.035 | \* | 169 | 0.044 | \* |
| Schizoid | 158.5 | 0.188 | n.s. | 274 | 0.669 | n.s. | 155 | 0.144 | n.s. |
| Schizotypal | 178.5 | 0.408 | n.s. | 246 | 0.841 | n.s. | 133.5 | 0.568 | n.s. |
