# Supplementary material for: In your face: the biased judgement of fear-anger expressions in violent offenders
Source: BMC Psychol. 2017 May 12;5:16. doi: 10.1186/s40359-017-0186-z (PMC5429544; doi:10.1186/s40359-017-0186-z)
Supplement: Supplementary file 6 — Analysis of basic expression recognition performance. (HTML 489 kb) [file 40359_2017_186_MOESM6_ESM.html]

002\_basicExpressions


# In your face: Biased judgement of fear-anger expressions in violence offenders.

# 2. Analysis of Basic Emotion Recognition¶

This is an analysis of a basic emotion recognition paradigm, where 12 faces (6 female, 6 male) were shown to the participants, each displaying all basic expressions (happy, sad, angry, fearful, disgusted, suprised + neutral).  
Hence, there are 12 trials per expression and 12\*7=84 trials in total for each participant.

### importing modules¶

In [1]:

```
import numpy as np
import pandas as pd

import os
import fnmatch

import seaborn as sns
import matplotlib.pyplot as plt
%matplotlib inline

from myBasics import *
```

### get logfiles¶

In [2]:

```
def getLogfile(whichfolder, whichexperiment):

    loglist = []
    for fileName in os.listdir(whichfolder):
        if fnmatch.fnmatch(fileName, whichexperiment):
            loglist.append(whichfolder+fileName)
    return loglist
```

In [3]:

```
logList = getLogfile('../experiment/data/','*facesParametric*.csv')
logList.sort()
```

Example:

In [4]:

```
logList[:5]
```

Out[4]:

```
['../experiment/data/A_10_facesParametric_2015_Sep_20_1307.csv',
 '../experiment/data/A_11_facesParametric_2015_Sep_25_1753.csv',
 '../experiment/data/A_12_facesParametric_2015_Oct_11_1221.csv',
 '../experiment/data/A_13_facesParametric_2015_Oct_11_1620.csv',
 '../experiment/data/A_14_facesParametric_2015_Nov_01_1250.csv']
```

### Extract Responses from logfile¶

In [5]:

```
def getBasicResps(fileName):
    df = pd.read_csv(fileName)

    ## merge scales into one column
    
    # convert None to NaN
    df = df.replace(r'None', np.nan, regex=True)
    df['basicRating1.response'] = df['basicRating1.response'].fillna(df['basicRating2.response']).fillna(df['basicRating3.response']).fillna(df['basicRating4.response'])
    df['basicRating1.rt'] = df['basicRating1.rt'].fillna(df['basicRating2.rt']).fillna(df['basicRating3.rt']).fillna(df['basicRating4.rt'])
   
    # boil down to just the essentials
    thisDf = pd.concat([df['img'],
                        df['basicRating1.response'],
                        df['basicRating1.rt']
                       ],axis=1)
    
    return thisDf[1:85]
```

Example:

In [6]:

```
getBasicResps(logList[-1]).head()
```

Out[6]:

|  | img | basicRating1.response | basicRating1.rt |
| --- | --- | --- | --- |
| 1 | ./basicStim/21M\_DI\_O.jpg | HAP | 18.338 |
| 2 | ./basicStim/28M\_HA\_O.jpg | HAP | 6.682 |
| 3 | ./basicStim/23M\_NE\_C.jpg | ANG | 3.406 |
| 4 | ./basicStim/07F\_FE\_O.jpg | DIS | 2.154 |
| 5 | ./basicStim/07F\_HA\_O.jpg | HAP | 1.73 |

## Get Conditions from Filenames¶

In [7]:

```
def getConds(df):
    
    df['conds'] = [entry[entry.find('_')+1:entry.rfind('_')] for entry in df['img'] ]
    df = df.sort_values(by='conds')
    df.index = [df['conds'],df.index]
    
    return df
```

Example:

In [8]:

```
getConds(getBasicResps(logList[-1])).head()
```

Out[8]:

|  |  | img | basicRating1.response | basicRating1.rt | conds |
| --- | --- | --- | --- | --- | --- |
| conds |  |  |  |  |  |
| AN | 64 | ./basicStim/24M\_AN\_O.jpg | ANG | 2.939 | AN |
|  | 66 | ./basicStim/07F\_AN\_O.jpg | ANG | 2.871 | AN |
|  | 56 | ./basicStim/34M\_AN\_O.jpg | ANG | 2.016 | AN |
|  | 69 | ./basicStim/36M\_AN\_O.jpg | SUP | 2.318 | AN |
|  | 70 | ./basicStim/21M\_AN\_O.jpg | ANG | 2.809 | AN |

### Evaluate whether response is correct or not (binary)¶

In [9]:

```
def addEval(df):
    
    resp = []
    for index,entry in enumerate( df['basicRating1.response'] ):
        # for each correct response, append a one
        if entry=='ANG' and df['conds'][index]=='AN':
            resp.append(1)
        elif entry=='ANG' and df['conds'][index]=='AN':
            resp.append(1)
        elif entry=='DIS' and df['conds'][index]=='DI':
            resp.append(1)
        elif entry=='FEA' and df['conds'][index]=='FE':
            resp.append(1)
        elif entry=='HAP' and df['conds'][index]=='HA':
            resp.append(1)
        elif entry=='NTR' and df['conds'][index]=='NE':
            resp.append(1)
        elif entry=='SAD' and df['conds'][index]=='SA':
            resp.append(1)
        elif entry=='SUP' and df['conds'][index]=='SP':
            resp.append(1)   
        # all other responses must be incorrect; we append a zero
        else:
            resp.append(0)

    df['resp'] = resp
        
    return df
```

Example:

In [10]:

```
addEval(getConds(getBasicResps(logList[-1]))).head()
```

Out[10]:

|  |  | img | basicRating1.response | basicRating1.rt | conds | resp |
| --- | --- | --- | --- | --- | --- | --- |
| conds |  |  |  |  |  |  |
| AN | 64 | ./basicStim/24M\_AN\_O.jpg | ANG | 2.939 | AN | 1 |
|  | 66 | ./basicStim/07F\_AN\_O.jpg | ANG | 2.871 | AN | 1 |
|  | 56 | ./basicStim/34M\_AN\_O.jpg | ANG | 2.016 | AN | 1 |
|  | 69 | ./basicStim/36M\_AN\_O.jpg | SUP | 2.318 | AN | 0 |
|  | 70 | ./basicStim/21M\_AN\_O.jpg | ANG | 2.809 | AN | 1 |

### Average over trials¶

Count and average the correct responses. Add a participant number to the index.

In [11]:

```
def getAvg(df,p):
    meanDf = pd.DataFrame([df.ix[cond]['resp'].mean()] for cond in df.index.levels[0])
    meanDf['cond'] = [cond for cond in df.index.levels[0]]
    meanDf.index = [[p]*len(meanDf.index), meanDf['cond'] ]
    meanDf.index.names = ['id','cond']
    
    return meanDf
```

Example:

In [12]:

```
getAvg(addEval(getConds(getBasicResps(logList[-1]))),'pXYZ').head()
```

Out[12]:

|  |  | 0 | cond |
| --- | --- | --- | --- |
| id | cond |  |  |
| pXYZ | AN | 0.666667 | AN |
|  | DI | 0.666667 | DI |
|  | FE | 0.166667 | FE |
|  | HA | 1.000000 | HA |
|  | NE | 0.750000 | NE |

### Do all this for a participant¶

Takes all the functions from above and applies them directly to a logfile. Also gets the participant number form the logfile name.

In [13]:

```
def makeOne(fileName):
    df = getBasicResps(fileName)

    condDf = getConds(df)
    
    respDf = addEval(condDf)
    
    p = fileName[fileName.find('_')-1:fileName.find('faces')-1]
    avgDf = getAvg(respDf,p)
    return avgDf
```

Example:

In [14]:

```
makeOne(logList[-1])
```

Out[14]:

|  |  | 0 | cond |
| --- | --- | --- | --- |
| id | cond |  |  |
| K\_9 | AN | 0.666667 | AN |
|  | DI | 0.666667 | DI |
|  | FE | 0.166667 | FE |
|  | HA | 1.000000 | HA |
|  | NE | 0.750000 | NE |
|  | SA | 0.083333 | SA |
|  | SP | 0.833333 | SP |

## Do this for whole group¶

In [15]:

```
def makeBig(loglist):
    for logfile in loglist:
        thisDf = makeOne(logfile)
        withinDf = thisDf.drop('cond',1).unstack()
        
        try:
            bigDf = pd.concat([bigDf,withinDf])
        except:
            bigDf = withinDf
    
    groups = [a[0] for a in bigDf.index]
    
    bigDf.index = [groups, bigDf.index ]
    bigDf.index.names = ['group','id']
    bigDf.columns = bigDf.columns.droplevel()
    return bigDf
```

This is the main table for the analyses:

In [16]:

```
bigDf = makeBig(logList)
```

In [17]:

```
bigDf.head()
```

Out[17]:

|  | cond | AN | DI | FE | HA | NE | SA | SP |
| --- | --- | --- | --- | --- | --- | --- | --- | --- |
| group | id |  |  |  |  |  |  |  |
| A | A\_10 | 1.000000 | 0.750000 | 0.416667 | 1.000000 | 0.916667 | 0.916667 | 0.500000 |
|  | A\_11 | 0.916667 | 0.916667 | 0.833333 | 1.000000 | 0.666667 | 0.666667 | 0.916667 |
|  | A\_12 | 0.666667 | 0.666667 | 0.083333 | 1.000000 | 0.916667 | 0.750000 | 0.833333 |
|  | A\_13 | 0.833333 | 0.916667 | 0.416667 | 1.000000 | 0.916667 | 0.750000 | 1.000000 |
|  | A\_14 | 1.000000 | 0.833333 | 0.250000 | 0.916667 | 1.000000 | 0.750000 | 0.916667 |

Save as csv for later re-use:

In [18]:

```
bigDf.to_csv('../outputs/basicExpressionsCorrect.csv')
```

## Basic Plotting¶

In [19]:

```
def makeBar(bigDf):
    fig = plt.figure(figsize=(12,8))
    add = 0
    for index,group in enumerate(bigDf.index.levels[0]):

        plt.bar(np.arange(0,14,2)+add,
                bigDf.ix[group].mean(),
                yerr=bigDf.ix[group].std()/np.sqrt(len(bigDf.ix[group]))*1.96,
                color=myPal[labelCoding[group]],
                ecolor='k',
                label=group)

        add+=0.5

    plt.legend(loc='lower right',bbox_to_anchor=(1.2,0.1))
    plt.xticks(np.arange(0,14,2)+1, bigDf.columns )
    sns.despine()
    plt.show()
```

In [20]:

```
makeBar(bigDf)
```

## Responses with confusions¶

Extract information from dataFrame in a more systematic way:

In [21]:

```
def recodeResponses(df):
    g = []; e = []
    for i in df.index:
        thisImg = df.ix[i]['img']
        thisGender = thisImg[ thisImg.rfind('/')+3 ]
        thisEmo = thisImg[ thisImg.find('_')+1 : thisImg.find('_')+3 ]

        g.append(thisGender)
        e.append(thisEmo)
    
    df['fgender'] = g
    df['emo'] = e

    return df
```

Example:

In [22]:

```
df = getBasicResps(logList[-1])
```

In [23]:

```
recodeResponses(df).head()
```

Out[23]:

|  | img | basicRating1.response | basicRating1.rt | fgender | emo |
| --- | --- | --- | --- | --- | --- |
| 1 | ./basicStim/21M\_DI\_O.jpg | HAP | 18.338 | M | DI |
| 2 | ./basicStim/28M\_HA\_O.jpg | HAP | 6.682 | M | HA |
| 3 | ./basicStim/23M\_NE\_C.jpg | ANG | 3.406 | M | NE |
| 4 | ./basicStim/07F\_FE\_O.jpg | DIS | 2.154 | F | FE |
| 5 | ./basicStim/07F\_HA\_O.jpg | HAP | 1.73 | F | HA |

Get full range of responses to each expression:

In [24]:

```
def getAllResponses(df,p):
    
    d = {}

    # prepare dict
    for entry in df.index:
        d[df.ix[entry]['emo']] = { 'F':{'HAP':0,'SAD':0,'ANG':0,'FEA':0,'DIS':0,'SUP':0,'NTR':0},
                                   'M':{'HAP':0,'SAD':0,'ANG':0,'FEA':0,'DIS':0,'SUP':0,'NTR':0}
                                 }  
    # fill dict
    for entry in df.index:
        thisResp = df.ix[entry]['basicRating1.response']
        thisFace = df.ix[entry]['emo']
        thisFGender = df.ix[entry]['fgender']
        try:
            d[thisFace][thisFGender][thisResp] +=1
        except:
            pass


    bigDf = pd.DataFrame()
    for entry in d:
        thisDf = pd.DataFrame(d[entry])
        thisDf.index = [ [entry]*len(thisDf.index), thisDf.index ]
        bigDf = pd.concat([bigDf, thisDf])


    bigDf = bigDf.sort_index()
    bigDf = bigDf.T
    bigDf.index = [[p]*len(bigDf.index), bigDf.index]
    bigDf.index.names = ['pNum','fGender']

    return bigDf
```

Example:

In [25]:

```
getAllResponses(df,'pXXX')
```

Out[25]:

|  |  | AN | | | | | | | DI | | | ... | SA | | | SP | | | | | | |
| --- | --- | --- | --- | --- | --- | --- | --- | --- | --- | --- | --- | --- | --- | --- | --- | --- | --- | --- | --- | --- | --- | --- |
|  |  | ANG | DIS | FEA | HAP | NTR | SAD | SUP | ANG | DIS | FEA | ... | NTR | SAD | SUP | ANG | DIS | FEA | HAP | NTR | SAD | SUP |
| pNum | fGender |  |  |  |  |  |  |  |  |  |  |  |  |  |  |  |  |  |  |  |  |  |
| pXXX | F | 4 | 1 | 0 | 0 | 0 | 0 | 1 | 1 | 5 | 0 | ... | 1 | 1 | 2 | 0 | 0 | 0 | 1 | 0 | 0 | 5 |
|  | M | 4 | 1 | 0 | 0 | 0 | 0 | 1 | 0 | 3 | 0 | ... | 0 | 0 | 2 | 0 | 0 | 0 | 1 | 0 | 0 | 5 |

2 rows × 49 columns

In [26]:

```
pd.DataFrame( pd.DataFrame( getAllResponses(df,'pXXX').ix['pXXX'].ix['F'] ).unstack() )/6.*100
```

Out[26]:

|  | F | | | | | | |
| --- | --- | --- | --- | --- | --- | --- | --- |
|  | ANG | DIS | FEA | HAP | NTR | SAD | SUP |
| AN | 66.666667 | 16.666667 | 0.000000 | 0.000000 | 0.000000 | 0.000000 | 16.666667 |
| DI | 16.666667 | 83.333333 | 0.000000 | 0.000000 | 0.000000 | 0.000000 | 0.000000 |
| FE | 0.000000 | 33.333333 | 0.000000 | 0.000000 | 0.000000 | 0.000000 | 66.666667 |
| HA | 0.000000 | 0.000000 | 0.000000 | 100.000000 | 0.000000 | 0.000000 | 0.000000 |
| NE | 0.000000 | 0.000000 | 16.666667 | 0.000000 | 66.666667 | 0.000000 | 16.666667 |
| SA | 0.000000 | 33.333333 | 0.000000 | 0.000000 | 16.666667 | 16.666667 | 33.333333 |
| SP | 0.000000 | 0.000000 | 0.000000 | 16.666667 | 0.000000 | 0.000000 | 83.333333 |

### Showing full confusion matrix as heatmap¶

In [27]:

```
fig = plt.figure(figsize=(14,7))
for i,fGender in enumerate(['F','M']):
    confDf = pd.DataFrame( pd.DataFrame( getAllResponses(df,'pXXX').ix['pXXX'].ix[fGender] ).unstack() )/6.*100
    ax = plt.subplot('12'+str(i+1))
    sns.heatmap(confDf,square=True);
    ax.set_title(fGender)
plt.show()
```

### Do this for all participants¶

In [28]:

```
def confusionDf(loglist):
    allRespDf = pd.DataFrame()

    for logfile in loglist:
        
        rawDf = getBasicResps(logfile)
        pName = logfile[logfile.rfind('/')+1:logfile.find('_faces')]
        thisDf = getAllResponses( recodeResponses(rawDf),pName )

        allRespDf = pd.concat([allRespDf,thisDf])
    
    # some restructuring...
    allRespDf = allRespDf.unstack(1)
    groupIndex = [ x[0] for x in allRespDf.index ]
    allRespDf.index = [ groupIndex,allRespDf.index  ]
    allRespDf.columns = allRespDf.columns.swaplevel(0, 2)
    allRespDf.sortlevel(0, axis=1, inplace=True)
    allRespDf.columns = allRespDf.columns.swaplevel(1, 2)
    allRespDf.sortlevel(0, axis=1, inplace=True)
    return allRespDf/6. # divide by number of trials in each condition (12 faces)
```

In [29]:

```
confDf = confusionDf(logList)
```

Example:

In [30]:

```
confDf.head()
```

Out[30]:

|  | fGender | F | | | | | | | | | | ... | M | | | | | | | | | |
| --- | --- | --- | --- | --- | --- | --- | --- | --- | --- | --- | --- | --- | --- | --- | --- | --- | --- | --- | --- | --- | --- | --- |
|  |  | AN | | | | | | | DI | | | ... | SA | | | SP | | | | | | |
|  |  | ANG | DIS | FEA | HAP | NTR | SAD | SUP | ANG | DIS | FEA | ... | NTR | SAD | SUP | ANG | DIS | FEA | HAP | NTR | SAD | SUP |
|  | pNum |  |  |  |  |  |  |  |  |  |  |  |  |  |  |  |  |  |  |  |  |  |
| A | A\_1 | 1.000000 | 0.0 | 0.000000 | 0.0 | 0.0 | 0.0 | 0.0 | 0.166667 | 0.833333 | 0.000000 | ... | 0.000000 | 0.833333 | 0.000000 | 0.0 | 0.000000 | 0.166667 | 0.000000 | 0.0 | 0.0 | 0.833333 |
|  | A\_10 | 1.000000 | 0.0 | 0.000000 | 0.0 | 0.0 | 0.0 | 0.0 | 0.000000 | 0.833333 | 0.166667 | ... | 0.000000 | 0.833333 | 0.000000 | 0.0 | 0.166667 | 0.333333 | 0.000000 | 0.0 | 0.0 | 0.500000 |
|  | A\_11 | 1.000000 | 0.0 | 0.000000 | 0.0 | 0.0 | 0.0 | 0.0 | 0.000000 | 1.000000 | 0.000000 | ... | 0.000000 | 0.666667 | 0.000000 | 0.0 | 0.000000 | 0.000000 | 0.000000 | 0.0 | 0.0 | 1.000000 |
|  | A\_12 | 0.833333 | 0.0 | 0.166667 | 0.0 | 0.0 | 0.0 | 0.0 | 0.333333 | 0.666667 | 0.000000 | ... | 0.000000 | 0.500000 | 0.333333 | 0.0 | 0.000000 | 0.000000 | 0.166667 | 0.0 | 0.0 | 0.833333 |
|  | A\_13 | 0.833333 | 0.0 | 0.166667 | 0.0 | 0.0 | 0.0 | 0.0 | 0.000000 | 1.000000 | 0.000000 | ... | 0.166667 | 0.500000 | 0.000000 | 0.0 | 0.000000 | 0.000000 | 0.000000 | 0.0 | 0.0 | 1.000000 |

5 rows × 98 columns

### Split the big plot into one for female, one for male faces¶

In [31]:

```
fConfDf = confDf['F']
mConfDf = confDf['M']
```

In [32]:

```
fConfDf.head()
```

Out[32]:

|  |  | AN | | | | | | | DI | | | ... | SA | | | SP | | | | | | |
| --- | --- | --- | --- | --- | --- | --- | --- | --- | --- | --- | --- | --- | --- | --- | --- | --- | --- | --- | --- | --- | --- | --- |
|  |  | ANG | DIS | FEA | HAP | NTR | SAD | SUP | ANG | DIS | FEA | ... | NTR | SAD | SUP | ANG | DIS | FEA | HAP | NTR | SAD | SUP |
|  | pNum |  |  |  |  |  |  |  |  |  |  |  |  |  |  |  |  |  |  |  |  |  |
| A | A\_1 | 1.000000 | 0.0 | 0.000000 | 0.0 | 0.0 | 0.0 | 0.0 | 0.166667 | 0.833333 | 0.000000 | ... | 0.0 | 0.500000 | 0.166667 | 0.0 | 0.0 | 0.166667 | 0.000000 | 0.0 | 0.0 | 0.833333 |
|  | A\_10 | 1.000000 | 0.0 | 0.000000 | 0.0 | 0.0 | 0.0 | 0.0 | 0.000000 | 0.833333 | 0.166667 | ... | 0.0 | 1.000000 | 0.000000 | 0.0 | 0.0 | 0.333333 | 0.166667 | 0.0 | 0.0 | 0.500000 |
|  | A\_11 | 1.000000 | 0.0 | 0.000000 | 0.0 | 0.0 | 0.0 | 0.0 | 0.000000 | 1.000000 | 0.000000 | ... | 0.0 | 0.666667 | 0.000000 | 0.0 | 0.0 | 0.000000 | 0.166667 | 0.0 | 0.0 | 0.833333 |
|  | A\_12 | 0.833333 | 0.0 | 0.166667 | 0.0 | 0.0 | 0.0 | 0.0 | 0.333333 | 0.666667 | 0.000000 | ... | 0.0 | 1.000000 | 0.000000 | 0.0 | 0.0 | 0.000000 | 0.166667 | 0.0 | 0.0 | 0.833333 |
|  | A\_13 | 0.833333 | 0.0 | 0.166667 | 0.0 | 0.0 | 0.0 | 0.0 | 0.000000 | 1.000000 | 0.000000 | ... | 0.0 | 1.000000 | 0.000000 | 0.0 | 0.0 | 0.000000 | 0.000000 | 0.0 | 0.0 | 1.000000 |

5 rows × 49 columns

In [33]:

```
mConfDf.head()
```

Out[33]:

|  |  | AN | | | | | | | DI | | | ... | SA | | | SP | | | | | | |
| --- | --- | --- | --- | --- | --- | --- | --- | --- | --- | --- | --- | --- | --- | --- | --- | --- | --- | --- | --- | --- | --- | --- |
|  |  | ANG | DIS | FEA | HAP | NTR | SAD | SUP | ANG | DIS | FEA | ... | NTR | SAD | SUP | ANG | DIS | FEA | HAP | NTR | SAD | SUP |
|  | pNum |  |  |  |  |  |  |  |  |  |  |  |  |  |  |  |  |  |  |  |  |  |
| A | A\_1 | 1.000000 | 0.000000 | 0.0 | 0.0 | 0.0 | 0.0 | 0.0 | 0.166667 | 0.833333 | 0.000000 | ... | 0.000000 | 0.833333 | 0.000000 | 0.0 | 0.000000 | 0.166667 | 0.000000 | 0.0 | 0.0 | 0.833333 |
|  | A\_10 | 1.000000 | 0.000000 | 0.0 | 0.0 | 0.0 | 0.0 | 0.0 | 0.000000 | 0.666667 | 0.166667 | ... | 0.000000 | 0.833333 | 0.000000 | 0.0 | 0.166667 | 0.333333 | 0.000000 | 0.0 | 0.0 | 0.500000 |
|  | A\_11 | 0.833333 | 0.166667 | 0.0 | 0.0 | 0.0 | 0.0 | 0.0 | 0.000000 | 0.833333 | 0.000000 | ... | 0.000000 | 0.666667 | 0.000000 | 0.0 | 0.000000 | 0.000000 | 0.000000 | 0.0 | 0.0 | 1.000000 |
|  | A\_12 | 0.500000 | 0.500000 | 0.0 | 0.0 | 0.0 | 0.0 | 0.0 | 0.000000 | 0.666667 | 0.000000 | ... | 0.000000 | 0.500000 | 0.333333 | 0.0 | 0.000000 | 0.000000 | 0.166667 | 0.0 | 0.0 | 0.833333 |
|  | A\_13 | 0.833333 | 0.166667 | 0.0 | 0.0 | 0.0 | 0.0 | 0.0 | 0.000000 | 0.833333 | 0.000000 | ... | 0.166667 | 0.500000 | 0.000000 | 0.0 | 0.000000 | 0.000000 | 0.000000 | 0.0 | 0.0 | 1.000000 |

5 rows × 49 columns

## Plotting of all responses (incl. confusions)¶

In [34]:

```
stackColors = sns.color_palette("Set1", 7)
sns.palplot(stackColors)
```

In [35]:

```
#http://stackoverflow.com/a/214657
def rgb2hex(rgb):
    return '#%02x%02x%02x' % rgb
```

In [36]:

```
def makeConfPlot(confDf,group,count):
    
    # we have 3 subplots here
    ax = plt.subplot(1,3,count)

    # order of face expressions is defined by hand here, instead of
    # using the columns, so the order is as we wish it to be
    faceExpressions = ['HA','NE','SP','AN','DI','SA','FE']
    faceAnswers = ['HAP','NTR','SUP','ANG','DIS','SAD','FEA']
    
    # we loop through all expressions
    for i,emo in enumerate( faceExpressions ):
        
        # since we build a stacked plot, we have to initialize a value that
        # tells us where to start. We start at the very bottom, hence 0
        sumSoFar = 0
        
        # we get the values of all conditions, and their names, as defined
        # in the index
        thisMean = confDf.ix[group][emo].mean()
        thisIndex = thisMean.index
        
        # firstly, we are interested in the correct response, which will be
        # always at the bottom of the stack plot and printed in a strong color
        
        # corrPos is the position of the current expression in the list of all expressions
        corrPos = faceExpressions.index(emo)
        # and we also get the name of the answer at this position (cave: the order of lists
        # must match!)
        corrEntry = faceAnswers[corrPos]

        # we get the positions of all the other answers
        incorrAnswers = faceAnswers[:corrPos]+faceAnswers[corrPos+1:]
        
        # we get the value of the correct answer
        entry = thisMean[corrEntry]
        # we get the name of the correct answer
        answer = faceAnswers[corrPos]
        
        # we take the color list and extract the color for the correct position
        thisColor = stackColors[corrPos]
        thisHex = rgb2hex( (thisColor[0]*255,thisColor[1]*255,thisColor[2]*255) )
        # the colors for the remaining 7-1 colors are stored here:
        incorrColors = stackColors[:corrPos]+stackColors[corrPos+1:]
        
            
        # this is the first bar we generate, it starts at the bottom (sumSoFar=0) and
        # has a strong color (alpha=1)
        ax.bar(i,
                entry,
                bottom=sumSoFar,
                color=thisHex,
                label=answer,
                alpha=1.)      
        
        # to stack the rest of the bars on top, we update the sum to be
        # the value (% correct) of the current condition
        sumSoFar = entry
        
        # we loop through the remaining (incorrect answers)
        for j,answer in enumerate(incorrAnswers):
            entry = thisMean[answer]

            thisColor = incorrColors[j]
            thisHex = rgb2hex( (thisColor[0]*255,thisColor[1]*255,thisColor[2]*255) )
            
            # same thing as above, but without the legend and with transparency
            ax.bar(i,
                    entry,
                    bottom=sumSoFar,
                    color=thisHex,
                    alpha=0.5
                        )           
            # for each run of the loop, we update the sumSoFar so we
            # always stack each expression at the top
            sumSoFar+=entry
    
    # get rid of figure box
    sns.despine()
    
    # x labels for each plot
    ax.set_xlabel('Basic Expression',fontsize=14)
    # y labels only for the first plot
    if i ==0:
        ax.set_ylabel('% of responses')
    else:
        ax.set_ylabel('')

    # axes annotation for all plots
    plt.xticks(np.arange(0,7.1,1)+0.4, faceExpressions,fontsize=12)
    plt.yticks(np.arange(0,1.01,0.1), [str(a)+'%' for a in  np.arange(0,101,10)],fontsize=14)

    ax.set_ylim(0,1)
    
    # getting the name of each subplot right
    groupNames = {'A':'General Population',
                  'G':'Violence Offenders',
                  'K':'Child Molesters'
                 }
    ax.set_title(groupNames[group],position=(0.5,1.03) )
    
    # legend only at the very end (3rd plot)
    if count == 3:
        plt.legend(loc='best',bbox_to_anchor=[1,1])
```

In [37]:

```
def makeAllConfPlots(confDf):
    
    # loop through male and female faces
    for fGender in confDf.columns.levels[0][::-1]:
        
        plt.figure(figsize=(16,4))
        thisDf = confDf[fGender]
        
        # loop through participant groups
        for i,group in enumerate( ['G','K','A'] ):
            makeConfPlot(thisDf,group,i+1)

        # each plot gets a title...
        titleDict = {'M':'Male Faces','F':'Female Faces'}
        plt.suptitle(titleDict[fGender], position=(0.5, 1.1),fontsize=20)
        # ...and is saved seperately under a differnt name
        plt.savefig('../figures/basicEmoPlot_'+fGender+'.png',
                    dpi=300,
                    bbox_inches='tight')
        # show in notebook
        plt.show()
```

In [38]:

```
makeAllConfPlots(confDf)
```

## Getting a global emo score for each participant¶

This averages all correct responses, so we have one value per participant, which indicates percentage of correct responses overall.

In [39]:

```
bigDf.head()
```

Out[39]:

|  | cond | AN | DI | FE | HA | NE | SA | SP |
| --- | --- | --- | --- | --- | --- | --- | --- | --- |
| group | id |  |  |  |  |  |  |  |
| A | A\_10 | 1.000000 | 0.750000 | 0.416667 | 1.000000 | 0.916667 | 0.916667 | 0.500000 |
|  | A\_11 | 0.916667 | 0.916667 | 0.833333 | 1.000000 | 0.666667 | 0.666667 | 0.916667 |
|  | A\_12 | 0.666667 | 0.666667 | 0.083333 | 1.000000 | 0.916667 | 0.750000 | 0.833333 |
|  | A\_13 | 0.833333 | 0.916667 | 0.416667 | 1.000000 | 0.916667 | 0.750000 | 1.000000 |
|  | A\_14 | 1.000000 | 0.833333 | 0.250000 | 0.916667 | 1.000000 | 0.750000 | 0.916667 |

In [40]:

```
globalCorrect = pd.DataFrame( bigDf.mean(axis=1) )
globalCorrect['group'] = [ labelCoding[x[0]] for x in globalCorrect.index.levels[1] ]
globalCorrect = globalCorrect.sort_values(by='group')
```

In [41]:

```
globalCorrect.head()
```

Out[41]:

|  |  | 0 | group |
| --- | --- | --- | --- |
| group | id |  |  |
| G | G\_22 | 0.654762 | 0 |
|  | G\_16 | 0.607143 | 0 |
|  | G\_17 | 0.761905 | 0 |
|  | G\_18 | 0.738095 | 0 |
|  | G\_19 | 0.821429 | 0 |

### Plot¶

In [42]:

```
fig = plt.figure(figsize=(7,7))

sns.boxplot(x='group',y=0,data=globalCorrect,
            width=0.4,linewidth=1,color='white',whis=True,notch=True,fliersize=0)

sns.stripplot(x=globalCorrect['group'],y=globalCorrect[0],
              jitter=True,palette=stackColors)
sns.despine()
plt.ylim(0.5,1.);
```

### Make correct responses for male and female faces for 2x3x7 ANOVA in JASP¶

In [43]:

```
faceList = list(confDf.columns.levels[1])
respList = list(confDf.columns.levels[2])

jaspDf = pd.DataFrame()

for fGender in confDf.columns.levels[0]:

    for face in faceList:
        for resp in respList:
            if faceList.index(face) == respList.index(resp):
                thisDf =  confDf[fGender][face][resp] 
                thisDf.name = fGender+'_'+thisDf.name
                jaspDf = pd.concat([jaspDf,thisDf ],axis=1)
                
jaspDf['group'] = [ x[0] for x in jaspDf.index.levels[1]]
```

In [44]:

```
jaspDf.head()
```

Out[44]:

|  |  | F\_ANG | F\_DIS | F\_FEA | F\_HAP | F\_NTR | F\_SAD | F\_SUP | M\_ANG | M\_DIS | M\_FEA | M\_HAP | M\_NTR | M\_SAD | M\_SUP | group |
| --- | --- | --- | --- | --- | --- | --- | --- | --- | --- | --- | --- | --- | --- | --- | --- | --- |
|  | pNum |  |  |  |  |  |  |  |  |  |  |  |  |  |  |  |
| A | A\_1 | 1.000000 | 0.833333 | 0.500000 | 1.0 | 0.166667 | 0.500000 | 0.833333 | 1.000000 | 0.833333 | 0.333333 | 1.0 | 0.500000 | 0.833333 | 0.833333 | A |
|  | A\_10 | 1.000000 | 0.833333 | 0.166667 | 1.0 | 1.000000 | 1.000000 | 0.500000 | 1.000000 | 0.666667 | 0.666667 | 1.0 | 0.833333 | 0.833333 | 0.500000 | A |
|  | A\_11 | 1.000000 | 1.000000 | 0.833333 | 1.0 | 0.833333 | 0.666667 | 0.833333 | 0.833333 | 0.833333 | 0.833333 | 1.0 | 0.500000 | 0.666667 | 1.000000 | A |
|  | A\_12 | 0.833333 | 0.666667 | 0.166667 | 1.0 | 1.000000 | 1.000000 | 0.833333 | 0.500000 | 0.666667 | 0.000000 | 1.0 | 0.833333 | 0.500000 | 0.833333 | A |
|  | A\_13 | 0.833333 | 1.000000 | 0.500000 | 1.0 | 0.833333 | 1.000000 | 1.000000 | 0.833333 | 0.833333 | 0.333333 | 1.0 | 1.000000 | 0.500000 | 1.000000 | A |

In [45]:

```
jaspDf.to_csv('../outputs/basicExpressionsJASP.csv')
```
