# Supplementary material for: In your face: the biased judgement of fear-anger expressions in violent offenders
Source: BMC Psychol. 2017 May 12;5:16. doi: 10.1186/s40359-017-0186-z (PMC5429544; doi:10.1186/s40359-017-0186-z)
Supplement: Supplementary file 7 — Data parsing for morph experiment. (HTML 232 kb) [file 40359_2017_186_MOESM7_ESM.html]

003\_gettingMorphData


# In your face: Biased judgement of fear-anger expressions in violence offenders.

# 3. Extracting Morph data from Logfiles¶

This notebook extracts the data from the main experiment, i.e. the face morphing

### import libraries¶

In [1]:

```
import numpy as np
import pandas as pd

import os
import fnmatch

from myBasics import *
```

### get logfiles¶

In [2]:

```
def get_logfile(whichfolder, whichexperiment):

    loglist = []
    for fileName in os.listdir(whichfolder):
        if fnmatch.fnmatch(fileName, whichexperiment):
            loglist.append(whichfolder+fileName)
    return loglist
```

In [3]:

```
loglist = get_logfile('../experiment/data/','*facesParametric*.csv')
loglist.sort()
```

Example:

In [4]:

```
loglist[:5]
```

Out[4]:

```
['../experiment/data/A_10_facesParametric_2015_Sep_20_1307.csv',
 '../experiment/data/A_11_facesParametric_2015_Sep_25_1753.csv',
 '../experiment/data/A_12_facesParametric_2015_Oct_11_1221.csv',
 '../experiment/data/A_13_facesParametric_2015_Oct_11_1620.csv',
 '../experiment/data/A_14_facesParametric_2015_Nov_01_1250.csv']
```

In [5]:

```
def getMorphResps(fileName):
    fullDf = pd.read_csv(fileName)
    
    # boil down to just the essentials
    thisDf = pd.concat([fullDf['img'],
                    fullDf['mouseResp.leftButton'],
                    fullDf['mouseResp.rightButton'],
                    fullDf['mouseResp.time']
                   ],axis=1)
    
    # we output starting at row 89, because the previous
    # rows belong the previous experiment (basic expression recognition)
    return thisDf[89:]
```

Example:

In [6]:

```
getMorphResps(loglist[-1]).head()
```

Out[6]:

|  | img | mouseResp.leftButton | mouseResp.rightButton | mouseResp.time |
| --- | --- | --- | --- | --- |
| 89 | ./stim/MorphM9\_07\_orig.jpg | 1.0 | 0.0 | 3.044200 |
| 90 | ./stim/MorphM10\_05\_orig.jpg | 0.0 | 1.0 | 1.925446 |
| 91 | ./stim/MorphM2\_07\_orig.jpg | 0.0 | 1.0 | 1.942962 |
| 92 | ./stim/MorphF5\_06\_orig.jpg | 0.0 | 1.0 | 1.305991 |
| 93 | ./stim/MorphM3\_09\_orig.jpg | 0.0 | 1.0 | 2.845542 |

In [7]:

```
def getCondition(stimList):

    part = []
    grades = []
    genders = []
    idents = []
    pt = 1

    for entry in stimList:

        try:
            grade = entry[entry.find('_')+1:entry.rfind('_')]
            ident = entry[entry.find('Morph')+len('Morph'):entry.find('_')]
            gender = entry[entry.find('Morph')+len('Morph'):entry.find('Morph')+len('Morph')+1]
            grades.append(grade)
            idents.append(ident+'_'+str(pt))
            genders.append(gender)
            part.append(pt)

        except:
            grades.append(float(np.nan))
            idents.append(float(np.nan))
            genders.append(float(np.nan))
            part.append(float(np.nan))
            pt = 2
            
    return part,grades,genders,idents
```

In [8]:

```
def makeTable(fileName):
    thisDf = getMorphResps(fileName)
    # get the file names from which the conditions are extracted
    stimList = thisDf['img']
    # 
    parts,grades,genders,idents = getCondition(stimList)
    # apply 
    #thisDf['part'] = parts
    thisDf['grades'] = grades
    thisDf['gender'] = genders
    thisDf['idents'] = idents
    # sort
    #thisDf = thisDf.sort(['part','gender','grades','idents'])
    thisDf = thisDf.sort_values(by=['grades','gender'])
    # set index
    #thisDf = thisDf.set_index(['part','gender','grades'])
    thisDf = thisDf.set_index(['grades','gender','idents'])
    # drop nan
    thisDf =  thisDf.dropna()
    
    return thisDf
```

Example:

In [9]:

```
makeTable(loglist[-1]).head()
```

Out[9]:

|  |  |  | img | mouseResp.leftButton | mouseResp.rightButton | mouseResp.time |
| --- | --- | --- | --- | --- | --- | --- |
| grades | gender | idents |  |  |  |  |
| 00 | F | F10\_1 | ./stim/MorphF10\_00\_orig.jpg | 1.0 | 0.0 | 2.763731 |
|  |  | F2\_1 | ./stim/MorphF2\_00\_orig.jpg | 1.0 | 0.0 | 3.099604 |
|  |  | F3\_1 | ./stim/MorphF3\_00\_orig.jpg | 1.0 | 0.0 | 0.701015 |
|  |  | F9\_1 | ./stim/MorphF9\_00\_orig.jpg | 1.0 | 0.0 | 2.060379 |
|  |  | F6\_1 | ./stim/MorphF6\_00\_orig.jpg | 1.0 | 0.0 | 1.154952 |

In [10]:

```
def cleanAvg(fileName):

    fileNum = fileName[fileName.find('_')+1:fileName.find('faces')-1]
    
    if int(fileNum)%2 == 0:
        targetButton = 'mouseResp.leftButton'
        nonTargetButton = 'mouseResp.rightButton'
    else:
        targetButton = 'mouseResp.rightButton'
        nonTargetButton = 'mouseResp.leftButton'
        

    pName = (fileName[fileName.rfind('/')+1:fileName.find('_')]+ ('000'+fileNum)[-3:])
    
    thisDf = makeTable(fileName)
    
    # get rid of everything but
    thisDf = thisDf.drop('img',1)
    thisDf = thisDf.drop(nonTargetButton,1)
    thisDf = thisDf.drop('mouseResp.time',1)
    
    # restructure for gender-based averaging
    thisDfWithin = thisDf.unstack(0)
    thisDfWithin.columns = thisDfWithin.columns.droplevel()

    # average by gender
    fDf = pd.DataFrame( thisDfWithin.ix['F'].mean(),columns=['F'] )
    mDf = pd.DataFrame( thisDfWithin.ix['M'].mean(),columns=['M'] )
    
    # restructure
    avgDf = pd.concat([fDf,mDf],axis=1).T
    # nice indexing
    avgDf.index = [ [pName[:1]]*len(avgDf.index),
                    [pName]*len(avgDf.index),
                    avgDf.index
                  ]
    avgDf['group'] = [ labelCoding[avgDf.index.levels[0][-1]], labelCoding[avgDf.index.levels[0][-1] ]  ]
    
    return avgDf
```

Example:

In [11]:

```
cleanAvg(loglist[0])
```

Out[11]:

|  |  | grades | 00 | 01 | 02 | 03 | 04 | 05 | 06 | 07 | 08 | 09 | 10 | group |
| --- | --- | --- | --- | --- | --- | --- | --- | --- | --- | --- | --- | --- | --- | --- |
| A | A010 | F | 0.05 | 0.05 | 0.05 | 0.10 | 0.25 | 0.50 | 0.60 | 0.80 | 0.85 | 0.95 | 1.0 | 2 |
|  |  | M | 0.05 | 0.05 | 0.05 | 0.25 | 0.40 | 0.35 | 0.75 | 0.85 | 0.95 | 1.00 | 1.0 | 2 |

In [12]:

```
def makeBigOne(allFiles):
    
    for fileName in allFiles:

        thisDf = cleanAvg(fileName)
        
        try:
            bigDf = pd.concat([bigDf,thisDf])
        except:
            bigDf = thisDf
    
    bigDf.index.names = ['g','p','fgender']
    
    bigDf = bigDf.sort_index()

    return bigDf
```

### Make a comprehensive table, where male and female face identities are treated seperately¶

In [13]:

```
bigDf = makeBigOne(loglist)
```

In [14]:

```
bigDf.tail()
```

Out[14]:

|  |  | grades | 00 | 01 | 02 | 03 | 04 | 05 | 06 | 07 | 08 | 09 | 10 | group |
| --- | --- | --- | --- | --- | --- | --- | --- | --- | --- | --- | --- | --- | --- | --- |
| g | p | fgender |  |  |  |  |  |  |  |  |  |  |  |  |
| K | K013 | M | 0.25 | 0.50 | 0.40 | 0.55 | 0.60 | 0.55 | 0.80 | 0.80 | 1.00 | 0.90 | 1.00 | 1 |
|  | K014 | F | 0.00 | 0.00 | 0.05 | 0.00 | 0.00 | 0.25 | 0.45 | 0.70 | 0.85 | 0.95 | 0.95 | 1 |
|  |  | M | 0.20 | 0.20 | 0.30 | 0.35 | 0.35 | 0.40 | 0.45 | 0.65 | 0.75 | 0.90 | 0.85 | 1 |
|  | K015 | F | 0.10 | 0.15 | 0.20 | 0.20 | 0.30 | 0.45 | 0.70 | 0.75 | 0.90 | 1.00 | 0.95 | 1 |
|  |  | M | 0.00 | 0.00 | 0.05 | 0.20 | 0.35 | 0.30 | 0.65 | 0.70 | 0.85 | 1.00 | 1.00 | 1 |

In [15]:

```
bigDf.to_csv('../outputs/genderTable.csv')
```

### Restructure to run an ANOVA in JASP¶

In [16]:

```
bigDfUnstacked = bigDf[bigDf.columns[:-1]].unstack(2).stack(0).unstack(2)
```

In [17]:

```
bigJasp = bigDfUnstacked.copy()
myColumns = [str(x[0])+'_'+str(x[-1]) for x in bigDfUnstacked.columns]
bigJasp.columns = myColumns
```

In [18]:

```
bigJasp.head()
```

Out[18]:

|  |  | F\_00 | F\_01 | F\_02 | F\_03 | F\_04 | F\_05 | F\_06 | F\_07 | F\_08 | F\_09 | ... | M\_01 | M\_02 | M\_03 | M\_04 | M\_05 | M\_06 | M\_07 | M\_08 | M\_09 | M\_10 |
| --- | --- | --- | --- | --- | --- | --- | --- | --- | --- | --- | --- | --- | --- | --- | --- | --- | --- | --- | --- | --- | --- | --- |
| g | p |  |  |  |  |  |  |  |  |  |  |  |  |  |  |  |  |  |  |  |  |  |
| A | A001 | 0.00 | 0.10 | 0.10 | 0.20 | 0.15 | 0.3 | 0.85 | 0.90 | 0.90 | 1.00 | ... | 0.10 | 0.20 | 0.20 | 0.25 | 0.50 | 0.70 | 0.85 | 0.95 | 1.00 | 1.00 |
|  | A002 | 0.20 | 0.10 | 0.05 | 0.30 | 0.25 | 0.5 | 0.75 | 0.90 | 0.75 | 1.00 | ... | 0.25 | 0.35 | 0.20 | 0.35 | 0.55 | 0.60 | 0.90 | 0.85 | 1.00 | 1.00 |
|  | A003 | 0.00 | 0.05 | 0.15 | 0.10 | 0.30 | 0.4 | 0.55 | 0.65 | 0.65 | 0.65 | ... | 0.00 | 0.05 | 0.05 | 0.30 | 0.25 | 0.45 | 0.50 | 0.80 | 0.95 | 0.95 |
|  | A004 | 0.45 | 0.35 | 0.40 | 0.30 | 0.40 | 0.6 | 0.30 | 0.45 | 0.45 | 0.60 | ... | 0.30 | 0.35 | 0.35 | 0.40 | 0.45 | 0.35 | 0.30 | 0.45 | 0.50 | 0.50 |
|  | A005 | 0.35 | 0.20 | 0.15 | 0.55 | 0.40 | 0.7 | 0.90 | 1.00 | 1.00 | 1.00 | ... | 0.20 | 0.30 | 0.45 | 0.40 | 0.65 | 0.90 | 1.00 | 1.00 | 1.00 | 1.00 |

5 rows × 22 columns

In [19]:

```
bigJasp.to_csv('../outputs/genderMorphsJASP.csv')
```

### Average over male and female face identities (one value per participant)¶

In [20]:

```
def makeAverage(bigDf):
    
    meanDf = pd.DataFrame()
    
    for entry in bigDf.index.levels[1]:
        thisRow = pd.DataFrame( list(bigDf.ix[entry[0]].ix[entry].mean()) ).T
        thisRow.index = [entry]
        meanDf = pd.concat([meanDf,thisRow])   

    meanDf.index.name = 'p'
    meanDf.index = [ [str(x)[0] for x in list(meanDf.index) ] ,meanDf.index]
    
    return meanDf[meanDf.columns[:-1]]
```

In [21]:

```
meanDf = makeAverage(bigDf)
```

In [22]:

```
meanDf.head()
```

Out[22]:

|  |  | 0 | 1 | 2 | 3 | 4 | 5 | 6 | 7 | 8 | 9 | 10 |
| --- | --- | --- | --- | --- | --- | --- | --- | --- | --- | --- | --- | --- |
|  | p |  |  |  |  |  |  |  |  |  |  |  |
| A | A001 | 0.000 | 0.100 | 0.150 | 0.200 | 0.2 | 0.400 | 0.775 | 0.875 | 0.925 | 1.00 | 1.000 |
|  | A002 | 0.125 | 0.175 | 0.200 | 0.250 | 0.3 | 0.525 | 0.675 | 0.900 | 0.800 | 1.00 | 1.000 |
|  | A003 | 0.000 | 0.025 | 0.100 | 0.075 | 0.3 | 0.325 | 0.500 | 0.575 | 0.725 | 0.80 | 0.900 |
|  | A004 | 0.400 | 0.325 | 0.375 | 0.325 | 0.4 | 0.525 | 0.325 | 0.375 | 0.450 | 0.55 | 0.625 |
|  | A005 | 0.275 | 0.200 | 0.225 | 0.500 | 0.4 | 0.675 | 0.900 | 1.000 | 1.000 | 1.00 | 1.000 |

In [23]:

```
meanDf.to_csv('../outputs/meanMorphsTable.csv')
```
